# “Involuntary” and “Voluntary” in Psychiatric, Behavioral, and Mental Health Services: A Scoping Review of Definitions

**DOI:** 10.1007/s11414-025-09940-8

**Published:** 2025-03-26

**Authors:** Benjamin D. Smart, Kritheeka Kalathil, William V. McCall, Sahil Munjal, Haley Kirkendall, Madison Patel, Amy Taliaferro, Lauren H. Yaeger, Ana S. Iltis

**Affiliations:** 1 Division of Psychology, Department of Clinical Neuroscience, Karolinska Institutet, Nobels väg 9, Stockholm, 17165 Sweden; 2 Wake Forest University Center for Bioethics, Health and Society, Wake Forest University, 1834 Wake Forest Rd., Winston-Salem, NC 27106 USA; 3 Sandra and Leon Levine Psychiatry Residency Program, Carolinas Medical Center, 501 Billingsley Rd, Charlotte, NC 28211 USA; 4 Department of Psychiatry and Health Behavior, Medical College of Georgia, Augusta University, Augusta, GA USA; 5 Wake Forest School of Medicine, Department of Psychiatry and Behavioral Medicine, 791 Jonestown Road, Winston-Salem, NC 27103 USA; 6 Wake Forest Psychiatry and Behavioral Medicine Residency Program, Atrium Health Wake Forest Baptist, 791 Jonestown Road, Winston-Salem, NC 27103 USA; 7 Wake Forest University, 1834 Wake Forest Rd., Winston-Salem, NC 27106 USA; 8 Wake Forest University School of Law, 1834 Wake Forest Rd, Winston-Salem, NC 27109 USA; 9 Becker Medical Library, Washington University School of Medicine, Campus Box 8132, 660 S. Euclid Ave., St. Louis, MO 63110 USA; 10 Department of Philosophy, Wake Forest University, 1834 Wake Forest Rd, Winston-Salem, NC 27106 USA

**Keywords:** Involuntary, Voluntary, Civil commitment, Psychiatry, Health services, Ethics

## Abstract

The meaning of (in)voluntary in the context of psychiatric, behavioral, and mental health services in the United States lacks shared understanding despite widespread use, such as in “involuntary treatment,” “involuntary hospitalization,” and “voluntary patient.” A pre-registered scoping review was conducted to describe how U.S.-based healthcare professionals explicitly define (in)voluntary when referring to psychiatric, behavioral, and mental health concepts. Nine databases and nine organization/government websites were searched. Eligibility criteria included English availability, U.S.-based healthcare author(s), and an explicit definition of (in)voluntary. Extracted data included study characteristics and (in)voluntary term(s) with definitions. Open coding was used for the (in)voluntary-associated definition. Meaning categories were created by grouping codes. A total of 29,313 citations were screened for a final 162 sources, containing 203 definitions. Evidence sources (years 1966–2023) were most frequently research articles (33%), review articles (24%), and books (20%) with authorship including persons with an M.D./D.O. (70%) and/or Ph.D. (51%). The most common definition words were order, coercion, against/opposed to a patient’s will/wishes, consent, force, and adhere. Meaning categories were external pressure, civil rights, individual agency, competence and capacity, and ethics. Involuntary definitions more commonly characterized the patient as actively against an intervention (23%), rather than without active agreement (11%). Some definitions included a legal (62%) and/or ethical dimension (33%). Two-thirds of sources used at least one additional (in)voluntary term in the publication without defining it. Because there exists a range of competing definitions for the term (in)voluntary, authors who use this descriptor clearly are recommended to explain their meaning.

**Supplementary Information:**

The online version contains supplementary material available at 10.1007/s11414-025-09940-8.

## Introduction

In psychiatric, mental health, and behavioral health services in the United States, “voluntary” and “involuntary” are used as descriptors for a range of intervention-related concepts, such as “involuntary treatment,” “involuntary commitment,” and “hospitalized involuntarily.” Yet the communicator’s precise meaning of (in)voluntary can be unclear despite the widely understood lexical definitions of “voluntary” and “involuntary.” *Meaning*, from semantics, refers to the relationships between the world, language, and communicators ^1^. In text, definitions are one important and sometimes necessary way to communicate meaning.

Consider the statement: “The patient was involuntarily hospitalized.” Among the possible interpretations, the writer could mean: (1) the patient was hospitalized against their will (i.e., contrary to a desire to not be hospitalized) as a form of coercion, (2) hospitalization of a patient who is clinically perceived to not have the decision-making capacity to consent to a hospitalization but did not actively resist, or (3) the patient underwent the legal process of civil commitment without making assertions about their level of willingness and/or consent.

Recognizing this ambiguity is not new. A late 1970s physician-authored paper began, “In the continuing debate over involuntary psychiatric hospitalization, relatively little attention has been given to defining the central concepts ‘voluntary’ and ‘involuntary’” ^2^^(p. 104)^. In 1986, an involuntary treatment coordinator wrote a letter to the editor responding to articles debating involuntary treatment: “Neither side questioned the construct of involuntary treatment itself — what, in fact, involuntary treatment means. I see the lack of discussion of this issue as a significant omission” ^3^^(p. 184)^. In 2003, a physician noted that “what defines ‘voluntary’ versus ‘involuntary' care remains ‘an unresolved question ’” ^4^^(p. 60)^. Others have also pointed out confusion around the terms involuntary treatment ^5^ and involuntary commitment ^6^. The issue is such that when leading psychiatric emergency medicine physicians (M.D. or D.O.) responded to survey questions involving their personal definition of “voluntary” and “involuntary” emergency medication, most, but not all, answered that any oral medication that the patient assents to in emergencies is considered voluntary and not coercive enough to be involuntary ^7^. Underscoring the importance of reaching clarity on terminology, the survey was undertaken as part of developing expert consensus guidelines in managing behavioral health emergencies with goals that included improving clinical decision-making, standardizing best practices, and ensuring ethical and patient-centered approaches. As recent as 2022, psychiatrist research authors have pointed out that involuntary hospitalization referring to the legal status of the patient may have little to do with the patient’s actual level of voluntariness, indicating that the terminology confusion has not been adequately resolved ^8^.

Others argue that the term involuntary is used too broadly and current applications miss important nuances ^9^. An additional category of “nonvoluntary” has received attention as a way to describe clinical situations where the “patient exists in an intermediate domain of decision-making capacity and voluntariness” ^10^. Patients, family members, journalists, and other members of the public suffer from the lack of clarity. The non-profit Treatment Advocacy Center reported in 2020 that it frequently receives questions about involuntary treatment, notably due to lack of consistent terminology ^11^.

Opaqueness surrounding the word “involuntary” in behavioral and mental healthcare has created problems in clinical care and research. A lack of shared meaning can create conflict between healthcare teams, patients, their families, and the legal system, particularly during emergency mental health situations where decisions are made expeditiously for the safety of the patient or others. For example, a patient deemed to have a mental health diagnosis, practitioner-assessed low insight, and treatment non-adherence may meet involuntary commitment criteria to a healthcare facility; however, involuntary treatment may not necessarily be provided unless certain safety or legal criteria are satisfied. A misunderstanding could lead to a violation of the patient’s rights and/or distrust between patients and healthcare teams. An overly broad understanding of involuntariness may lead to providing similar treatment for situations that differ in ethically important ways. As an administrative and legal status, the “involuntary” label can have important implications for a patient’s healthcare cost liability, future employment, professional licensing, child custody, access to firearms, admission to hospitals, and access to scarce psychiatric resources.

From a research perspective, patient records containing the “involuntary” label can restrict access to data for confidentiality protections, underscoring the importance of precise clinical care terminology. Within epidemiology, variation in the definitions of involuntary civil commitment across U.S. states has been a significant barrier to measurement ^12^. Studies of healthcare data across different hospitals, states, or other contexts may be unreliable due to the introduction of error stemming from different local and legal meanings of (in)voluntary. The critical importance of clarity can be stated this way: Clinical decisions regarding involuntary treatment are usually guided by state laws and hospital policies, which in turn are (hopefully) based on research-generated evidence, which relies on clear and consistent labels for what constitutes (in)voluntary interventions.

As a step toward addressing these problems, a scoping review was conducted to document meanings of “involuntary” and “voluntary” in psychiatric, behavioral, and mental healthcare in the United States. The primary objective was to develop a comprehensive description of the ways U.S.-based healthcare professionals define (in)voluntary when referring to concepts that involve the practice of psychiatry or that concern psychiatric disorders, mental health, or behavioral health.

## Methods

A scoping review was chosen as it is suited for research questions that map the breadth of available evidence and clarify key concepts ^13^. Methods were based on recommendations from JBI (previously: Joanna Briggs Institute), ^14^ and reporting follows the Preferred Reporting Items for Systematic Reviews and Meta-Analyses Extension for Scoping Reviews (PRISMA-ScR) ^15^. A scoping review protocol was pre-registered in the Open Science Framework (OSF) [registration DOI: https://doi.org/10.17605/OSF.IO/6BJ8Y] ^16^.

### Research Question

The research question that this review addressed was: How do U.S.-based healthcare professionals define the term “involuntary” explicitly when referring to interventions or decisions that involve the practice of psychiatry or that concern psychiatric disorders, mental health, or behavioral health?

### Eligibility Criteria

The detailed inclusion and exclusion criteria—organized by the population, concept, and context framework—and the exclusion hierarchy are available in the supplemental results. Briefly, evidence sources were included if they had: (1) English availability, (2) authors included a U.S.-based healthcare worker (e.g., doctor, nurses psychologist, counselor, or social worker) or physician or psychological professional organization, (3) full-text availability, (4) contained the word “involuntary” or iteration of the lexeme (a single word in all its grammatical variants) “voluntary” describing U.S.-based care in the title, abstract, or main text, and (5) contained an explicitly clear definition of (in)voluntary as it applies to concepts involving psychiatry, mental health, or behavioral health. There were no time restrictions as we wanted to capture as many definitions as possible, and thus sources from all years available in the databases were included. A definition was explicitly clear when it was a “dictionary-type” definition, for example, “involuntary hospitalization is defined as ____,” “involuntary treatment, which is _____,” and “involuntary hold, _____.” Definitions implied from the text and *definition by typical exemplar* were excluded to maximize precision, minimize subjectivity, and keep the review scope sufficiently narrow to document differences. To be clear, the “involuntary” concept often includes mention of the law and is thus of interest, but we did not include sources authored by legal professionals without any clinician coauthors, as those were deemed to be a separate project outside the scope. Patient (in)voluntary movements, in the context of cortical control and dyskinesias, and (in)voluntary thoughts were excluded. It was decided to limit the scope to the United States because (in)voluntary psychiatric care is closely tied to laws which differ drastically around the world ^17^. Despite U.S. state law variation, there are relevant federal laws and definitions that are shared among states.

### Information Sources and Search

A medical librarian (LHY) searched the literature for records including the concepts of involuntary commitment, psychiatry, psychiatric disorders, mental health, behavioral health, intervention, hospitalization, outpatient treatment, and medication. The librarian created search strategies using a combination of keywords and controlled vocabulary in Embase.com (1947–present), Ovid Medline (1946–present), Scopus (1823–present), Cochrane Central Register of Controlled Trials, The Cochrane Database of Systematic Reviews, Cumulative Index to Nursing and Allied Health Literature Plus (1937–present), APA PsycInfo (1800s–present), and Clinicaltrials.gov (1997–present). All search strategies were completed July 11, 2022, with no added limits, and a total of 29,313 results were found. 14,157 duplicate records were deleted after using de-duplication processes ^18^ resulting in a total of 15,156 unique citations in the project library. Fully reproducible search strategies are in the supplemental results.

The websites of nine groups were reviewed, with the final check in June 2023, to identify publicly available documents that addressed (in)voluntary concepts. These organizations/societies were chosen because they state they represent psychiatrists or other mental healthcare providers who deliver interventions that may be described as “involuntary”: American Psychiatric Association, American Psychological Association, Association for Psychological Science, Academy of Consult-Liaison Psychiatry, American Neuropsychiatric Association, American Academy of Addiction Psychiatry, Veterans Affairs/U.S. Department of Defense, American Academy of Psychiatry and the Law, and the National Association of Social Workers.

### Selection of Sources of Evidence

Two authors (BDS and ASI) separately reviewed all titles and abstracts for the first screening wherein only sources that did not clearly meet the inclusion criteria were excluded. Next, full-text review was divided among a review team (BDS, HRP, SD, AT, KK, GW, CD, and MP) and each publication was screened separately by two reviewers. An exclusion hierarchy, based on the highest-ranking inclusion criterion the source failed to meet, was developed and used. Discrepancies were resolved through discussion and consultation with other team members. Four reviewers (BDS, KK, HRP, and AT) searched the websites of the professional groups evidence sources that address involuntary interventions. These were assessed by two authors (BDS and ASI) for inclusion in the scoping review. Disagreements were resolved through discussion. Covidence software was used for review management.

### Data Charting: Data Items, Data Analysis, and Synthesis of Results

Following full-text review, data extraction was performed (BDS, HRP, and MP) using a data extraction template developed a priori. Extraction was done by one author, then checked by another.

The semantic theories that guided the approach to describing (in)voluntary’s meaning were *meaning as reference* and *meaning as concepts *^1^. Meaning as reference is the theory that a word is best understood by naming the specific thing to which it refers. Meaning as concepts refers to understanding a word through its associated words and expressions that refer to thoughts. To describe meaning, methods here focused on definitions of (in)voluntary in text. There are many types of definitions, but because the review’s aim focused on finding explicit, clear explanations of (in)voluntary that could be easily compared, the authors identified “dictionary-type” definitions, rather than “encyclopedic-type” definitions or definitions by context ^19^. The approach was from the perspective that (in)voluntary terms are compositional: the overall term definition could be broken down according to individual lexemes, i.e., a portion of the definition of “involuntary treatment,” for example, defines the “involuntary” aspect of the term ^1^.

Data was analyzed quantitatively and qualitatively. Descriptive statistics were calculated from article metadata. Conceptual qualitative content analysis was used to analyze the source text. Inductive analysis was used with open coding. Each definition’s code consisted of all key words in the definition that captured the relevant (in)voluntary component. The definition domain was defined as the individual involuntary-related code lexeme(s), phrase(s), or general concept. A three-column codebook was created with the (1) word form of voluntary and the code, (2) source ID (each source was assigned a unique number for this project) and any words in the defined term that are described or modified as (in)voluntary, and (3) code domain. Term synonyms were noted. The codebook and a summary table are available on OSF.

The codes were then grouped into overarching conceptual categories, which were developed inductively by the authors by analyzing broader ideas shared by the key definition words. When comparing (in)voluntary terms and definitions, terms needed to match almost exactly to be compared; for example, “involuntary treatment laws” was not considered the same as “involuntary treatment.” Motivated by arguments about distinguishing between nonvoluntary and involuntary treatment, ^8,9^ definitions were categorized for situations where patients are *actively against the intervention* (e.g., patient was “forced,” “against their will/wishes,” “despite refusal,” or “over objection”), versus situations where patients are *without active agreement* (e.g., without informed consent). Whole definitions were considered, and lenses for approaching the term were identified inductively by the authors (“definition dimensions”): ethics, legal, and safety. Ethics referred to explicit mention of an ethical concept (e.g., informed consent, coercion, and autonomy vs. beneficence), the specific definitions of which are outside the scope of this review. Legal included explicit mention of a legal, law, or justice concept (e.g., “legal status,” “civil commitment,” and “mental health courts”). Safety referred to mentions of personal safety or danger (e.g., “pose a danger to themselves or others” and “admitted for their own safety”). All definitions were re-read and coded for each dimension’s presence or absence.

SPSS version 28 was used for statistical analysis. All codes and protocol amendments are publicly available on OSF. Supplemental results available online include results describing (in)voluntary parts of speech, repeated definitions, and change over time comparisons.

### Data Availability Statement

The Online Supplement contains detailed inclusion and exclusion criteria with exclusion hierarchy, the full search strategy, the full citation list of included sources, and a table of data about the included sources. The OSF online repository (https://osf.io/grw7q/) contains the pre-registered protocol, qualitative codebook, data table for counting definition words, and analysis code for descriptive statistics.

## Results

### Scoping Review Process

**Figure 1:**
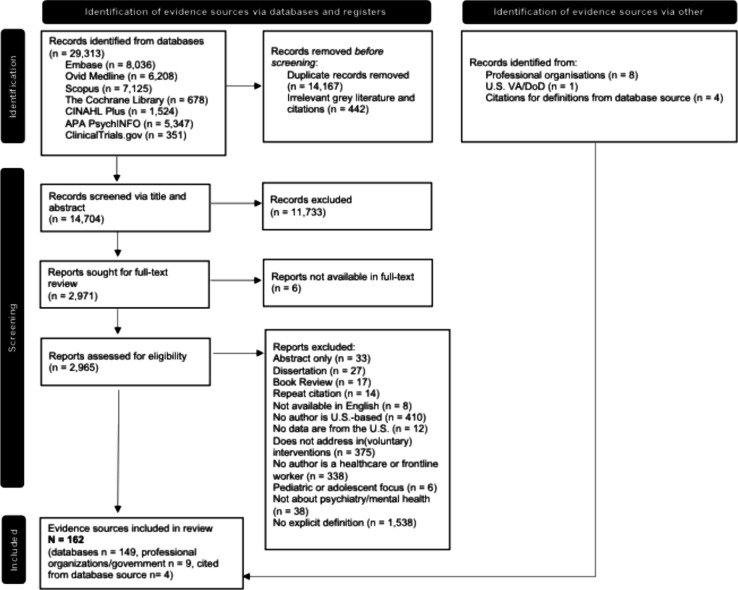
Prisma flow diagram for a scoping review of how U.S. healthcare clinicians define “involuntary” in psychiatric, behavioral, and mental healthcare

The initial database search yielded 29,313 evidence sources. Figure 1, the review’s flow diagram, shows the stepwise screening process which resulted in a final sample of 162 evidence sources. The final citation list and a table containing data about each evidence source are in the supplemental results.

### Characteristics of Evidence Sources

Table 1 shows characteristics of the final 162 evidence sources, of which the most common types were research articles (*n* = 53), review articles (*n* = 39), and book excerpts (*n* = 32). Source years were between 1966 and 2023 (median = 2005). The most common author credential was M.D./D.O. (*n* = 113) followed by Ph.D. (*n* = 82).

**Table 1:** Characteristics of included sources of evidence (*N* = 162)

Characteristic	*n*	%
Year published		
1960–1969	2	1
1970–1979	5	3
1980–1989	17	11
1999–1999	27	17
2000–2009	42	26
2010–2019	53	33
2020–2023	16	10
Evidence type		
Research article	53	33
Review article	39	24
Book excerpt	32	20
Editorial, commentary, or letter	13	8
Educational materials	11	7
Clinical guidelines	5	3
Case report	2	1
Technical report	2	1
Magazine style article	2	1
Dictionary entry	2	1
Position statement	1	1
Author credential^1^		
M.D., D.O., or equivalent	113	70
Ph.D.	82	51
Psychology or counseling	16	10
Nursing	12	7
Social work	12	7

^1^Does not add up to 100% because some source authors had different or multiple credentials

### Number of (In)Voluntary Definitions and the Specific Terms Defined

Among the 162 evidence sources, there were 203 definitions provided (hereafter referred to as the definitions reviewed), with 40 for “voluntary” terms and 163 for “involuntary” terms. There were 126 sources with one definition, 31 sources with two definitions, and 5 sources with three definitions.

Among the definitions reviewed, (in)voluntary was a standalone word for 21 terms and part of a multi-word term for 182 terms. There were a variety of concepts that were described as (in)voluntary multi-word terms, including interventions (hospitalization, mental hospitalization, outpatient commitment, outpatient treatment, civil commitment, commitment, admission, admission for treatment, treatment, mental health treatment, treatment decision, admission to a mental hospital, discharge, medication, inpatient civil commitment, hold, psychiatric hold, mental health holds psychiatric examination, and electroconvulsive therapy [ECT]), people (clients, patients, psychiatric patient, inpatients, and subjects), clinical process concepts (involuntary beds in state hospitals, the involuntary treatment system, and voluntary service agreement), and legal references (legal status, involuntary and voluntary statuses, involuntary treatment laws, substance use laws for involuntary commitment, voluntary participation in mental health court, involuntary outpatient commitment statutes, civil commitment laws, and civil commitment cases).

Table 2 shows definition dimensions (legal, ethical, and safety; see methods section for explanation), key definition words for “involuntary,” and the most frequently defined terms. Illustrative definitions for the most defined terms are found in supplemental results.

**Table 2:** Definition dimensions and definition words for most frequently defined (in)voluntary terms among scoping review included sources (1966–2023)

		Definition dimension^1^			
Selected terms	Number of definitions	Legal	Ethical	Safety	Most frequent (in)voluntary-associated key definition words
	*n (%)*	*%*	*%*	*%*	*Word – number of definitions*
TOTAL	203 (100%)	67%	33%	26%	Order – 25 Coercion – 23 Against a patient’s will/wishes – 23 Consent – 18 Force – 13 Adhere – 12
Involuntary outpatient commitment	35 (17%)	94%	17%	31%	Order – 21 Adhere – 11 Comply – 8 Mandate – 5 Compel – 3 Coercion – 3 Force – 3
Involuntary (psychiatric/mental) hospitalization	21 (10%)	81%	38%	38%	Confine – 5 Against a patient’s will/wishes – 4 Freedom– 2 Liberty – 2 Autonomy vs. beneficence – 2
Involuntary commitment	16 (6%)	81%	31%	31%	Against a patient’s will/wishes – 4 Order – 3 Confine – 2 Deprive liberty – 2 Autonomy vs. beneficence – 2 Force—2
Involuntary treatment	12 (6%)	25%	67%	17%	Consent – 3 Against a patient’s will/wishes – 3 Coercion – 3 Refusal – 3 Over/despite objection – 2 Capacity – 2 Competence – 2
Involuntary *(single word)*	11 (5%)	45%	27%	9%	Against a patient’s will/wishes – 3 Legally/civilly committed – 2
Involuntary civil commitment	9 (4%)	89%	33%	67%	Mandate – 4 Force – 3 Require – 2

^1^Definitions could have more than one dimension or none, therefore these rows do not add up to 100%

The earliest evidence sources identified were two from the 1960s. One defined involuntary hospitalization as “hospitalized without consent,” ^20^^(p. 53)^ and the other defined “involuntary hospitalization, or commitment” as “the legal procedure which confines the mentally ill to an appropriately designated hospital” ^21^^(p. 1267)^.

Among the (in)voluntary terms, involuntary outpatient commitment had the most consistent definition. The most common key words in the definition were: order, adhere, comply, mandate, compel, coercion, force, protested, and reluctant. The least consistent definitions were found for involuntary treatment, whose most frequent key (in)voluntary definitions words were three mentions each of consent, against a patient’s will/wishes, coercion, and refusal.

### (In)Voluntary As a Standalone Word Defined

The term (in)voluntary was defined in isolation or as the focus of the concept in 17 definitions: 11 for involuntary and 6 for voluntary. There were different definitions for (in)voluntary depending on the context. The most common key definition words for involuntary were against the patient’s will/wishes (3 uses) and legally/civilly committed (2 uses).

Examples of involuntary definitions were a decision made by someone other than the patient when describing a “decision to commit” ^22^^(p. 130)^; patients committed against their will when describing “beds in state hospitals” ^23^^(p. 13)^; patients having no choice in some aspect of treatment (e.g., location, treatment site, or psychiatrist), non-compliance resulting in commitment, and could not easily modify treatment plan when discussing “treatment” ^24^^(p. 83)^; against the patient’s will ^25^; and civil commitment ^26^. Voluntary examples definitions include self-referred when discussing “clients,” ^27^^(p. 189)^ and an individual not coerced in the context of a “treatment decision.” ^28^^(p. 105)^.

Four definitions directly contrasted voluntary and involuntary. The difference was said to hinge upon on an individual’s consent with respect to treatments (specifically ECT) ^29^; “of their own accord” versus “against the patient’s will” regarding arrival at the hospital ^30^^(p. 122)^; and initiated by the patient versus another adult for mental hospital admissions within North Carolina statutes ^31^.

### Definition Citations

Among the evidence sources, 48 (30%) cited a reference for the definition. There were no single definition or group of definitions that were highly cited for the definition of (in)voluntary. In other words, many different sources were cited infrequently.

### Multiple (In)Voluntary Terms Within One Evidence Source

Most evidence sources (135/202, 67%) used at least one additional (in)voluntary term without defining it. For example, an article would define “involuntary hospitalization” and then later refer to “involuntary treatment” without defining it.

### Specific Laws or Legal References

Some definitions referred to specific laws, regulations, or legal cases. Involuntary psychiatric examination was used interchangeably with Baker Act examination, which refers to the Florida Mental Health Act of 1971 ^32^. Specific state laws or regulations were used as references to define (in)voluntary admission, ^31,33–35^ informal voluntary admissions, ^36^ involuntary patients, ^37^^(p.200)^ voluntary agreement as part of assisted outpatient treatment, ^38^ involuntary legal status, ^39^ and involuntary commitment for alcoholism ^40^. U.S. Air Force regulation was used to define involuntary treatment ^41^.

### Professional Societies

A total of 9 evidence sources were found from the hand search review of the professional societies, organizations, and the U.S. VA/DoD websites. Two definitions were found from the American Psychological Association’s Dictionary of Psychology. “Voluntary admission” was said to occur at the patient’s request, without coercion, and ends when the patient sees fit unlike involuntary hospitalization ^42^. “Involuntary hospitalization” was defined as “confinement of a person with a serious mental illness to a mental hospital by medical authorization and legal direction (as in involuntary civil commitment)” ^43^.

Four evidence sources were found from the American Psychiatric Association. “Non-emergency involuntary medication” was defined as a “clinical and legal process by which a psychiatric patient is administered medication after they have declined acceptance of prescribed medication” ^44^^(p. 1)^. “Involuntary outpatient commitment” was described in a position statement as “mandated under state involuntary commitment statutes… a judge orders a person with severe mental illness to adhere to an outpatient treatment plan” ^45^^(p. 1)^. A resource document provided three definitions for “involuntary outpatient commitment”: “court-ordered… unlikely to adhere without such a program,” “judge orders… to adhere,” and a list of interchangeable terms ^46^^(p. 5)^.

One source was found from the Academy of Consultation-Liaison Psychiatry, which was a collaborative care guide that included “involuntary psychiatric hold” defined by synonym as “civil commitment” by placing it in parenthesis after the term ^47^^(p. 4)^.

The Veterans Health Administration defined “commitment and involuntary mental health treatment” in a healthcare handbook as “the totality of applicable state laws governing involuntary mental health evaluation and treatment, including time limited holds for evaluation, involuntary outpatient treatment, and forced administration of psychotropic medication” ^48^^(p. 3)^. In a Veterans Affairs mental health nursing orientation guidebook, “voluntary hospitalization” was said to “allow the person to freely choose to discharge from the facility by utilizing the proper process,” while “involuntary hospitalization” was said to “[result] in a period of commitment established in compliance with state legal guidelines” ^49^^(p. 107)^.

Two notable uses of (in)voluntary terms without clear definitions were in the *American Psychiatric Glossary *^50^ and *The Principles of Medical Ethics With Annotations Especially Applicable to Psychiatry* ^51^.

### Specific Definition Words for (in)Voluntary Terms

Across all (in)voluntary terms (*n* = 163), the most frequently used key definition words were order (found in 25 definitions), coercion (23 definitions), and against or opposed to a patient’s will/wishes (23 definitions). The ethical concept of autonomy versus beneficence was used in 6 definitions, and the concepts of capacity and competence were mentioned in 4 and 7 definitions, respectively.

#### Order

Forms of order appeared in 25 definitions, all of which were for involuntary terms. The term with order found most frequently in the definition was “involuntary outpatient commitment” which was defined as “court-ordered” ^46^^(p. 1)^, ^52^^(p. 863)^, ^53^^(p. 36)^, ^54^^(p. 7)^ ordered by a judge [some sources also add: to comply/adhere], ^45,46,55–61^ “ordered to seek,” ^62^^(p. 339)^ and ordered/orders to adhere ^63,64^. Involuntary commitment was said to be “court-ordered,” ^65^^(p. 181)^, ^66^^(p. 65)^, ^67^^(p. 65)^ and involuntary inpatient treatment was defined with “court-ordered to receive” ^68^^(p. 57)^.

#### Coercion

There were 23 definitions in which coercion was included in the definition of (in)voluntary. Of these, 10 definitions presented coercion dichotomously as either present or absent. Four definitions presented coercion in a more nuanced way. One definition of “involuntary legal status” stated that patients were not necessarily coerced, ^39^ while other definitions stated that “involuntary treatment,” “involuntary mental hospitalization,” “involuntary outpatient commitment,” “involuntary outpatient treatment,” and “involuntary civil commitment” were coercive. “Voluntary” terms (“treatment,” “treatment decision,” “clients,” “situated,” and “admission”) were said to be not coerced, uncoerced, no coercion permissible, without coercion, or without improper inducement or coercion. Sources with more specific concepts related to coercion distinguished between “coerced voluntaries” ^69^^(p. 167)^ as patients who were coerced to be a voluntary patient (“sign themselves in as voluntary patients”), “uncoerced involuntaries” to mean those involuntary patients who were “actually seeking hospitalization,” ^69^^(p. 168)^ “coerced involuntary retention” to mean “patients who clearly expressed the wish to avoid hospitalization” ^70^^(p. 516)^.

#### A Patient’s Will/Wishes

There were 23 definitions that explained an involuntary intervention as something that was against/opposed to the will/wishes of a patient; no definitions of voluntary included this language. Among these, as examples, one definition term was “involuntary” alone, ^25^ three were involuntary treatment, ^71–73^ four were hospitalization derivatives, ^30,74–76^ one was patient, ^77^ one was intervention, ^78^ and five were [civil] commitment ^40,79–82^.

#### Consent

Consent was mentioned in 18 definitions, 11 of which were involuntary terms, and seven were voluntary terms. Voluntary treatments ^29^ and medications ^83^ were said to be done with patient consent, and another definition stated that voluntary interventions were done with patient informed consent ^78^. A “voluntary” civil commitment was said to be one in which consent could be withdrawn and lead to discharge ^84^^(p. 321)^. Voluntary inpatients were said to be persons who presented, requested, and consented to treatment of illness ^85^. For involuntary terms, most commonly, involuntary treatments, hospitalization, and confinement were said to be done without patient consent. Involuntary legal status was defined using “without consent” and “non-consenting,” although not necessarily coerced ^39^^(p. 89)^. The involuntary admissions process was defined as one where the patient is “unwilling” to consent to hospitalization ^86^^(p. 872)^. Patients were said to, in many instances, lack the capacity to consent in one definition of involuntary treatment ^87^. The term “involuntary voluntary” was defined as a patient who is labeled as admitted voluntarily but actually is coerced into the decision and therefore repression of true informed consent is concealed ^88^.

#### Autonomy Versus Beneficence

The medical ethics concepts of autonomy and beneficence were listed as part of six explicit definitions, all of which were involuntary interventions. The most common terms were involuntary hospitalization (*n* = 2) and involuntary commitment (*n* = 2). Specific phrasings were that doctors, with the help of the state, deprived patients of autonomy and liberty for the purpose of beneficence ^89^; autonomy and beneficence were values in conflict ^66,67,86^; patient autonomy was precluded for beneficial treatment ^90^; and a temporary override of paternalistic beneficence over personal autonomy occurred ^91^.

#### Competence and Capacity

Forms of competence (*n* = 7, 4 involuntary and 3 voluntary), capacity (*n* = 3, all involuntary), and decision-making capacity (*n* = 1, involuntary) were used in some definitions. While these terms can mean different things in different contexts, that distinction was not always made. An individual was said to be “temporarily incapacitated” as part of one definition for involuntary hospitalization, ^91^^(p. 752)^ while another source defined involuntary civil commitment for “persons with incapacitating mental illness” ^92^^(p. 454)^. For two involuntary treatment definitions, one stated that the patient lacked decision-making capacity, ^93^ and another stated that the patient lacked the capacity to consent or refuse treatment ^93^. For competence, the judicial system was mentioned for some definitions. In one source, an involuntary patient was defined with five possible situations, one of which was “incompetent to stand trial” ^94^^(p. 124)^. One definition of involuntary hospitalization stated that the patient is assumed competent and can refuse treatment unless proven in court ^95^. Participation in mental health courts was said to be voluntary and therefore “defendants must be competent to stand trial” ^96^^(p. 518)^. For non-explicitly judicial uses, involuntary treatment was defined as for patients who were “mentally incompetent” ^41^^(p. 47)^ and “incompetent to make decisions” ^72^^(p. 467)^. A voluntary psychiatric patient was defined to be “virtually by definition competent,” and an involuntary psychiatric patient was “presumed to be competent also, unless evidence exists to demonstrate otherwise. Only in this latter situation would involuntary treatment be possible” ^97^^(p. 92)^.

### Patient Actively Against Versus Patient Without Active Agreement

Definitions of “involuntary” were categorized based on their assessment of patient voluntariness. Overall, *n* = 33 (23%) of evidence sources with involuntary definitions characterized the patient as *actively against* (e.g. “against his or her will,” “despite a patient’s objection,” and “overridden their treatment refusal”), *n* = 16 (11%) said the patient was *without active agreement* (e.g., “without their consent,” and “nonadherent to treatment,” and “unwilling individual”), *n* = 13 (9%) said that involuntary includes *both actively against and without active agreement* (e.g., “almost inherently unwanted” and “patient reluctant or unable to follow through”), and *n* = 80 (56%) did not address patient voluntariness or could not be clearly categorized. The only notable difference among the specific involuntary term was that “involuntary outpatient commitment” was more frequently defined in the category of *both actively against and without active agreement* (27% of sources) compared with the other involuntary terms. The term “nonvoluntary” was only defined once and did not include any information about voluntariness from this perspective in the definition ^98^^(p. 48)^.

**Figure 2:**
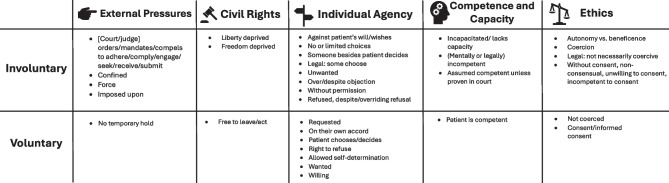
Meaning categories of key definition words of “involuntary” and “voluntary” in psychiatry, behavioral, and mental health in clinician-authored sources, 1966-2023

Figure 2 shows the five meaning categories of the key definition words identified from codes: external pressures, civil rights, individual agency, competence and capacity, and ethics.

## Discussion

This scoping review of how U.S. clinicians explicitly define (in)voluntary concepts in the literature found no definition consensus, a lack of meaning clarity, and frequent use without definition as evidenced by the many sources that were excluded due to lack of an explicit definition. There were a wide range of different (in)voluntary terms and definitions in the 162 included psychiatry and behavioral health publications, which were mostly research articles, review papers, and books authored by persons with an M.D./D.O. and/or Ph.D. credential from the past 6 decades. The variety of definition dimensions—found here to be through legal, ethical, and safety lenses—suggests that consistent meaning for a given term cannot be implied without explanation, and that the compositional meaning of (in)voluntary changes depending on its collocation.

It was striking to find an absence of agreement or any highly cited definitions of (in)voluntary concepts, given that it has been referred to as a core ethical problem in psychiatry ^86,89^. It is notable that over one-half (*n* = 1,538, 52%) of all sources excluded during the full-text screening stage lacked explicit definitions for the (in)voluntary term. Adding to this confusion was the common practice of authors defining a/an (in)voluntary term but then introducing one or more additional (in)voluntary terms without additional definitions. Perhaps authors believed that the meaning was self-evident or was implied by the source context or surrounding text. However, the findings from this review show that terms described as (in)voluntary, and even identical terms, have different definitions in different publications and contexts. This problem suggests that authors should clearly and explicitly define (in)voluntary terms in their text to avoid confusion.

Analysis showed the clinician-authored definitions of “involuntary” most frequently described the patient recipient as *actively against*, and less commonly described the patient as *without active agreement* or *both actively against/without active agreement.* Competing meanings from definitions for the same terms were found. For example, involuntary treatment was defined as both *without patient consent* and *against the patient’s will/wishes*, which are potentially two different clinical scenarios. Altogether, this review provides evidence of term imprecision as it relates to describing voluntariness and supports arguments that utilizing the term nonvoluntary, and perhaps other terms such as “involuntary voluntary” could be one step to increase communication clarity and capture nuance.

There are many real-world clinical scenarios that illustrate the importance of clarifying (in)voluntary term definitions. For example, consider a patient with recurrent catatonia who responds only to ECT and consents to future treatments when decision-making capacity is recovered. Would then future ECT be considered involuntary when the patient is unresponsive and cannot consent again? How is the treatment classified when the decision is made on behalf of a patient by a surrogate decision-maker who knows the patient’s values, wishes, or desires? Psychiatric advance directives, where patients state their future wishes regarding potential involuntary interventions, represent a related critical area for clear understanding. Health disparities are an important part of the conversation, given data showing that interventions that may be considered involuntary occur more commonly among special populations, including those experiencing poverty ^99^ and Black patients and other patients of color ^100^.

Definitions which conflate “involuntary” with “against the patient’s wishes” ignore the administrative reality that admission to some psychiatric hospitals bureaucratically require the use of civil commitment, even if the patient desires treatment. For example, a patient may be mentally ill and suffering with intent and plan to commit suicide, and desires inpatient treatment. Yet the requested hospitalization cannot proceed without civil commitment.

The enormity of use and the variety of the use of the term “involuntary” within scholarship, society guidelines, and other publications reduce the confidence that there will be an organic, grassroots movement towards a universal consensus of definition. This review attempts to stitch together the patchwork of meanings uncovered in the explicit definitions compiled in this review, but to be clear that this should not be mistaken for reaching a consensus on the meaning of (in)voluntary overall or even for the specific terms that contain (in)involuntary as a descriptor. In fact, one single, universal definition for (in)voluntary may be impossible because of the clear incongruence between the lexical definition of involuntary and the referent meaning in specific contexts here.

## Implications for Behavioral Health

Consistent understanding for what involuntary interventions entail within behavioral health services (such as provision of mental health care and/or substance use treatment) is critical, given that it often involves suspension of a person’s fundamental right to autonomy. Yet findings here show there is no consensus in the face of a variety of defined meanings in the literature, which include components of external pressures, civil rights, individual agency, competence and capacity, and ethics. This scoping review provides a description and analysis of the ways the term is defined by U.S. clinicians involved in behavioral health services, mental healthcare, and psychiatry in the extant literature, a step towards reaching clarity of understanding for a problem that has been neglected despite having been pointed out for decades. Finding here, especially Fig. 2, may be useful for behavioral health clinicians seeking to increase precision in their language.

Based on the findings, the authors present several recommendations: (1) clinicians in psychiatry and behavioral health should clearly and explicitly explain with a definition what they mean if deploying an (in)voluntary term in patient charts and discourse (e.g., involuntary in the legal sense of civil commitment, involuntary in the external pressure sense of against the patient’s volition, involuntary in the ethical-procedural sense of without informed consent); (2) professional organizations should recognize the lack of definitional clarity and offer definitions for (in)voluntary and related terms that include the dimensions identified here (legal, ethical, and safety); (3) laws, policies, and regulations should explicitly define any (in)voluntary terms used; (4) clinicians, scholars, and law- and policy-makers should consider using more nuanced terms (e.g., nonvoluntary, which would also requirement agreement on its meaning) and definitions to reflect the ethical and legal differences that exist among clinical situations that are currently described under the same term.

This scoping review had several limitations and opens the door for potential future research. First, implicit definitions, i.e., definitions that could be deduced based on the context in which the term was used, were not included. An analysis of implicit definitions could lead to greater understanding of (in)voluntary’s meaning in the literature. Second, this study was focused on the United States and did not compare how clinicians in other countries or world regions use (in)voluntary. A more detailed historical analysis could uncover greater understanding of how important events, legal decisions, and cultural attitudes impacted how clinicians define (in)voluntary. Given the close relationship of the legal system with (in)voluntary interventions, a review of sources authored solely by legal professionals could provide an additional perspective. Furthermore, (in)voluntary care occurs in all areas of medicine, so a review of broader review of specialties could provide further insight. Finally, only publicly available content was reviewed, so any private, paywall, or members-only content with (in)voluntary explicit definitions were not included.

## Supplementary Information

Below is the link to the electronic supplementary material.

Supplementary file1 (DOCX 251 KB)
