# Supplementary material for: “Involuntary” and “Voluntary” in Psychiatric, Behavioral, and Mental Health Services: A Scoping Review of Definitions
Source: J Behav Health Serv Res. 2025 Mar 26;53(1):142–64. doi: 10.1007/s11414-025-09940-8 (PMC12876088; doi:10.1007/s11414-025-09940-8)
Supplement: Supplementary file 1 — Supplementary file1 (DOCX 251 KB) [file 11414_2025_9940_MOESM1_ESM.docx]

Table of Contents

[1. Inclusion and exclusion criteria, and exclusion hierarchy 2](#_Toc176241837)

[2. Search Strategy 4](#_Toc176241838)

[3. Full citation list of included sources in the scoping review 11](#_Toc176241839)

[4. Appendix. Table of data about included sources including citation, evidence type, and excerpt containing the (in)voluntary definition 23](#_Toc176241840)

[5. Illustrative definitions for (in)voluntary alone and five most common (in)voluntary terms identified in scoping review search 89](#_Toc176241841)

[6. (In)voluntary parts of speech and specific concepts 91](#_Toc176241842)

[7. Repeated definitions 92](#_Toc176241843)

[8. Comparison of key definition words pre-2005 versus post-2005 93](#_Toc176241844)

[9. Term Equivalence for Civil Commitment and Others 94](#_Toc176241845)

[10. Force and Adhere – Key Definition Words 95](#_Toc176241846)

[11. Discussion of MeSH Terms 96](#_Toc176241847)

# 1. Inclusion and exclusion criteria, and exclusion hierarchy

**Table.** (In)voluntary meaning scoping review inclusion and exclusion criteria organized by population, concept, and context framework

|  | Inclusion Criteria | Exclusion Criteria |
| --- | --- | --- |
| Population | - N/A | - No data, cases, or decisions described are from the U.S. - Pediatric or adolescent populations (minors) are the target of involuntary interventions described. |
| Concept | - Written by healthcare professional authors or physician or psychological professional organizations. Healthcare professional author may include psychiatrist or other medical doctors (MD or, DO degree), clinical psychologist (PhD or PsyD degree), counselor/therapist (LPC, LMFT, or LCADAC certification), clinical social worker (MSW degree), nurse practitioner (MSN or DNP degree), or nurse (ADN or BSN degree). Physician or psychological professional organizations are any independent, organized body that claims to represent the aforementioned healthcare professionals. We will include publications by multiple authors where some are not healthcare professionals. - Contain the word “involuntary” or some iteration of the base word “voluntary” in the title, abstract, or text body - Authors are based in the United States. Where there are multiple authors and not all authors are based in the US, at least one author is based in the US. In those cases, inclusion/exclusion decisions will be made on a case-by-case basis depending on whether any of the data, cases, or decisions involved are from the US. - Contain an explicit definition of “involuntary” as it applies to healthcare interventions or decisions involving psychiatry, mental health, or behavioral health (such as hospital admission, detainment, procedures, medications, treatment techniques, outpatient care). | - No author is a healthcare professional or physician or psychological professional organization. Healthcare professional author may include psychiatrist or other medical doctors (MD or, DO degree), clinical psychologist (PhD or PsyD degree), counselor/therapist (LPC, LMFT, or LCADAC certification), clinical social worker (MSW degree), nurse practitioner (MSN or DNP degree), or nurse (RN; ADN or BSN degree). Physician or psychological professional organizations are any independent, organized body that claims to represent the aforementioned healthcare professionals. - Not about the practice of psychiatry or does not concern psychiatric disorders, mental health, or behavioral health - Does not address interventions described as involuntary or the base word voluntary. - Does not contain an explicit definition of “involuntary” as it applies to healthcare interventions or decisions involving psychiatry, mental health, or behavioral health (such as hospital admission, detainment, procedures, medications, treatment techniques, outpatient care). |
| Context | - Written or translated into English - Available to us in full-text - Identified through our search strategy | - Repeat citation - Not written or translated into English - No author is a U.S.-based clinician or organization - Abstract only - Dissertation - Book review - Not available to us in full-text. |

**Exclusion hierarchy:**

1. Not written or translated into English
2. No author is a U.S.-based clinician or organization
3. Abstract only
4. Dissertation
5. Book review
6. Not available to us in full-text.
7. No author is a healthcare professional or physician or psychological professional organization. Healthcare professional author may include psychiatrist or other medical doctors (MD or, DO degree), clinical psychologist (PhD or PsyD degree), counselor/therapist (LPC, LMFT, or LCADAC certification), clinical social worker (MSW degree), nurse practitioner (MSN or DNP degree), or nurse (RN; ADN or BSN degree). Physician or psychological professional organizations are any independent, organized body that claims to represent the aforementioned healthcare professionals.
8. Not about the practice of psychiatry or does not concern psychiatric disorders, mental health, or behavioral health
9. No data, cases, or decisions described are from the U.S.
10. Does not address interventions described as involuntary or the base word voluntary.
11. Does not contain an explicit definition of “involuntary” as it applies to healthcare interventions or decisions involving psychiatry, mental health, or behavioral health (such as hospital admission, detainment, procedures, medications, treatment techniques, outpatient care).
12. Pediatric or adolescent populations (minors) are the target of involuntary interventions described.
13. Repeat citation

# 2. Search Strategy

**Embase**
Date Searched: 7/11/2022

Applied Database Supplied Limits: none

Number of Results: 8,036

Full Search Strategy:

('involuntary commitment'/exp OR 'commitment'/exp OR involuntary*:ti,ab,kw OR ((civil* OR coercive* OR compulsory* OR legal* OR nonvoluntar* OR 'non-voluntar*' OR 'non voluntary*') NEAR/4 (commitment* OR committed OR treatment* OR hospitalization* OR admission* OR administration OR therap*)):ti,ab,kw) AND ('addiction'/exp OR 'anorexia nervosa'/exp OR 'bulimia'/exp OR 'binge eating disorder'/exp OR 'behavior disorder'/exp OR 'disruptive behavior'/exp OR 'drug seeking behavior'/exp OR 'impulse control disorder'/exp OR 'psychosocial disorder'/exp OR 'suicidal behavior'/exp OR 'behavioral health'/exp OR 'behavioral health care'/exp OR 'drug dependence'/exp OR 'eating disorder'/exp OR 'forensic psychiatry'/exp OR 'mental health'/exp OR 'mental disease'/exp OR 'opiate addiction'/exp OR 'narcotic dependence'/exp OR 'psychiatry'/exp OR 'psychosis'/exp OR 'severe mental illness'/exp OR 'substance use'/exp OR (addiction OR addictive OR 'chemical dependenc*' OR anorexia OR bulimia OR diabulimia OR hyperrexia):ti,ab,kw OR ((behavior* OR behaviour*) NEAR/3 (health OR disorder* OR aberrant OR aberration OR disturb* OR crisis OR crises OR deviant OR disruptive OR 'drug seeking' OR 'drug-seeking' OR suicidal OR psychosocial)):ti,ab,kw OR ((reactive OR impulse OR psychosocial OR unipolar) NEAR/3 disorder*):ti,ab,kw OR (psychosocial NEAR/2 (disease* OR problem*)):ti,ab,kw OR ('neurobehavioral manifestation*' OR suicidality OR depress*):ti,ab,kw OR ((drug OR substance OR 'substance-related') NEAR/3 (dependen* OR addict* OR facilitation OR habituation OR disorder*)):ti,ab,kw OR (addict OR toxicomani*):ti,ab,kw OR ((eating OR feeding) NEAR/3 (disorder* OR behavior* OR behavior* OR patholog*)):ti,ab,kw OR ('forensic psychiat*' OR 'insanity defense'):ti,ab,kw OR (commitment NEAR/1 'mentally ill'):ti,ab,kw OR ((mental* OR psychic) NEAR/3 (health OR care OR condition* OR factor* OR help OR services* OR state OR status OR ill* OR disease* OR abnormal* OR confusion OR defect* OR disorder* OR disturbance* OR insufficienc*)):ti,ab,kw OR (insanity OR psychopathology OR narcotism):ti,ab,kw OR ((neurodevelopmental OR neuropsychiatric OR psychiatric OR psychic OR psychological) NEAR/3 (disorder* OR disease* OR disturbance* OR illness* OR symptom*)):ti,ab,kw OR ((narcotic* OR opiate OR opioid* OR 'opioid-related' OR opium OR 'opioid-related') NEAR/3 (dependenc* OR abuse OR depression OR disorder*)):ti,ab,kw OR (psychiatry OR orthopsychiatry OR psychiatric* OR psychosis OR psychoses OR encephalopschychosis OR psychotic*):ti,ab,kw) AND ('psychosocial intervention'/exp OR 'hospitalization'/exp OR 'commit'/exp OR 'treatment outcome'/exp OR 'outpatient'/exp OR 'electroconvulsive therapy'/exp OR 'convulsive therapy'/exp OR 'hospital admission'/exp OR 'benzodiazepine derivative'/exp OR 'benzodiazepine'/exp OR 'diazepam'/exp OR 'haloperidol'/exp OR 'injection'/exp OR 'drug therapy'/exp OR 'oral drug administration'/exp OR 'drug administration'/exp OR 'administration of drugs, food and chemicals'/exp OR 'intravenous drug administration'/exp OR 'neuroleptic agent'/exp OR 'physical restraint'/exp OR 'restraining device'/exp OR 'seclusion'/exp OR (intervention* OR hospitalization OR committed OR ‘civil commitment’ OR outpatient OR ‘out patient*’ OR ‘out-patient*’):ti,ab,kw OR ((treatment OR ‘health care’ OR healthcare OR ‘health-care’ OR management OR patient OR therap*) NEAR/3 outcome*):ti,ab,kw OR (‘electroconvulsive therap*’ OR ‘electric convulsive’ OR electroconvulsant OR electroshock OR convulsive):ti,ab,kw OR (benzodiazepine* OR benzodiazepinone*):ti,ab,kw OR (hospital NEAR/1 (admission* OR admit*)):ti,ab,kw OR (diazepam OR alboral OR aliseum OR alupram OR amiprol OR ansiolin OR antenex OR anxionil OR apaurin OR apaurine OR ‘apo-diazepam’ OR apozepam OR arzepam OR assival OR atensine OR audium OR azedipamin OR benzopin OR betapam OR bialzepam OR bialzepan OR calmpose OR caudel OR cercin OR cercine OR cersine OR chlordiazepam OR compaz OR desconet OR diaceplex OR dialag OR dialar OR diano OR diapam OR diapanil OR diapax OR diapin OR diapine OR diapo OR diaquel OR diastat OR diazelium OR diazemuls OR diazepan OR diazepen OR diazepin OR diazidem OR dipaz OR dipezona OR dizac OR doval OR drenian OR ducene OR dupin OR duxen OR eridan OR ‘euphorin p’ OR eurosan OR evacalm OR fanstan OR faustan OR gewacalm OR gubex OR kratium OR lamra OR lembrol OR libervant OR lipodiazepam OR lorinon OR lovium OR melode OR mentalium OR methyldiazepinon OR methyldiazepinone OR morosan OR neocalme OR neurolytril OR nivalen OR noan OR novazam OR ortopsique OR paceum OR pacitran OR paxum OR plidan OR propam OR psychopax OR ‘q-pam’ OR radizepam OR relanium OR reliver OR reposepan OR saromet OR sedapam OR seduxen OR serendin OR setonil OR simasedan OR sipam OR sonacon OR stesolid OR stesolin OR ‘tanquo tablinen’ OR tranimul OR tranquirit OR ‘tranquo puren’ OR trazepam OR umbrium OR valaxona OR valiquid OR valium OR valpam OR valrelease OR valtoco OR vanconin OR vatran OR vazen OR vival OR vivol OR zetran):ti,ab,kw OR ((drug OR medicine* OR medication* OR medicament OR pharmacological OR intravenous OR ‘IV’ OR vein OR venous) NEAR/3 (administration OR inject* OR ‘re-administration’ OR readministration OR infusion* OR transfusion)):ti,ab,kw OR (haloperidol OR alased OR aloperidin OR aloperidine OR ‘apo-haloperidol’ OR benison OR brotopon OR celenase OR cereen OR cerenace OR cizoren OR depidol OR dores OR dozic OR duraperidol OR fortunan OR govotil OR Haldol OR halidol OR ‘halo-p’ OR halojust OR halomed OR haloneural OR haloper OR ‘haloperidol hydrochoride’ OR ‘haloperidol intensol’ OR ‘haloperidol lactate’ OR haloperil OR haloperin OR haloperitol OR halopidol OR halopol OR halosten OR haricon OR keselan OR linton OR mixidol OR novoperidol OR peluces OR perida OR peridol OR peridor OR selezyme OR seranace OR serenace OR serenase OR serenelfi OR siegoperidol OR sigaperidol OR ‘trancodol-10’ OR ‘trancodol-5’):ti,ab,kw OR (neuroleptic OR antipsychotic* OR ‘butyrophenone tranquilizer*’ OR ‘major tranquilizer*’ OR ‘major tranquillizer*’ OR neurolepticum OR ‘phenothiazine tranquilizer*’):ti,ab,kw OR ((physical OR mechanical) NEAR/3 restraint*):ti,ab,kw OR (‘restraining devic*’ OR seclusion OR isolation OR ‘therapeutic hold*’):ti,ab,kw)

**Ovid Medline**
Date Searched: 7/11/2022
Applied Database Supplied Limits: none
Number of Results: 6,208

Full Search Strategy:

(exp "Commitment of Mentally Ill"/ OR exp Involuntary Commitment/ OR exp Involuntary Treatment/ OR involuntary*.ti,ab,kf. OR (((civil* OR coercive* OR compulsory* OR legal* OR nonvoluntar* OR non-voluntar* OR non voluntary*) ADJ4 (commitment* OR committed OR treatment* OR hospitalization* OR admission* OR administration OR therap*)).ti,ab,kf.)) AND (exp Substance-Related Disorders/ OR exp Anorexia Nervosa/ OR exp Bulimia Nervosa/ OR exp Bulimia/ OR exp Binge-Eating Disorder/ OR exp Mental Disorders/ OR exp Problem Behavior/ OR exp Drug-Seeking Behavior/ OR exp "Disruptive, Impulse Control, and Conduct Disorders"/ OR exp Suicide, Attempted/ OR exp Suicidal Ideation/ OR exp "Feeding and Eating Disorders"/ OR exp Forensic Psychiatry/ OR exp Mental Health/ OR exp Opioid-Related Disorders/ OR exp Narcotic-Related Disorders/ OR exp Psychiatry/ OR exp Psychotic Disorders/ OR (addiction OR addictive OR chemical dependenc* OR anorexia OR bulimia OR diabulimia OR hyperrexia).ti,ab,kf. OR ((behavior* OR behaviour*) ADJ3 (health OR disorder* OR aberrant OR aberration OR disturb* OR crisis OR crises OR deviant OR disruptive OR drug seeking OR drug-seeking OR suicidal OR psychosocial)).ti,ab,kf. OR ((reactive OR impulse OR psychosocial OR unipolar) ADJ3 disorder*).ti,ab,kf. OR (psychosocial ADJ2 (disease* OR problem*)).ti,ab,kf. OR (neurobehavioral manifestation* OR suicidality OR depress*).ti,ab,kf. OR ((drug OR substance OR substance-related) ADJ3 (dependen* OR addict* OR facilitation OR habituation OR disorder*)).ti,ab,kf. OR (addict OR toxicomani*).ti,ab,kf. OR ((eating OR feeding) ADJ3 (disorder* OR behavior* OR behavior* OR patholog*)).ti,ab,kf. OR (forensic psychiat* OR insanity defense).ti,ab,kf. OR (commitment ADJ1 mentally ill).ti,ab,kf. OR ((mental* OR psychic) ADJ3 (health OR care OR condition* OR factor* OR help OR services* OR state OR status OR ill* OR disease* OR abnormal* OR confusion OR defect* OR disorder* OR disturbance* OR insufficienc*)).ti,ab,kf. OR (insanity OR psychopathology OR narcotism).ti,ab,kf. OR ((neurodevelopmental OR neuropsychiatric OR psychiatric OR psychic OR psychological) ADJ3 (disorder* OR disease* OR disturbance* OR illness* OR symptom*)).ti,ab,kf. OR ((narcotic* OR opiate OR opioid* OR opioid-related OR opium OR opioid-related) ADJ3 (dependenc* OR abuse OR depression OR disorder*)).ti,ab,kf. OR (psychiatry OR orthopsychiatry OR psychiatric* OR psychosis OR psychoses OR encephalopschychosis OR psychotic*).ti,ab,kf.) AND (exp Psychosocial Intervention/ OR exp Hospitalization/ OR exp Treatment Outcome/ OR exp Outpatients/ OR exp Electroconvulsive Therapy/ OR exp Convulsive Therapy/ OR exp Benzodiazepines/ OR exp Diazepam/ OR exp Haloperidol/ OR exp Injections/ OR exp Drug Therapy/ OR exp Administration, Oral/ OR exp Drug Administration Routes/ OR exp Administration, Intravenous/ OR exp Antipsychotic Agents/ OR exp Restraint, Physical/ OR (intervention* OR hospitalization OR committed OR civil commitment OR outpatient OR out patient* OR out-patient*).ti,ab,kf. OR ((treatment OR health care OR healthcare OR health-care OR management OR patient OR therap*) ADJ3 outcome*).ti,ab,kf. OR (electroconvulsive therap* OR electric convulsive OR electroconvulsant OR electroshock OR convulsive).ti,ab,kf. OR (benzodiazepine* OR benzodiazepinone*).ti,ab,kf. OR (hospital ADJ1 (admission* OR admit*)).ti,ab,kf. OR (diazepam OR alboral OR aliseum OR alupram OR amiprol OR ansiolin OR antenex OR anxionil OR apaurin OR apaurine OR apo-diazepam OR apozepam OR arzepam OR assival OR atensine OR audium OR azedipamin OR benzopin OR betapam OR bialzepam OR bialzepan OR calmpose OR caudel OR cercin OR cercine OR cersine OR chlordiazepam OR compaz OR desconet OR diaceplex OR dialag OR dialar OR diano OR diapam OR diapanil OR diapax OR diapin OR diapine OR diapo OR diaquel OR diastat OR diazelium OR diazemuls OR diazepan OR diazepen OR diazepin OR diazidem OR dipaz OR dipezona OR dizac OR doval OR drenian OR ducene OR dupin OR duxen OR eridan OR euphorin p OR eurosan OR evacalm OR fanstan OR faustan OR gewacalm OR gubex OR kratium OR lamra OR lembrol OR libervant OR lipodiazepam OR lorinon OR lovium OR melode OR mentalium OR methyldiazepinon OR methyldiazepinone OR morosan OR neocalme OR neurolytril OR nivalen OR noan OR novazam OR ortopsique OR paceum OR pacitran OR paxum OR plidan OR propam OR psychopax OR q-pam OR radizepam OR relanium OR reliver OR reposepan OR saromet OR sedapam OR seduxen OR serendin OR setonil OR simasedan OR sipam OR sonacon OR stesolid OR stesolin OR tanquo tablinen OR tranimul OR tranquirit OR tranquo puren OR trazepam OR umbrium OR valaxona OR valiquid OR valium OR valpam OR valrelease OR valtoco OR vanconin OR vatran OR vazen OR vival OR vivol OR zetran).ti,ab,kf. OR ((drug OR medicine* OR medication* OR medicament OR pharmacological OR intravenous OR IV OR vein OR venous) ADJ3 (administration OR inject* OR re-administration OR readministration OR infusion* OR transfusion)).ti,ab,kf. OR (haloperidol OR alased OR aloperidin OR aloperidine OR apo-haloperidol OR benison OR brotopon OR celenase OR cereen OR cerenace OR cizoren OR depidol OR dores OR dozic OR duraperidol OR fortunan OR govotil OR Haldol OR halidol OR halo-p OR halojust OR halomed OR haloneural OR haloper OR haloperidol hydrochoride OR haloperidol intensol OR haloperidol lactate OR haloperil OR haloperin OR haloperitol OR halopidol OR halopol OR halosten OR haricon OR keselan OR linton OR mixidol OR novoperidol OR peluces OR perida OR peridol OR peridor OR selezyme OR seranace OR serenace OR serenase OR serenelfi OR siegoperidol OR sigaperidol OR trancodol-10 OR trancodol-5).ti,ab,kf. OR (neuroleptic OR antipsychotic* OR butyrophenone tranquilizer* OR major tranquilizer* OR major tranquillizer* OR neurolepticum OR phenothiazine tranquilizer*).ti,ab,kf. OR ((physical OR mechanical) ADJ3 restraint*).ti,ab,kf. OR (restraining devic* OR seclusion OR isolation OR therapeutic hold*).ti,ab,kf.)

**Scopus**
Date Searched: 7/11/2022
Applied Database Supplied Limits: none
Number of Results: 7,125

Full Search Strategy:

((TITLE-ABS-KEY(involuntary*)) OR (TITLE-ABS-KEY((civil* OR coercive* OR compulsory* OR legal* OR nonvoluntar* OR “non-voluntar*” OR “non voluntary*”) W/4 (commitment* OR committed OR treatment* OR hospitalization* OR admission* OR administration OR therap*)))) AND ((TITLE-ABS-KEY(addiction OR addictive OR “chemical dependenc*” OR anorexia OR bulimia OR diabulimia OR hyperrexia)) OR (TITLE-ABS-KEY((behavior* OR behaviour*) W/3 (health OR disorder* OR aberrant OR aberration OR disturb* OR crisis OR crises OR deviant OR disruptive OR “drug seeking” OR “drug-seeking” OR suicidal OR psychosocial))) OR (TITLE-ABS-KEY((reactive OR impulse OR psychosocial OR unipolar) W/3 disorder*)) OR (TITLE-ABS-KEY(psychosocial W/2 (disease* OR problem*))) OR (TITLE-ABS-KEY(“neurobehavioral manifestation*” OR suicidality OR depress*)) OR (TITLE-ABS-KEY((drug OR substance OR “substance-related”) W/3 (dependen* OR addict* OR facilitation OR habituation OR disorder*))) OR (TITLE-ABS-KEY(addict OR toxicomani*)) OR (TITLE-ABS-KEY((eating OR feeding) W/3 (disorder* OR behavior* OR behavior* OR patholog*))) OR (TITLE-ABS-KEY(“forensic psychiat*” OR “insanity defense”)) OR (TITLE-ABS-KEY(commitment W/1 “mentally ill”)) OR (TITLE-ABS-KEY((mental* OR psychic) W/3 (health OR care OR condition* OR factor* OR help OR services* OR state OR status OR ill* OR disease* OR abnormal* OR confusion OR defect* OR disorder* OR disturbance* OR insufficienc*))) OR (TITLE-ABS-KEY(insanity OR psychopathology OR narcotism)) OR (TITLE-ABS-KEY((neurodevelopmental OR neuropsychiatric OR psychiatric OR psychic OR psychological) W/3 (disorder* OR disease* OR disturbance* OR illness* OR symptom*))) OR (TITLE-ABS-KEY((narcotic* OR opiate OR opioid* OR “opioid-related” OR opium OR “opioid-related”) W/3 (dependenc* OR abuse OR depression OR disorder*))) OR (TITLE-ABS-KEY(psychiatry OR orthopsychiatry OR psychiatric* OR psychosis OR psychoses OR encephalopschychosis OR psychotic*))) AND ((TITLE-ABS-KEY(intervention* OR hospitalization OR committed OR “civil commitment” OR outpatient OR “out patient*” OR “out-patient*”)) OR (TITLE-ABS-KEY((treatment OR “health care” OR healthcare OR “health-care” OR management OR patient OR therap*) W/3 outcome*)) OR (TITLE-ABS-KEY(“electroconvulsive therap*” OR “electric convulsive” OR electroconvulsant OR electroshock OR convulsive)) OR (TITLE-ABS-KEY(benzodiazepine* OR benzodiazepinone*)) OR (TITLE-ABS-KEY(hospital W/1 (admission* OR admit*))) OR (TITLE-ABS-KEY(diazepam OR alboral OR aliseum OR alupram OR amiprol OR ansiolin OR antenex OR anxionil OR apaurin OR apaurine OR “apo-diazepam” OR apozepam OR arzepam OR assival OR atensine OR audium OR azedipamin OR benzopin OR betapam OR bialzepam OR bialzepan OR calmpose OR caudel OR cercin OR cercine OR cersine OR chlordiazepam OR compaz OR desconet OR diaceplex OR dialag OR dialar OR diano OR diapam OR diapanil OR diapax OR diapin OR diapine OR diapo OR diaquel OR diastat OR diazelium OR diazemuls OR diazepan OR diazepen OR diazepin OR diazidem OR dipaz OR dipezona OR dizac OR doval OR drenian OR ducene OR dupin OR duxen OR eridan OR “euphorin p” OR eurosan OR evacalm OR fanstan OR faustan OR gewacalm OR gubex OR kratium OR lamra OR lembrol OR libervant OR lipodiazepam OR lorinon OR lovium OR melode OR mentalium OR methyldiazepinon OR methyldiazepinone OR morosan OR neocalme OR neurolytril OR nivalen OR noan OR novazam OR ortopsique OR paceum OR pacitran OR paxum OR plidan OR propam OR psychopax OR “q-pam” OR radizepam OR relanium OR reliver OR reposepan OR saromet OR sedapam OR seduxen OR serendin OR setonil OR simasedan OR sipam OR sonacon OR stesolid OR stesolin OR “tanquo tablinen” OR tranimul OR tranquirit OR “tranquo puren” OR trazepam OR umbrium OR valaxona OR valiquid OR valium OR valpam OR valrelease OR valtoco OR vanconin OR vatran OR vazen OR vival OR vivol OR zetran)) OR (TITLE-ABS-KEY((drug OR medicine* OR medication* OR medicament OR pharmacological OR intravenous OR “IV” OR vein OR venous) W/3 (administration OR inject* OR “re-administration” OR readministration OR infusion* OR transfusion))) OR (TITLE-ABS-KEY(haloperidol OR alased OR aloperidin OR aloperidine OR “apo-haloperidol” OR benison OR brotopon OR celenase OR cereen OR cerenace OR cizoren OR depidol OR dores OR dozic OR duraperidol OR fortunan OR govotil OR Haldol OR halidol OR “halo-p” OR halojust OR halomed OR haloneural OR haloper OR “haloperidol hydrochoride” OR “haloperidol intensol” OR “haloperidol lactate” OR haloperil OR haloperin OR haloperitol OR halopidol OR halopol OR halosten OR haricon OR keselan OR linton OR mixidol OR novoperidol OR peluces OR perida OR peridol OR peridor OR selezyme OR seranace OR serenace OR serenase OR serenelfi OR siegoperidol OR sigaperidol OR “trancodol-10” OR “trancodol-5”)) OR (TITLE-ABS-KEY(neuroleptic OR antipsychotic* OR “butyrophenone tranquilizer*” OR “major tranquilizer*” OR “major tranquillizer*” OR neurolepticum OR “phenothiazine tranquilizer*”)) OR (TITLE-ABS-KEY((physical OR mechanical) W/3 restraint*)) OR (TITLE-ABS-KEY(“restraining devic*” OR seclusion OR isolation OR “therapeutic hold*”)))

**The Cochrane Library**

Date Searched: 7/11/2022
Applied Database Supplied Limits: none
Number of Results

CENTRAL: 678

CDSR: 44

Full Search Strategy:

([mh “Commitment of Mentally Ill”] OR [mh “Involuntary Commitment”] OR [mh “Involuntary Treatment”] OR involuntary*:ti,ab,kw OR ((civil* OR coercive* OR compulsory* OR legal* OR nonvoluntar* OR “non-voluntar*” OR “non voluntary*”) NEAR/4 (commitment* OR committed OR treatment* OR hospitalization* OR admission* OR administration OR therap*)):ti,ab,kw) AND ([mh “Substance-Related Disorders”] OR [mh “Anorexia Nervosa”] OR [mh “Bulimia Nervosa”] OR [mh “Bulimia”] OR [mh “Binge-Eating Disorder”] OR [mh “Mental Disorders”] OR [mh “Problem Behavior”] OR [mh “Drug-Seeking Behavior”] OR [mh “Disruptive, Impulse Control, and Conduct Disorders”] OR [mh “Suicide, Attempted”] OR [mh “Suicidal Ideation”] OR [mh “Feeding and Eating Disorders”] OR [mh “Forensic Psychiatry”] OR [mh “Mental Health”] OR [mh “Opioid-Related Disorders”] OR [mh “Narcotic-Related Disorders”] OR [mh “Psychiatry”] OR [mh “Psychotic Disorders”] OR (addiction OR addictive OR “chemical dependenc*” OR anorexia OR bulimia OR diabulimia OR hyperrexia):ti,ab,kw OR ((behavior* OR behaviour*) NEAR/3 (health OR disorder* OR aberrant OR aberration OR disturb* OR crisis OR crises OR deviant OR disruptive OR “drug seeking” OR “drug seeking” OR suicidal OR psychosocial)):ti,ab,kw OR ((reactive OR impulse OR psychosocial OR unipolar) NEAR/3 disorder*):ti,ab,kw OR (psychosocial NEAR/2 (disease* OR problem*)):ti,ab,kw OR (“neurobehavioral manifestation*” OR suicidality OR depress*):ti,ab,kw OR ((drug OR substance OR “substance related”) NEAR/3 (dependen* OR addict* OR facilitation OR habituation OR disorder*)):ti,ab,kw OR (addict OR toxicomani*):ti,ab,kw OR ((eating OR feeding) NEAR/3 (disorder* OR behavior* OR behavior* OR patholog*)):ti,ab,kw OR (“forensic psychiat*” OR “insanity defense”):ti,ab,kw OR (commitment NEAR/1 “mentally ill”):ti,ab,kw OR ((mental* OR psychic) NEAR/3 (health OR care OR condition* OR factor* OR help OR services* OR state OR status OR ill* OR disease* OR abnormal* OR confusion OR defect* OR disorder* OR disturbance* OR insufficienc*)):ti,ab,kw OR (insanity OR psychopathology OR narcotism):ti,ab,kw OR ((neurodevelopmental OR neuropsychiatric OR psychiatric OR psychic OR psychological) NEAR/3 (disorder* OR disease* OR disturbance* OR illness* OR symptom*)):ti,ab,kw OR ((narcotic* OR opiate OR opioid* OR “opioid related” OR opium OR “opioid related”) NEAR/3 (dependenc* OR abuse OR depression OR disorder*)):ti,ab,kw OR (psychiatry OR orthopsychiatry OR psychiatric* OR psychosis OR psychoses OR encephalopschychosis OR psychotic*):ti,ab,kw) AND ([mh “Psychosocial Intervention”] OR [mh “Hospitalization”] OR [mh “Treatment Outcome”] OR [mh “Outpatients”] OR [mh “Electroconvulsive Therapy”] OR [mh “Convulsive Therapy”] OR [mh “Benzodiazepines”] OR [mh “Diazepam”] OR [mh “Haloperidol”] OR [mh “Injections”] OR [mh “Drug Therapy”] OR [mh “Administration, Oral”] OR [mh “Drug Administration Routes”] OR [mh “Administration, Intravenous”] OR [mh “Antipsychotic Agents”] OR [mh “Restraint, Physical”] OR (intervention* OR hospitalization OR committed OR “civil commitment” OR outpatient OR “out patient*” OR “out patient*”):ti,ab,kw OR ((treatment OR “health care” OR healthcare OR “health care” OR management OR patient OR therap*) NEAR/3 outcome*):ti,ab,kw OR (“electroconvulsive therap*” OR “electric convulsive” OR electroconvulsant OR electroshock OR convulsive):ti,ab,kw OR (benzodiazepine* OR benzodiazepinone*):ti,ab,kw OR (hospital NEAR/1 (admission* OR admit*)):ti,ab,kw OR (diazepam OR alboral OR aliseum OR alupram OR amiprol OR ansiolin OR antenex OR anxionil OR apaurin OR apaurine OR “apo diazepam” OR apozepam OR arzepam OR assival OR atensine OR audium OR azedipamin OR benzopin OR betapam OR bialzepam OR bialzepan OR calmpose OR caudel OR cercin OR cercine OR cersine OR chlordiazepam OR compaz OR desconet OR diaceplex OR dialag OR dialar OR diano OR diapam OR diapanil OR diapax OR diapin OR diapine OR diapo OR diaquel OR diastat OR diazelium OR diazemuls OR diazepan OR diazepen OR diazepin OR diazidem OR dipaz OR dipezona OR dizac OR doval OR drenian OR ducene OR dupin OR duxen OR eridan OR “euphorin p” OR eurosan OR evacalm OR fanstan OR faustan OR gewacalm OR gubex OR kratium OR lamra OR lembrol OR libervant OR lipodiazepam OR lorinon OR lovium OR melode OR mentalium OR methyldiazepinon OR methyldiazepinone OR morosan OR neocalme OR neurolytril OR nivalen OR noan OR novazam OR ortopsique OR paceum OR pacitran OR paxum OR plidan OR propam OR psychopax OR “q pam” OR radizepam OR relanium OR reliver OR reposepan OR saromet OR sedapam OR seduxen OR serendin OR setonil OR simasedan OR sipam OR sonacon OR stesolid OR stesolin OR “tanquo tablinen” OR tranimul OR tranquirit OR “tranquo puren” OR trazepam OR umbrium OR valaxona OR valiquid OR valium OR valpam OR valrelease OR valtoco OR vanconin OR vatran OR vazen OR vival OR vivol OR zetran):ti,ab,kw OR ((drug OR medicine* OR medication* OR medicament OR pharmacological OR intravenous OR “IV” OR vein OR venous) NEAR/3 (administration OR inject* OR “re administration” OR readministration OR infusion* OR transfusion)):ti,ab,kw OR (haloperidol OR alased OR aloperidin OR aloperidine OR “apo haloperidol” OR benison OR brotopon OR celenase OR cereen OR cerenace OR cizoren OR depidol OR dores OR dozic OR duraperidol OR fortunan OR govotil OR Haldol OR halidol OR “halo p” OR halojust OR halomed OR haloneural OR haloper OR “haloperidol hydrochoride” OR “haloperidol intensol” OR “haloperidol lactate” OR haloperil OR haloperin OR haloperitol OR halopidol OR halopol OR halosten OR haricon OR keselan OR linton OR mixidol OR novoperidol OR peluces OR perida OR peridol OR peridor OR selezyme OR seranace OR serenace OR serenase OR serenelfi OR siegoperidol OR sigaperidol OR “trancodol 10” OR “trancodol 5”):ti,ab,kw OR (neuroleptic OR antipsychotic* OR “butyrophenone tranquilizer*” OR “major tranquilizer*” OR “major tranquillizer*” OR neurolepticum OR “phenothiazine tranquilizer*”):ti,ab,kw OR ((physical OR mechanical) NEAR/3 restraint*):ti,ab,kw OR (“restraining devic*” OR seclusion OR isolation OR “therapeutic hold*”):ti,ab,kw)

**CINAHL Plus**
Date Searched: 7/11/2022
Applied Database Supplied Limits: none
Number of Results: 1,524

Full Search Strategy:

1. (MH "Involuntary Commitment") OR (MH "Involuntary Treatment") OR TI “involuntary*” OR TI ((civil* OR coercive* OR compulsory* OR legal* OR nonvoluntar* OR “non-voluntar*” OR “non voluntary*”) N4 (commitment* OR committed OR treatment* OR hospitalization* OR admission* OR administration OR therap*)) OR AB “involuntary*” OR AB ((civil* OR coercive* OR compulsory* OR legal* OR nonvoluntar* OR “non-voluntar*” OR “non voluntary*”) N4 (commitment* OR committed OR treatment* OR hospitalization* OR admission* OR administration OR therap*))

AND

2. (MH "Substance Use Disorders") OR (MH "Anorexia Nervosa") OR (MH "Anorexia") OR (MH "Bulimia Nervosa") OR (MH "Bulimia") OR (MH "Binge Eating Disorder") OR (MH "Eating Disorders") OR (MH "Feeding and Eating Disorders of Childhood") OR (MH "Mental Disorders") OR (MH "Drug-Seeking Behavior") OR (MH "Impulse Control Disorders") OR (MH "Social Behavior Disorders") OR (MH "Suicide, Attempted") OR (MH "Suicidal Ideation") OR (MH "Forensic Psychiatry") OR (MH "Mental Health") OR (MH "Psychiatry") OR (MH "Psychotic Disorders") OR TI (addiction OR addictive OR “chemical dependenc*” OR anorexia OR bulimia OR diabulimia OR hyperrexia) OR TI ((behavior* OR behaviour*) N3 (health OR disorder* OR aberrant OR aberration OR disturb* OR crisis OR crises OR deviant OR disruptive OR “drug seeking” OR “drug-seeking” OR suicidal OR psychosocial)) OR TI ((reactive OR impulse OR psychosocial OR unipolar) N3 disorder*) OR (psychosocial N2 (disease* OR problem*)) OR TI (“neurobehavioral manifestation*” OR suicidality OR depress*) OR ((drug OR substance OR “substance-related”) N3 (dependen* OR addict* OR facilitation OR habituation OR disorder*)) OR TI (addict OR toxicomani*) OR TI ((eating OR feeding) N3 (disorder* OR behavior* OR behavior* OR patholog*)) OR TI (“forensic psychiat*” OR “insanity defense”) OR TI (commitment N1 “mentally ill”) OR TI ((mental* OR psychic) N3 (health OR care OR condition* OR factor* OR help OR services* OR state OR status OR ill* OR disease* OR abnormal* OR confusion OR defect* OR disorder* OR disturbance* OR insufficienc*)) OR TI (insanity OR psychopathology OR narcotism) OR TI ((neurodevelopmental OR neuropsychiatric OR psychiatric OR psychic OR psychological) N3 (disorder* OR disease* OR disturbance* OR illness* OR symptom*)) OR TI ((narcotic* OR opiate OR opioid* OR “opioid-related” OR opium OR “opioid-related”) N3 (dependenc* OR abuse OR depression OR disorder*)) OR TI (psychiatry OR orthopsychiatry OR psychiatric* OR psychosis OR psychoses OR encephalopschychosis OR psychotic*) OR AB (addiction OR addictive OR “chemical dependenc*” OR anorexia OR bulimia OR diabulimia OR hyperrexia) OR AB ((behavior* OR behaviour*) N3 (health OR disorder* OR aberrant OR aberration OR disturb* OR crisis OR crises OR deviant OR disruptive OR “drug seeking” OR “drug-seeking” OR suicidal OR psychosocial)) OR AB ((reactive OR impulse OR psychosocial OR unipolar) N3 disorder*) OR (psychosocial N2 (disease* OR problem*)) OR AB (“neurobehavioral manifestation*” OR suicidality OR depress*) OR ((drug OR substance OR “substance-related”) N3 (dependen* OR addict* OR facilitation OR habituation OR disorder*)) OR AB (addict OR toxicomani*) OR AB ((eating OR feeding) N3 (disorder* OR behavior* OR behavior* OR patholog*)) OR AB (“forensic psychiat*” OR “insanity defense”) OR AB (commitment N1 “mentally ill”) OR AB ((mental* OR psychic) N3 (health OR care OR condition* OR factor* OR help OR services* OR state OR status OR ill* OR disease* OR abnormal* OR confusion OR defect* OR disorder* OR disturbance* OR insufficienc*)) OR AB (insanity OR psychopathology OR narcotism) OR AB ((neurodevelopmental OR neuropsychiatric OR psychiatric OR psychic OR psychological) N3 (disorder* OR disease* OR disturbance* OR illness* OR symptom*)) OR AB ((narcotic* OR opiate OR opioid* OR “opioid-related” OR opium OR “opioid-related”) N3 (dependenc* OR abuse OR depression OR disorder*)) OR AB (psychiatry OR orthopsychiatry OR psychiatric* OR psychosis OR psychoses OR encephalopschychosis OR psychotic*)

AND

3. (MH "Psychosocial Intervention") OR (MH "Hospitalization") OR (MH "Treatment Outcomes") OR (MH "Outpatients") OR (MH "Electroconvulsive Therapy") OR (MH "Antianxiety Agents, Benzodiazepine") OR (MH "Diazepam") OR (MH "Haloperidol") OR (MH "Drug Therapy") OR (MH "Administration, Oral") OR (MH "Drug Administration") OR (MH "Administration, Intravenous") OR (MH "Antipsychotic Agents") OR (MH "Restraint, Physical") OR (MH "Patient Seclusion") OR TI (intervention* OR hospitalization OR committed OR “civil commitment” OR outpatient OR “out patient*” OR “out-patient*”) OR TI ((treatment OR “health care” OR healthcare OR “health-care” OR management OR patient OR therap*) N3 outcome*) OR TI (“electroconvulsive therap*” OR “electric convulsive” OR electroconvulsant OR electroshock OR convulsive) OR AB (intervention* OR hospitalization OR committed OR “civil commitment” OR outpatient OR “out patient*” OR “out-patient*”) OR AB ((treatment OR “health care” OR healthcare OR “health-care” OR management OR patient OR therap*) N3 outcome*) OR AB (“electroconvulsive therap*” OR “electric convulsive” OR electroconvulsant OR electroshock OR convulsive) OR TI (benzodiazepine* OR benzodiazepinone*) OR TI (hospital N1 (admission* OR admit*)) OR TI (diazepam OR alboral OR aliseum OR alupram OR amiprol OR ansiolin OR antenex OR anxionil OR apaurin OR apaurine OR “apo-diazepam” OR apozepam OR arzepam OR assival OR atensine OR audium OR azedipamin OR benzopin OR betapam OR bialzepam OR bialzepan OR calmpose OR caudel OR cercin OR cercine OR cersine OR chlordiazepam OR compaz OR desconet OR diaceplex OR dialag OR dialar OR diano OR diapam OR diapanil OR diapax OR diapin OR diapine OR diapo OR diaquel OR diastat OR diazelium OR diazemuls OR diazepan OR diazepen OR diazepin OR diazidem OR dipaz OR dipezona OR dizac OR doval OR drenian OR ducene OR dupin OR duxen OR eridan OR “euphorin p” OR eurosan OR evacalm OR fanstan OR faustan OR gewacalm OR gubex OR kratium OR lamra OR lembrol OR libervant OR lipodiazepam OR lorinon OR lovium OR melode OR mentalium OR methyldiazepinon OR methyldiazepinone OR morosan OR neocalme OR neurolytril OR nivalen OR noan OR novazam OR ortopsique OR paceum OR pacitran OR paxum OR plidan OR propam OR psychopax OR “q-pam” OR radizepam OR relanium OR reliver OR reposepan OR saromet OR sedapam OR seduxen OR serendin OR setonil OR simasedan OR sipam OR sonacon OR stesolid OR stesolin OR “tanquo tablinen” OR tranimul OR tranquirit OR “tranquo puren” OR trazepam OR umbrium OR valaxona OR valiquid OR valium OR valpam OR valrelease OR valtoco OR vanconin OR vatran OR vazen OR vival OR vivol OR zetran) OR TI ((drug OR medicine* OR medication* OR medicament OR pharmacological OR intravenous OR “IV” OR vein OR venous) N3 (administration OR inject* OR “re-administration” OR readministration OR infusion* OR transfusion)) OR TI (haloperidol OR alased OR aloperidin OR aloperidine OR “apo-haloperidol” OR benison OR brotopon OR celenase OR cereen OR cerenace OR cizoren OR depidol OR dores OR dozic OR duraperidol OR fortunan OR govotil OR Haldol OR halidol OR “halo-p” OR halojust OR halomed OR haloneural OR haloper OR “haloperidol hydrochoride” OR “haloperidol intensol” OR “haloperidol lactate” OR haloperil OR haloperin OR haloperitol OR halopidol OR halopol OR halosten OR haricon OR keselan OR linton OR mixidol OR novoperidol OR peluces OR perida OR peridol OR peridor OR selezyme OR seranace OR serenace OR serenase OR serenelfi OR siegoperidol OR sigaperidol OR “trancodol-10” OR “trancodol-5”) OR TI (neuroleptic OR antipsychotic* OR “butyrophenone tranquilizer*” OR “major tranquilizer*” OR “major tranquillizer*” OR neurolepticum OR “phenothiazine tranquilizer*”) OR TI ((physical OR mechanical) N3 restraint*) OR TI (“restraining devic*” OR seclusion OR isolation OR “therapeutic hold*”) OR AB (benzodiazepine* OR benzodiazepinone*) OR AB (hospital N1 (admission* OR admit*)) OR AB (diazepam OR alboral OR aliseum OR alupram OR amiprol OR ansiolin OR antenex OR anxionil OR apaurin OR apaurine OR “apo-diazepam” OR apozepam OR arzepam OR assival OR atensine OR audium OR azedipamin OR benzopin OR betapam OR bialzepam OR bialzepan OR calmpose OR caudel OR cercin OR cercine OR cersine OR chlordiazepam OR compaz OR desconet OR diaceplex OR dialag OR dialar OR diano OR diapam OR diapanil OR diapax OR diapin OR diapine OR diapo OR diaquel OR diastat OR diazelium OR diazemuls OR diazepan OR diazepen OR diazepin OR diazidem OR dipaz OR dipezona OR dizac OR doval OR drenian OR ducene OR dupin OR duxen OR eridan OR “euphorin p” OR eurosan OR evacalm OR fanstan OR faustan OR gewacalm OR gubex OR kratium OR lamra OR lembrol OR libervant OR lipodiazepam OR lorinon OR lovium OR melode OR mentalium OR methyldiazepinon OR methyldiazepinone OR morosan OR neocalme OR neurolytril OR nivalen OR noan OR novazam OR ortopsique OR paceum OR pacitran OR paxum OR plidan OR propam OR psychopax OR “q-pam” OR radizepam OR relanium OR reliver OR reposepan OR saromet OR sedapam OR seduxen OR serendin OR setonil OR simasedan OR sipam OR sonacon OR stesolid OR stesolin OR “tanquo tablinen” OR tranimul OR tranquirit OR “tranquo puren” OR trazepam OR umbrium OR valaxona OR valiquid OR valium OR valpam OR valrelease OR valtoco OR vanconin OR vatran OR vazen OR vival OR vivol OR zetran) OR AB ((drug OR medicine* OR medication* OR medicament OR pharmacological OR intravenous OR “IV” OR vein OR venous) N3 (administration OR inject* OR “re-administration” OR readministration OR infusion* OR transfusion)) OR AB (haloperidol OR alased OR aloperidin OR aloperidine OR “apo-haloperidol” OR benison OR brotopon OR celenase OR cereen OR cerenace OR cizoren OR depidol OR dores OR dozic OR duraperidol OR fortunan OR govotil OR Haldol OR halidol OR “halo-p” OR halojust OR halomed OR haloneural OR haloper OR “haloperidol hydrochoride” OR “haloperidol intensol” OR “haloperidol lactate” OR haloperil OR haloperin OR haloperitol OR halopidol OR halopol OR halosten OR haricon OR keselan OR linton OR mixidol OR novoperidol OR peluces OR perida OR peridol OR peridor OR selezyme OR seranace OR serenace OR serenase OR serenelfi OR siegoperidol OR sigaperidol OR “trancodol-10” OR “trancodol-5”) OR AB (neuroleptic OR antipsychotic* OR “butyrophenone tranquilizer*” OR “major tranquilizer*” OR “major tranquillizer*” OR neurolepticum OR “phenothiazine tranquilizer*”) OR AB ((physical OR mechanical) N3 restraint*) OR AB (“restraining devic*” OR seclusion OR isolation OR “therapeutic hold*”)

**APA PsycINFO**
Date Searched: 7/11/2022
Applied Database Supplied Limits: none
Number of Results: 5,347

Full Search Strategy:

1. (DE "Commitment" OR DE "Outpatient Commitment" OR DE "Commitment (Psychiatric)" OR DE "Involuntary Treatment" OR TI “involuntary*” OR TI ((civil* OR coercive* OR compulsory* OR legal* OR nonvoluntar* OR “non-voluntar*” OR “non voluntary*”) N4 (commitment* OR committed OR treatment* OR hospitalization* OR admission* OR administration OR therap*)) OR AB “involuntary*” OR AB ((civil* OR coercive* OR compulsory* OR legal* OR nonvoluntar* OR “non-voluntar*” OR “non voluntary*”) N4 (commitment* OR committed OR treatment* OR hospitalization* OR admission* OR administration OR therap*)))

AND

1. DE "Addiction" OR DE "Anorexia Nervosa" AND DE "Bulimia" OR DE "Binge Eating Disorder" AND DE "Disruptive Behavior Disorders" OR DE "Behavior Disorders" OR DE "Drug Seeking" AND DE "Impulse Control Disorders" OR DE "Explosive Disorder" AND DE "Suicidal Behavior" OR DE "Suicidality" OR DE "Attempted Suicide" OR DE "Suicidal Ideation" OR DE "Behavioral Health Services" AND DE "Drug Addiction" OR DE "Drug Dependency" OR DE "Eating Disorders" OR DE "Forensic Psychiatry" OR DE "Mental Health" OR DE "Psychiatry" OR DE "Psychosis" OR DE "Serious Mental Illness" OR TI (addiction OR addictive OR “chemical dependenc*” OR anorexia OR bulimia OR diabulimia OR hyperrexia) OR TI ((behavior* OR behaviour*) N3 (health OR disorder* OR aberrant OR aberration OR disturb* OR crisis OR crises OR deviant OR disruptive OR “drug seeking” OR “drug-seeking” OR suicidal OR psychosocial)) OR TI ((reactive OR impulse OR psychosocial OR unipolar) N3 disorder*) OR (psychosocial N2 (disease* OR problem*)) OR TI (“neurobehavioral manifestation*” OR suicidality OR depress*) OR ((drug OR substance OR “substance-related”) N3 (dependen* OR addict* OR facilitation OR habituation OR disorder*)) OR TI (addict OR toxicomani*) OR TI ((eating OR feeding) N3 (disorder* OR behavior* OR behavior* OR patholog*)) OR TI (“forensic psychiat*” OR “insanity defense”) OR TI (commitment N1 “mentally ill”) OR TI ((mental* OR psychic) N3 (health OR care OR condition* OR factor* OR help OR services* OR state OR status OR ill* OR disease* OR abnormal* OR confusion OR defect* OR disorder* OR disturbance* OR insufficienc*)) OR TI (insanity OR psychopathology OR narcotism) OR TI ((neurodevelopmental OR neuropsychiatric OR psychiatric OR psychic OR psychological) N3 (disorder* OR disease* OR disturbance* OR illness* OR symptom*)) OR TI ((narcotic* OR opiate OR opioid* OR “opioid-related” OR opium OR “opioid-related”) N3 (dependenc* OR abuse OR depression OR disorder*)) OR TI (psychiatry OR orthopsychiatry OR psychiatric* OR psychosis OR psychoses OR encephalopschychosis OR psychotic*) OR AB (addiction OR addictive OR “chemical dependenc*” OR anorexia OR bulimia OR diabulimia OR hyperrexia) OR AB ((behavior* OR behaviour*) N3 (health OR disorder* OR aberrant OR aberration OR disturb* OR crisis OR crises OR deviant OR disruptive OR “drug seeking” OR “drug-seeking” OR suicidal OR psychosocial)) OR AB ((reactive OR impulse OR psychosocial OR unipolar) N3 disorder*) OR (psychosocial N2 (disease* OR problem*)) OR AB (“neurobehavioral manifestation*” OR suicidality OR depress*) OR ((drug OR substance OR “substance-related”) N3 (dependen* OR addict* OR facilitation OR habituation OR disorder*)) OR AB (addict OR toxicomani*) OR AB ((eating OR feeding) N3 (disorder* OR behavior* OR behavior* OR patholog*)) OR AB (“forensic psychiat*” OR “insanity defense”) OR AB (commitment N1 “mentally ill”) OR AB ((mental* OR psychic) N3 (health OR care OR condition* OR factor* OR help OR services* OR state OR status OR ill* OR disease* OR abnormal* OR confusion OR defect* OR disorder* OR disturbance* OR insufficienc*)) OR AB (insanity OR psychopathology OR narcotism) OR AB ((neurodevelopmental OR neuropsychiatric OR psychiatric OR psychic OR psychological) N3 (disorder* OR disease* OR disturbance* OR illness* OR symptom*)) OR AB ((narcotic* OR opiate OR opioid* OR “opioid-related” OR opium OR “opioid-related”) N3 (dependenc* OR abuse OR depression OR disorder*)) OR AB (psychiatry OR orthopsychiatry OR psychiatric* OR psychosis OR psychoses OR encephalopschychosis OR psychotic*)

AND

1. (DE "Intervention" AND DE "Hospitalization" OR DE "Psychiatric Hospitalization" AND DE "Treatment Outcomes" OR DE "Psychotherapeutic Outcomes" AND DE "Outpatient Commitment" OR DE "Outpatients” OR DE "Electroconvulsive Shock Therapy" OR DE "Electroconvulsive Shock Therapy" OR DE "Hospital Admission" OR DE "Psychiatric Hospital Admission" OR DE "Benzodiazepines" OR DE "Diazepam" OR DE "Haloperidol" OR DE "Injections" OR DE "Drug Therapy" OR DE "Drug Administration Methods" OR DE "Neuroleptic Drugs" OR DE "Physical Restraint" OR DE "Patient Seclusion" OR TI (intervention* OR hospitalization OR committed OR “civil commitment” OR outpatient OR “out patient*” OR “out-patient*”) OR TI ((treatment OR “health care” OR healthcare OR “health-care” OR management OR patient OR therap*) N3 outcome*) OR TI (“electroconvulsive therap*” OR “electric convulsive” OR electroconvulsant OR electroshock OR convulsive) OR AB (intervention* OR hospitalization OR committed OR “civil commitment” OR outpatient OR “out patient*” OR “out-patient*”) OR AB ((treatment OR “health care” OR healthcare OR “health-care” OR management OR patient OR therap*) N3 outcome*) OR AB (“electroconvulsive therap*” OR “electric convulsive” OR electroconvulsant OR electroshock OR convulsive) OR TI (benzodiazepine* OR benzodiazepinone*) OR TI (hospital N1 (admission* OR admit*)) OR TI (diazepam OR alboral OR aliseum OR alupram OR amiprol OR ansiolin OR antenex OR anxionil OR apaurin OR apaurine OR “apo-diazepam” OR apozepam OR arzepam OR assival OR atensine OR audium OR azedipamin OR benzopin OR betapam OR bialzepam OR bialzepan OR calmpose OR caudel OR cercin OR cercine OR cersine OR chlordiazepam OR compaz OR desconet OR diaceplex OR dialag OR dialar OR diano OR diapam OR diapanil OR diapax OR diapin OR diapine OR diapo OR diaquel OR diastat OR diazelium OR diazemuls OR diazepan OR diazepen OR diazepin OR diazidem OR dipaz OR dipezona OR dizac OR doval OR drenian OR ducene OR dupin OR duxen OR eridan OR “euphorin p” OR eurosan OR evacalm OR fanstan OR faustan OR gewacalm OR gubex OR kratium OR lamra OR lembrol OR libervant OR lipodiazepam OR lorinon OR lovium OR melode OR mentalium OR methyldiazepinon OR methyldiazepinone OR morosan OR neocalme OR neurolytril OR nivalen OR noan OR novazam OR ortopsique OR paceum OR pacitran OR paxum OR plidan OR propam OR psychopax OR “q-pam” OR radizepam OR relanium OR reliver OR reposepan OR saromet OR sedapam OR seduxen OR serendin OR setonil OR simasedan OR sipam OR sonacon OR stesolid OR stesolin OR “tanquo tablinen” OR tranimul OR tranquirit OR “tranquo puren” OR trazepam OR umbrium OR valaxona OR valiquid OR valium OR valpam OR valrelease OR valtoco OR vanconin OR vatran OR vazen OR vival OR vivol OR zetran) OR TI ((drug OR medicine* OR medication* OR medicament OR pharmacological OR intravenous OR “IV” OR vein OR venous) N3 (administration OR inject* OR “re-administration” OR readministration OR infusion* OR transfusion)) OR TI (haloperidol OR alased OR aloperidin OR aloperidine OR “apo-haloperidol” OR benison OR brotopon OR celenase OR cereen OR cerenace OR cizoren OR depidol OR dores OR dozic OR duraperidol OR fortunan OR govotil OR Haldol OR halidol OR “halo-p” OR halojust OR halomed OR haloneural OR haloper OR “haloperidol hydrochoride” OR “haloperidol intensol” OR “haloperidol lactate” OR haloperil OR haloperin OR haloperitol OR halopidol OR halopol OR halosten OR haricon OR keselan OR linton OR mixidol OR novoperidol OR peluces OR perida OR peridol OR peridor OR selezyme OR seranace OR serenace OR serenase OR serenelfi OR siegoperidol OR sigaperidol OR “trancodol-10” OR “trancodol-5”) OR TI (neuroleptic OR antipsychotic* OR “butyrophenone tranquilizer*” OR “major tranquilizer*” OR “major tranquillizer*” OR neurolepticum OR “phenothiazine tranquilizer*”) OR TI ((physical OR mechanical) N3 restraint*) OR TI (“restraining devic*” OR seclusion OR isolation OR “therapeutic hold*”) OR AB (benzodiazepine* OR benzodiazepinone*) OR AB (hospital N1 (admission* OR admit*)) OR AB (diazepam OR alboral OR aliseum OR alupram OR amiprol OR ansiolin OR antenex OR anxionil OR apaurin OR apaurine OR “apo-diazepam” OR apozepam OR arzepam OR assival OR atensine OR audium OR azedipamin OR benzopin OR betapam OR bialzepam OR bialzepan OR calmpose OR caudel OR cercin OR cercine OR cersine OR chlordiazepam OR compaz OR desconet OR diaceplex OR dialag OR dialar OR diano OR diapam OR diapanil OR diapax OR diapin OR diapine OR diapo OR diaquel OR diastat OR diazelium OR diazemuls OR diazepan OR diazepen OR diazepin OR diazidem OR dipaz OR dipezona OR dizac OR doval OR drenian OR ducene OR dupin OR duxen OR eridan OR “euphorin p” OR eurosan OR evacalm OR fanstan OR faustan OR gewacalm OR gubex OR kratium OR lamra OR lembrol OR libervant OR lipodiazepam OR lorinon OR lovium OR melode OR mentalium OR methyldiazepinon OR methyldiazepinone OR morosan OR neocalme OR neurolytril OR nivalen OR noan OR novazam OR ortopsique OR paceum OR pacitran OR paxum OR plidan OR propam OR psychopax OR “q-pam” OR radizepam OR relanium OR reliver OR reposepan OR saromet OR sedapam OR seduxen OR serendin OR setonil OR simasedan OR sipam OR sonacon OR stesolid OR stesolin OR “tanquo tablinen” OR tranimul OR tranquirit OR “tranquo puren” OR trazepam OR umbrium OR valaxona OR valiquid OR valium OR valpam OR valrelease OR valtoco OR vanconin OR vatran OR vazen OR vival OR vivol OR zetran) OR AB ((drug OR medicine* OR medication* OR medicament OR pharmacological OR intravenous OR “IV” OR vein OR venous) N3 (administration OR inject* OR “re-administration” OR readministration OR infusion* OR transfusion)) OR AB (haloperidol OR alased OR aloperidin OR aloperidine OR “apo-haloperidol” OR benison OR brotopon OR celenase OR cereen OR cerenace OR cizoren OR depidol OR dores OR dozic OR duraperidol OR fortunan OR govotil OR Haldol OR halidol OR “halo-p” OR halojust OR halomed OR haloneural OR haloper OR “haloperidol hydrochoride” OR “haloperidol intensol” OR “haloperidol lactate” OR haloperil OR haloperin OR haloperitol OR halopidol OR halopol OR halosten OR haricon OR keselan OR linton OR mixidol OR novoperidol OR peluces OR perida OR peridol OR peridor OR selezyme OR seranace OR serenace OR serenase OR serenelfi OR siegoperidol OR sigaperidol OR “trancodol-10” OR “trancodol-5”) OR AB (neuroleptic OR antipsychotic* OR “butyrophenone tranquilizer*” OR “major tranquilizer*” OR “major tranquillizer*” OR neurolepticum OR “phenothiazine tranquilizer*”) OR AB ((physical OR mechanical) N3 restraint*) OR AB (“restraining devic*” OR seclusion OR isolation OR “therapeutic hold*”)

**ClinicalTrials.gov**
Date Searched: 7/11/2022

Number of Results: 351

Full Search Strategy:

(involuntary OR commitment) AND (psychiatry OR “psychiatric disorders” OR “mental health” OR “behavioral health”) AND (“involuntary treatment*” OR hospitalization)

# 3. Full citation list of included sources in the scoping review

Abukamil, R. : M., Douglas. (2017). Vacating an order for civil commitment. *Journal of The American Academy of Psychiatry and The Law*, *45*(4), 493–495.

Ackerman, M. J. (2010). Essentials of forensic psychological assessment, 2nd ed. In *Essentials of psychological assessment series* (2011-12958-000). John Wiley & Sons Inc. <https://search.ebscohost.com/login.aspx?direct=true&db=psyh&AN=2011-12958-000&site=ehost-live>

ACLP Interdisciplinary Education Subcommittee. (2019, March 15). *ACLP interdisciplinary inpatient collaborative care guide*. Academy of Consultation-Liaison Psychiatry, Inc. <https://www.clpsychiatry.org/wp-content/uploads/ACLP-Interdisciplinary-Inpatient-Collaborative-Care-Guide-2019.pdf>

Albers, D. A., Pasewark, R. A., & Smith, T. C. (1976). Involuntary hospitalization: The social construction of danger. *American Journal of Community Psychology*, *4*(2), 129–132. <https://doi.org/10/b2j94j>

Alexis, A. (1986). Body searches and the right to privacy. *Journal of Psychosocial Nursing and Mental Health Services*, *24*(11), 21–25. <https://doi.org/10/gqs8c9>

Allen, M. H., Currier, G. W., Hughes, D. H., Reyes-Harde, M., Docherty, J. P., & Expert Consensus Panel for Behavioral Emergencies. (2001). The expert consensus guideline series. Treatment of behavioral emergencies. *Postgraduate Medicine*, *Spec No*, 1–88; quiz 89–90.

Allen, N. G., Khan, J. S., Alzahri, M. S., & Stolar, A. G. (2015). Ethical issues in emergency psychiatry. *Emergency Medicine Clinics of North America*, *33*(4), 863–874. <https://doi.org/10/f7zr9b>

APA. (2018, April 19). Involuntary hospitalization. *APA Dictionary of Psychology, American Psychological Association*. <https://dictionary.apa.org/involuntary-hospitalization>

APA. (2020, December). *Position statement on involuntary outpatient commitment and related programs of assisted outpatient treatment (APA official actions)*. <https://www.psychiatry.org/getattachment/d50db97b-59aa-4dd4-a0ec-d09b4e19112e/Position-Involuntary-Outpatient-Commitment.pdf>

APA. (2023, November 15). Voluntary admission. *APA Dictionary of Psychology, American Psychological Association*. <https://dictionary.apa.org/voluntary-admission>

APA Council on Psychiatry and Law. (2020, October). *Resource document on non-emergency involuntary medication for mental disorders in U.S. jails (APA resource document)*. American Psychiatric Association. <https://www.psychiatry.org/getattachment/a49e5fe1-47ec-468a-b56f-072b8f02f4c8/Resource-Document-2020-Non-Emergency-Involuntary-Medication.pdf>

Appelbaum, P. S. (2006). Commentary: Psychiatric advance directives at a crossroads—When can PADs be overridden? *Journal of The American Academy of Psychiatry and The Law*, *34*(3), 395–397.

Ash, P. (2019). From courtroom to clinic: Legal cases that changed mental health treatment. In *From Courtroom to Clinic: Legal Cases that Changed Mental Health Treatment* (p. 170). Cambridge University Press. <https://www.scopus.com/inward/record.uri?eid=2-s2.0-85097696586&doi=10.1017%2f9781108377171&partnerID=40&md5=c493828155d602ba032b3369b742c974>

Barnes, S. S., & Badre, N. (2016). Is the evidence strong enough to warrant long-term antipsychotic use in compulsory outpatient treatment? *Psychiatric Services*, *67*(7), 784–786. <https://doi.org/10/f8x563>

Bartol, C. R. : B., Anne M. (2015). *Psychology and law: Research and practice* (2015-27852-000). Sage Publications, Inc. <https://search.ebscohost.com/login.aspx?direct=true&db=psyh&AN=2015-27852-000&site=ehost-live>

Beighley, P. S., & Brown, G. R. (1992). Medication refusal by psychiatric inpatients in the military. *Military Medicine*, *157*(1), 47–49. <https://doi.org/10/gqs8tt>

Boit, H., Palmer, G. A., & Olson, S. A. (2019). A comparison between the involuntary and voluntary treatment of patients with alcohol use disorder in a residential rehabilitation treatment program. *Journal of Addictions Nursing*, *30*(1), 57–60. <https://doi.org/10/gqs8fq>

Brogdon, M. G. : B., R. :. Adams, J. H. (2004). Psychology and the law. In *Handb. Of Forensic Psychol.: Resour. For Ment. Health and Legal Prof.* (pp. 3–26). Elsevier Inc.

Brooks, R. A. (2007). Psychiatrists’ opinions about involuntary civil commitment: Results of a national survey. *Journal of The American Academy of Psychiatry and The Law*, *35*(2), 219–228.

Brown, M. A., Ridgway, P., Anthony, W. A., & Rogers, E. S. (1991). Comparison of outcomes for clients seeking and assigned to supported housing services. *Hospital and Community Psychiatry*, *42*(11), 1150–1153. <https://doi.org/10/gqs8f6>

Buettner, B. (2017). Older adults in the involuntary treatment system. *Journal of Gerontological Social Work*, *60*(2), 99–103. <https://doi.org/10/gqs8f8>

Byatt, N. : P., Debra A. (2010). Legal and ethical issues in emergency psychiatry. In *Clinical manual of emergency psychiatry.* (2010-09223-012; pp. 261–281). American Psychiatric Publishing, Inc. <https://search.ebscohost.com/login.aspx?direct=true&db=psyh&AN=2010-09223-012&site=ehost-live>

Carroll, P. : M., V. F. (1990). Legal considerations for psychiatric patients. *Advancing Clinical Care : Official Journal of NOAADN*, *5*(6), 16–17.

Cavaiola, A. A., & Dolan, D. (2016). Article commentary: Considerations in civil commitment of individuals with substance use disorders. *Substance Abuse*, *37*(1), 181–187. <https://doi.org/10/gqs8gx>

Chang, J. S., Chiu, J. D., Gruber, V. A., & Sorensen, J. L. (2017). Fair hearing outcomes of patients recommended discharge from methadone maintenance. *Journal of Substance Abuse Treatment*, *83*, 68–72. <https://doi.org/10/gqs8g2>

Charlton, M. : F., Terry L. :. Ivandick, Mark J. (2006). Voluntary or involuntary receipt of state services. In *Law & mental health professionals: Colorado.* (2005-14024-008; pp. 421–452). American Psychological Association. <https://search.ebscohost.com/login.aspx?direct=true&db=psyh&AN=2005-14024-008&site=ehost-live>

Cheung, E. H., Heldt, J., Strouse, T., & Schneider, P. (2018). The medical incapacity hold: A policy on the involuntary medical hospitalization of patients who lack decisional capacity. *Psychosomatics*, *59*(2), 169–176. <https://doi.org/10/gc875g>

Chin, H. P. (2019). Detention, capacity, and treatment in the mentally ill—Ethical and legal challenges. *Cambridge Quarterly of Healthcare Ethics : CQ : The International Journal of Healthcare Ethics Committees*, *28*(4), 752–758. <https://doi.org/10/gqs8g7>

Chodoff, P. : P., R. (1983). The psychiatric will of dr. Szasz. *Hastings Center Report*, *13*(2), 11–13.

Christy, A., Bond, J., & Young, M. S. (2007). Short‐term involuntary examination of older adults in florida. *Behavioral Sciences & The Law*, *25*(5), 615–628. <https://doi.org/10/bb424h>

Christy, A., Boothroyd, R. A., Petrila, J., & Poythress, N. (2003). The reported prevalence of mandated community treatment in two florida samples. *Behavioral Sciences & The Law*, *21*(4), 493–502. <https://doi.org/10/c774j4>

Christy, A., Petrila, J., McCranie, M., & Lotts, V. (2009). Involuntary outpatient commitment in florida: Case information and provider experience and opinions. *Int. J. Forensic Ment. Health*, *8*(2), 122–130. <https://doi.org/10/ccn6n8>

Cochrane, R. E., Herbel, B. L., Reardon, M. L., & Lloyd, K. P. (2013). The sell effect: Involuntary medication treatment is a “clear and convincing” success. *Law and Human Behavior*, *37*(2), 107–116. <https://doi.org/10.1037/lhb0000003>

Compton, S. N., Swanson, J. W., Wagner, H. R., Swartz, M. S., Burns, B. J., & Elbogen, E. B. (2003). Involuntary outpatient commitment and homelessness in persons with severe mental illness. *Mental Health Services Research*, *5*(1), 27–38. <https://doi.org/10/db25wd>

Cranton, J. R. (1968). Involuntary hospitalization of the mentally ill in alabama: A critical analysis. *Journal of the Medical Association of the State of Alabama*, *37*(11), 1266–1271.

Cullen-Drill, M., & Schilling, K. (2008). The case for mandatory outpatient treatment. *Journal of Psychosocial Nursing & Mental Health Services*, *46*(2), 33–41. <https://doi.org/10/b2mwwx>

Davis, G. E. : L., W. E. :. Davis, G. L. (1998). Determining the number of state psychiatric hospital beds by measuring quality of care with artificial neural networks. *American Journal of Medical Quality : The Official Journal of the American College of Medical Quality*, *13*(1), 13–24.

Deaton, R. : B., Harold. (1994). Antipsychotic medication: Regulation through the right to refuse. In *Psychiatric practice under fire: The influence of government, the media, and special interests on somatic therapies.* (1994-98445-004; pp. 85–101). American Psychiatric Association. <https://search.ebscohost.com/login.aspx?direct=true&db=psyh&AN=1994-98445-004&site=ehost-live>

Desmond, D. P., & Maddux, J. F. (1996). Compulsory supervision and methadone maintenance. *Journal of Substance Abuse Treatment*, *13*(1), 79–83. <https://doi.org/10/fnh4bf>

Drogin, E., & Spaderna, C. (2016). Mental illness, dangerousness, and involuntary commitment. In *Gun violence and mental illness.* (2015-55277-007; pp. 159–183). American Psychiatric Association. <https://search.ebscohost.com/login.aspx?direct=true&db=psyh&AN=2015-55277-007&site=ehost-live>

Drude, K. P. (1978). Psychologists and civil commitment: Review of state statutes. *Prof. Psychol. Res. Pract.*, *9*(3), 499–506. <https://doi.org/10/bsnc7v>

Durns, T. A., O’Connell, P. H., Shvartsur, A., Grey, J. S., & Kious, B. M. (2021). Effects of temporary psychiatric holds on length of stay and readmission risk among persons admitted for psychotic disorders. *International Journal of Law and Psychiatry*, *76*. <https://doi.org/10/gqs8kd>

Elbogen, E. B., Swanson, J. W., & Swartz, M. S. (2003). Effects of legal mechanisms on perceived coercion and treatment adherence among persons with severe mental illness. *Journal of Nervous and Mental Disease*, *191*(10), 629–637. <https://doi.org/10/bdxhzk>

Elbogen, E. B. : T., Alan J. (1999). The psychiatric hospital and therapeutic jurisprudence: Applying the law to promote mental health. In *The role of the state hospital in the twenty-first century.* (1999-04443-006; pp. 71–84). Jossey-Bass. <https://search.ebscohost.com/login.aspx?direct=true&db=psyh&AN=1999-04443-006&site=ehost-live>

Fariba, K. : G., Vikas. (2021). *Involuntary commitment: StatPearls*. <http://ovidsp.ovid.com/ovidweb.cgi?T=JS&PAGE=reference&D=medp&NEWS=N&AN=32491309>

Fisher, W. H., Barreira, P. J., Lincoln, A. K., Simon, L. J., White, A. W., & Sudders, M. (2001). Insurance status and length of stay for involuntarily hospitalized patients. *Journal of Behavioral Health Services & Research*, *28*(3), 334–346. <https://doi.org/10.1007/BF02287248>

Fitch, W. L. (2003). Sexual offender commitment in the United States: Legislative and policy concerns. *Ann. New York Acad. Sci.*, *989*((Fitch W.L., fitchl@dhmh.state.md.us) Forensic Services, Department of Health/Mental Hygiene, Jessup, MD 20794-1000, United States), 489–501. <https://doi.org/10/d8vz5b>

Frazier, A. : C., I. (2021). Pre-trial civil commitment of criminal defendants. In *Handb. On Pretrial Justice* (pp. 144–167). Taylor and Francis. <https://www.scopus.com/inward/record.uri?eid=2-s2.0-85131469215&doi=10.4324%2f9781003149842-9&partnerID=40&md5=66da021d7362a02760a6cf513e209d4d>

Friedman, S., Hall, R., & Sorrentino, R. (2018). Involuntary treatment of psychosis in pregnancy. *Journal of The American Academy of Psychiatry and The Law*, *46*(2), 217–223. <https://doi.org/10.29158/JAAPL.003759-18>

Galon, P. A., & Wineman, N. M. (2010). Coercion and procedural justice in psychiatric care: State of the science and implications for nursing. *Archives of Psychiatric Nursing*, *24*(5), 307–316. <https://doi.org/10/dqg2wn>

Garakani, A., Shalenberg, E., Burstin, S. C., Brendel, R. W., & Appel, J. M. (2014). Voluntary psychiatric hospitalization and patient-driven requests for discharge: A statutory review and analysis of implications for the capacity to consent to voluntary hospitalization. *Harvard Review of Psychiatry*, *22*(4), 241–249. <https://doi.org/10/gqs8mn>

Garriga, M., Pacchiarotti, I., Kasper, S., Zeller, S. L., Allen, M. H., Vázquez, G., Baldaçara, L., San, L., McAllister-Williams, R. H., Fountoulakis, K. N., Courtet, P., Naber, D., Chan, E. W., Fagiolini, A., Möller, H. J., Grunze, H., Llorca, P. M., Jaffe, R. L., Yatham, L. N., … Vieta, E. (2016). Assessment and management of agitation in psychiatry: Expert consensus. *World Journal of Biological Psychiatry*, *17*(2), 86–128. <https://doi.org/10.3109/15622975.2015.1132007>

Gaynes, B. N. : B., Carrie :. Lux, Linda J. :. Ashok, Mahima :. Coker-Schwimmer, Emmanuel :. Hoffman, Valerie :. Sheitman, Brian :. Viswanathan, Meera. (2015). *Management strategies to reduce psychiatric readmissions*. <http://ovidsp.ovid.com/ovidweb.cgi?T=JS&PAGE=reference&D=medp&NEWS=N&AN=26020093>

Geller, J. L. (1986). The quandaries of enforced community treatment and unenforceable outpatient commitment statutes. *Journal of Psychiatry & Law*, *14*(1–2), 149–158. <https://doi.org/10/gqs8mt>

Geller, J. L. (1992). Clinical encounters with outpatient coercion at the CMHC: Questions of implementation and efficacy. *Community Mental Health Journal*, *28*(2), 81–94. <https://doi.org/10/b3hvqn>

Geppert, C. M. A. (2022). Civil commitment for substance use disorders: Coercion or compassion? *Psychiatric Times*, *39*(6), 20–23.

Gerbasi, J., Bonnie, R., & Binder, R. (2000). Resource document on mandatory outpatient treatment. *Journal of The American Academy of Psychiatry and The Law*, *28*(2), 127–144.

Gilbert, A. R., Moser, L. L., Van Dorn, R. A., Swanson, J. W., Wilder, C. M., Robbins, P. C., Keator, K. J., Steadman, H. J., & Swartz, M. S. (2010). Reductions in arrest under assisted outpatient treatment in new york. *Psychiatric Services*, *61*(10), 996–999. <https://doi.org/10/dqhdvq>

Griffith, E. E. H., & Etkin, K. (1981). Legal rights and involuntary transfer following voluntary admission. *Hospital and Community Psychiatry*, *32*(5), 319–322. <https://doi.org/10/gqs8nd>

Griffith, E., & Papapietro, D. (2018). Forensic ethics and involuntary outpatient commitment. In *Ethics challenges in forensic psychiatry and psychology practice.* (2018-10500-008; pp. 116–131). Columbia University Press. <https://search.ebscohost.com/login.aspx?direct=true&db=psyh&AN=2018-10500-008&site=ehost-live>

Guidelines for involuntary civil commitment. (1986). *Mental and Physical Disability Law Reporter*, *10*(5), 409–514.

Hardin, K. M., Contreras, I. M., Kosiak, K., & Novaco, R. W. (2022). Anger rumination and imagined violence as related to violent behavior before and after psychiatric hospitalization. *Journal of Clinical Psychology*. <https://doi.org/10/gqs8nm>

Heilbrun, K., & Griffin, P. (1998). Community-based forensic treatment. In *Treatment of offenders with mental disorders.* (1998-07168-004; pp. 168–210). The Guilford Press. <https://search.ebscohost.com/login.aspx?direct=true&db=psyh&AN=1998-07168-004&site=ehost-live>

Hickman, F., Resnick, P., & Olson, K. (1982). Right to refuse psychotropic medication: An interdisciplinary proposal. *Mental Disability Law Reporter*, *6*(2), 122–130.

Hicks, C. : W., S. (2020). Does your patient have the right to refuse medications? A brief guide to the legal aspects of administering involuntary medications. *Curr. Psychiatry*, *19*(4), 22–30.

Hiday, V. A., Swartz, M. S., Swanson, J., & Wagner, H. R. (1997). Patient perceptions of coercion in mental hospital admission. *International Journal of Law and Psychiatry*, *20*(2), 227–241. <https://doi.org/10/czjhz6>

Hoge, S., Applebaum, PS, Lawlor, T, Beck, JC, R, L., Greer, A, Gutheil, TG, & Kaplan, E. (1990). A prospective, multicenter study of patients’ refusal of antipsychotic medication. *Archives of General Psychiatry*, *47*(10), 949–956.

Hoge, S., & Bonnie, R. (2021). Expedited diversion of criminal defendants to court-ordered treatment. *Journal of The American Academy of Psychiatry and The Law*, *49*(4), 517–525. <https://doi.org/10.29158/JAAPL.210076-21>

Hoge, S. K., Lidz, C. W., Eisenberg, M., Gardner, W., Monahan, J., Mulvey, E., Roth, L., & Bennett, N. (1997). Perceptions of coercion in the admission of voluntary and involuntary psychiatric patients. *International Journal of Law and Psychiatry*, *20*(2), 167–181. <https://doi.org/10/bkg3q9>

Holder, S. M., Warren, C., Rogers, K., Griffeth, B., Peterson, E., Blackhurst, D., & Ochonma, C. (2018). Involuntary processes: Knowledge base of health care professionals in a tertiary medical center in upstate south carolina. *Community Mental Health Journal*, *54*(2), 149–157. <https://doi.org/10/gcx98j>

Hopko, D. R., Averill, P. M., Cowan, K., & Shah, N. (2002). Self-reported symptoms and treatment outcome among non-offending involuntary inpatients. *Journal of Forensic Psychiatry*, *13*(1), 88–106. <https://doi.org/10/ddqtxn>

Hunter, R. H., Ritchie, A. J., & Spaulding, W. D. (2005). The sell decision: Implications for psychological assessment and treatment. *Professional Psychology-Research and Practice*, *36*(5), 467–475. <https://doi.org/10/dxkb85>

Irvin, T. (2003). Legal, ethical and clinical implications of prescribing involuntary, life-threatening treatment: The case of the sunshine kid. *Journal of Forensic Sciences*, *48*(4), 856–860. <https://doi.org/10/gqs8pc>

Johnson, J. M., & Stern, T. A. (2014). Involuntary hospitalization of primary care patients: (Rounds in the general hospital). *Primary Care Companion to the Journal of Clinical Psychiatry*, *16*(3). <https://doi.org/10/gqs8pg>

Ladds, B. : C., A. (1994). Involuntary medication of patients who are incompetent to stand trial: A review of empirical studies. *Bulletin of The American Academy of Psychiatry and The Law*, *22*(4), 519–532.

Lareau, C. R. (2013). Civil commitment and involuntary hospitalization of the mentally ill. In *Handbook of psychology: Forensic psychology., Vol. 11, 2nd ed.* (2012-28467-014; pp. 308–331). John Wiley & Sons, Inc. <https://search.ebscohost.com/login.aspx?direct=true&db=psyh&AN=2012-28467-014&site=ehost-live>

Lee, G., & Cohen, D. (2021). Incidences of involuntary psychiatric detentions in 25 U.S. states. *Psychiatric Services*, *72*(1), 61–68. <https://doi.org/10/gqcn56>

Leibman, F. H. : L., Neil. (1991). Developing trends in prisoners’ rights to mental health treatment. *American Journal of Forensic Psychology*, *9*(1), 19–28.

Leifer, R. (1966). Involuntary psychiatric hospitalization and social control. *International Journal of Social Psychiatry*, *13*(1), 53–58.

Leifer, R. (1982). Psychiatry, language and freedom. *Metamedicine*, *3*(3), 397–416. <https://doi.org/10/fph3m4>

Leifer, R. (2018). Involuntary psychiatric hospitalization. *Ethical Human Psychology and Psychiatry*, *19*(3), 182–184. <https://doi.org/10/gqs8qr>

Leong, G. B., & Eth, S. (1991). Ethics in psychiatry. *Current Opinion in Psychiatry*, *4*(6), 872–875. <https://doi.org/10/dctgjz>

Leukefeld, C. G., & Tims, F. M. (1990). Compulsory treatment for drug abuse. *International Journal of The Addictions*, *25*(6), 621–640. <https://doi.org/10/cz2x5r>

Lidz, C. W. : M., E. P. :. Appelbaum, P. S. :. Cleveland, S. (1989). Commitment: The consistency of clinicians and the use of legal standards. *American Journal of Psychiatry*, *146*(2), 176–181.

Lidz, C. W., Mulvey, E. P., Hoge, S. K., Kirsch, B. L., Monahan, J., Bennett, N. S., Eisenberg, M., Gardner, W., & Roth, L. H. (2000). Sources of coercive behaviours in psychiatric admissions. *Acta Psychiatrica Scandinavica*, *101*(1), 73–79. <https://doi.org/10/cq2r4j>

Lozovatsky, M. (2016). Psychiatric involuntary commitment: A brief critique of modern day policy and practice. *Ethics in Biology, Engineering and Medicine*, *7*(1–2), 43–63. <https://doi.org/10/gqs8q7>

Mahler, H., & Co Jr, B. (1984). Who are the “committed”? Update. *Journal of Nervous and Mental Disease*, *172*(4), 189–196.

Maniaci, M. J., Lachner, C., Vadeboncoeur, T. F., Hodge, D. O., Dawson, N. L., Rummans, T. A., Roy, A., & Burton, M. C. (2020). Involuntary patient length-of-stay at a suburban emergency department. *American Journal of Emergency Medicine*, *38*(3), 534–538. <https://doi.org/10/gqs8rg>

Marlowe, D. B. : G., David J. :. Merikle, Elizabeth P. :. Festinger, David S. :. DeMatteo, David S. :. Marczyk, Geoffrey R. :. Platt, Jerome J. (2001). Efficacy of coercion in substance abuse treatment. In *Relapse and recovery in addictions.* (2001-01126-008; pp. 208–227). Yale University Press. <https://search.ebscohost.com/login.aspx?direct=true&db=psyh&AN=2001-01126-008&site=ehost-live>

Martone, C. A. : M., Cameron L. :. Singh, Amarpreet :. Hira-Brar, Shabneet :. Paul, Robindra :. Strassnig, Martin. (2008). Forensic psychiatry—Civil law. In *Oxford American handbook of psychiatry.* (2008-13270-017; pp. 807–885). Oxford University Press. <https://search.ebscohost.com/login.aspx?direct=true&db=psyh&AN=2008-13270-017&site=ehost-live>

McCormack, R. P., Williams, A. R., Goldfrank, L. R., Caplan, A. L., Ross, S., & Rotrosen, J. (2013). Commitment to assessment and treatment: Comprehensive care for patients gravely disabled by alcohol use disorders. *Lancet*, *382*(9896), 995–997. <https://doi.org/10/f2mg3s>

Meloy, J. R. : H., Ansar :. Schiller, Eugene F. (1990). Clinical guidelines for involuntary outpatient treatment. In *Practitioner’s resource series* (1990-97292-000). Professional Resource Exchange, Inc. <https://search.ebscohost.com/login.aspx?direct=true&db=psyh&AN=1990-97292-000&site=ehost-live>

Miller, R. D. (1980). Voluntary “involuntary” commitment—The briar-patch syndrome. *Bulletin of The American Academy of Psychiatry and The Law*, *8*(3), 305–312.

Miller, R. D. (1994). The US Supreme Court looks at voluntariness and consent. *International Journal of Law and Psychiatry*, *17*(3), 239–252. <https://doi.org/10/d66krv>

Miller, R. D., & Fiddleman, P. B. (1984). Outpatient commitment: Treatment in the least restrictive environment? *Hospital and Community Psychiatry*, *35*(2), 147–151. <https://doi.org/10.1176/ps.35.2.147>

Mills, M. J. (1984). Legal issues in psychiatric treatment. *Psychiatric Medicine*, *2*(3), 245–261.

Mindock, S., Wright, K., & Fleming, M. (2012). Prevalence of involuntary commitment for alcohol dependence. *Wisc. Med. J.*, *111*(2), 55–57.

Mueser, K. T. : N., Douglas L. :. Drake, Robert E. :. Fox, Lindy :. Barlow, David H. (2003). Integrated treatment for dual disorders: A guide to effective practice. In *Treatment manuals for practitioners* (2003-07130-000). The Guilford Press. <https://search.ebscohost.com/login.aspx?direct=true&db=psyh&AN=2003-07130-000&site=ehost-live>

Nadelhoffer, T., Bibas, S., Grafton, S., Kiehl, K. A., Mansfield, A., Sinnott-Armstrong, W., & Gazzaniga, M. (2012). Neuroprediction, violence, and the law: Setting the stage. *Neuroethics*, *5*(1), 67–99. <https://doi.org/10/fmtr82>

Nakic, M. (2017). Civil commitment and involuntary outpatient commitment. In *Psychiatry and the Law: Basic Principles* (pp. 63–73). Springer International Publishing.

NASMHPD. (2017, August). *Trend in psychiatric inpatient capacity, united states and each state, 1970 to 2014*. National Association of State Mental Health Program Directors. <https://www.nasmhpd.org/sites/default/files/TACPaper.2.Psychiatric-Inpatient-Capacity_508C.pdf>

Nau, M. : B., H. E. :. Street, J. (2011). Psychotic denial of pregnancy: Legal and treatment considerations for clinicians. *Journal of The American Academy of Psychiatry and The Law*, *39*(1), 31–39.

Noordsy, D. L. : M., Carolyn C. :. Drake, Robert E. (2002). Involuntary interventions in dual disorders programs. In *Ethics in community mental health care: Commonplace concerns.* (2002-00412-007; pp. 95–115). Kluwer Academic/Plenum Publishers. <https://search.ebscohost.com/login.aspx?direct=true&db=psyh&AN=2002-00412-007&site=ehost-live>

Nunley, W. : N., Bernadette :. Cutler, David L. :. Dentinger, Jean :. McFarland, Bentson. (2013). Involuntary civil commitment: Applying evolving policy and legal determination in community mental health. In *Modern community mental health: An interdisciplinary approach.* (2013-08779-004; pp. 49–61). Oxford University Press. <https://search.ebscohost.com/login.aspx?direct=true&db=psyh&AN=2013-08779-004&site=ehost-live>

ONS. (2018). *Psychiatric – mental health nursing orientation guidebook*. Office of Nursing Services (ONS) Mental Health Field Advisory Committee - U.S. Department of Veteran’s Affairs. <https://www.va.gov/covidtraining/docs/ONS_Psych_Mental_Health_Nursing_Orientation_Guide.pdf>

Oriol, M. D., & Oriol, R. D. (1986). Involuntary commitment and the right to refuse medication. *Journal of Psychosocial Nursing and Mental Health Services*, *24*(11), 15–20. <https://doi.org/10/gqs86j>

Parrish, J. (1993). Involuntary use of interventions: Pros and cons. *Innovations & Research*, *2*(1), 15–22.

Parry, J., & Beck, J. (1990). Revisiting the civil commitment/involuntary treatment stalemate using limited guardianship, substituted judgment and different due process considerations: A work in progress. *Mental & Physical Disability Law Reporter*, *14*(2), 102–107.

Parry, J. W. (1994). Involuntary civil commitment in the 90s: A constitutional perspective. *Mental & Physical Disability Law Reporter*, *18*(3), 320–336.

Parsons, P. J. (1984). Norms, commitment, and psychotherapy with the involuntarily hospitalized psychiatric patient. *Psychotherapy Patient*, *1*(2), 81–87. <https://doi.org/10/cpznxk>

Peszke, M. A. (1984). The historic antecedents to the current polemics on involuntary psychiatric hospitalization. *Comprehensive Psychiatry*, *25*(2), 238–246. <https://doi.org/10/d5c352>

Pinals, D. A. (2016). Treatment engagement, access to services, and civil commitment reform: Would these strategies help reduce firearm-related risks? In *Gun violence and mental illness.* (2015-55277-011; pp. 291–315). American Psychiatric Association. <https://search.ebscohost.com/login.aspx?direct=true&db=psyh&AN=2015-55277-011&site=ehost-live>

Pollack, D. A., McFarland, B. H., Mahler, J. M., & Kovas, A. E. (2005). Brief reports: Outcomes of patients in a low-intensity, short-duration involuntary outpatient commitment program. *Psychiatric Services*, *56*(7), 863–866. <https://doi.org/10/cj99b2>

Rachlin, S. (1994). Retention and treatment issues on the psychiatric inpatient unit. In *Psychiatric-legal decision making by the mental health practitioner: The clinician as de facto magistrate.* (1994-97201-004; pp. 77–99). John Wiley & Sons. <https://search.ebscohost.com/login.aspx?direct=true&db=psyh&AN=1994-97201-004&site=ehost-live>

Richmond, J. S. (2021). De-escalation in the emergency department. In *Behavioral emergencies for healthcare providers., 2nd ed.* (2021-36863-021; pp. 221–229). Springer Nature Switzerland AG. <https://search.ebscohost.com/login.aspx?direct=true&db=psyh&AN=2021-36863-021&site=ehost-live>

Robbins, P. C., Keator, K. J., Steadman, H. J., Swanson, J. W., Wilder, C. M., & Swartz, M. S. (2010). Assisted outpatient treatment in new york: Regional differences in new york’s assisted outpatient treatment program. *Psychiatric Services*, *61*(10), 970–975. <https://doi.org/10.1176/appi.ps.61.10.970>

Rosedale, M., & Maher, V. (1993). Anorexia: Enigma and dilemma—Reflections on contemporary clinical interventions. *Journal of the New York State Nurses Association*, *24*(4), 9–14.

Segal, S. P., Laurie, T. A., & Segal, M. J. (2001). Factors in the use of coercive retention in civil commitment evaluations in psychiatric emergency services. *Psychiatric Services*, *52*(4), 514–520. <https://doi.org/10.1176/appi.ps.52.4.514>

Seitler, B. (2008). Once the wheels are in motion: Involuntary hospitalization and forced medicating. *Ethical Human Psychology and Psychiatry*, *10*(1), 31–42. <https://doi.org/10.1891/1559-4343.10.1.31>

Serper, M. R., Goldberg, B. R., Herman, K. G., Richarme, D., Chou, J., Dill, C. A., & Cancro, R. (2005). Predictors of aggression on the psychiatric inpatient service. *Comprehensive Psychiatry*, *46*(2), 121–127. <https://doi.org/10.1016/j.comppsych.2004.07.031>

Simon, R. I. (2001). Concise guide to psychiatry and law for clinicians, 3rd ed. In *Concise guides* (2001-06677-000). American Psychiatric Association. <https://search.ebscohost.com/login.aspx?direct=true&db=psyh&AN=2001-06677-000&site=ehost-live>

Sirica, C. (2000). Outpatient commitment in mental health: Is coercion the price of community services? *Issue Brief (George Washington University. National Health Policy Forum)*, *757*, 1–12.

Skeem, J. L., Louden, J. E., Polaschek, D., & Camp, J. (2007). Assessing relationship quality in mandated community treatment: Blending care with control. *Psychol. Assess.*, *19*(4), 397–410. <https://doi.org/10.1037/1040-3590.19.4.397>

Smith, C. A. (1995). Use of involuntary outpatient commitment in community care of the seriously and persistently mentally ill patient. *Issues in Mental Health Nursing*, *16*(3), 275–284.

Starks, S. L., Kelly, E. L., Castillo, E. G., Meldrum, M. L., Bourgois, P., & Braslow, J. T. (2022). Client outreach in los angeles county’s assisted outpatient treatment program: Strategies and barriers to engagement. *Research On Social Work Practice*. <https://doi.org/10.1177/1049731520949918>

Stern, T. A., Herman, J. B., & Slavin, P. L. (Eds.). (2004). *The massachusetts general hospital guide to primary care psychiatry*. McGraw-Hill Health Professions Division.

Sullivan, M. A., Birkmayer, F., Boyarsky, B. K., Frances, R. J., Fromson, J. A., Galanter, M., Levin, F. R., Lewis, C., Nace, E. P., Suchinsky, R. T., Tamerin, J. S., Tolliver, B., & Westermeyer, J. (2008). Uses of coercion in addiction treatment: Clinical aspects. *American Journal on Addictions*, *17*(1), 36–47. <https://doi.org/10.1080/10550490701756369>

Surya, S., Bishnoi, R. J., & Shashank, R. B. (2019). Balancing medical ethics to consider involuntary administration of electroconvulsive therapy. *Journal of ECT*, *35*(3), 150–151. <https://doi.org/10.1097/YCT.0000000000000624>

Swanson, J., Tong, G., Robertson, A., & Swartz, M. (2020). Gun-related and other violent crime after involuntary commitment and short-term emergency holds. *Journal of The American Academy of Psychiatry and The Law*, *48*(4), 454–467. <https://doi.org/10.29158/JAAPL.200082-20>

Swanson, J. W. : S., M. S. :. George, L. K. :. Burns, B. J. :. Hiday, V. A. :. Borum, R. :. Wagner, H. R. (1997). Interpreting the effectiveness of involuntary outpatient commitment: A conceptual model. *Journal of The American Academy of Psychiatry and The Law*, *25*(1), 5–16.

Swanson, J. W., Swartz, M. S., Elbogen, E. B., Wagner, H. R., & Burns, B. J. (2003). Effects of involuntary outpatient commitment on subjective quality of life in persons with severe mental illness. *Behavioral Sciences & The Law*, *21*(4), 473–491. <https://doi.org/10.1002/bsl.548>

Swanson, J. W., Swartz, M. S., Wagner, H. R., Burns, B. J., Borum, R., & Hiday, V. A. (2000). Involuntary out-patient commitment and reduction of violent behaviour in persons with severe mental illness. *British Journal of Psychiatry*, *176*(APR.), 324–331. <https://doi.org/10.1192/bjp.176.4.324>

Swartz, M. S., Bhattacharya, S., Robertson, A. G., & Swanson, J. W. (2017). Involuntary outpatient commitment and the elusive pursuit of violence prevention: A view from the United States. *Canadian Journal of Psychiatry*, *62*(2), 102–108. <https://doi.org/10.1177/0706743716675857>

Swartz, M. S., Burns, B. J., George, L. K., Swanson, J., Hiday, V. A., Borum, R., & Wagner, H. R. (1997). The ethical challenges of a randomized controlled trial of involuntary outpatient commitment. *Journal of Mental Health Administration*, *24*(1), 35–43. <https://doi.org/10.1007/BF02790478>

Swartz, M. S., Hoge, S. K., Pinals, D. A., Lee, E., Lee, L.-W., Sidor, M., Bell, T., Ford, E., & Scott Johnson, R. (2015). *Resource Document on Involuntary Outpatient Commitment and Related Programs of Assisted Outpatient Treatment*. American Psychiatric Association. <https://www.psychiatry.org/getattachment/685f787b-f08f-4b2c-ac4b-35821d50e4fd/Resource-Document-2015-involuntary-outpatient-commitment.pdf>

Swartz, M. S., & Swanson, J. W. (2013). Economic grand rounds: Can states implement involuntary outpatient commitment within existing state budgets? *Psychiatric Services (Washington, D.C.)*, *64*(1), 7–9. <https://doi.org/10.1176/appi.ps.201200467>

Swartz, M. S., Swanson, J. W., Hiday, V. A., Wagner, H. R., Burns, B. J., & Borum, R. (2001). A randomized controlled trial of outpatient commitment in north carolina. *Psychiatric Services*, *52*(3), 325–329. <https://doi.org/10.1176/appi.ps.52.3.325>

Swartz, M. S., Swanson, J. W., Wagner, H. R., Burns, B. J., & Hiday, V. A. (2001). Effects of involuntary outpatient commitment and depot antipsychotics on treatment adherence in persons with severe mental illness: *Journal of Nervous and Mental Disease*, *189*(9), 583–592. <https://doi.org/10.1097/00005053-200109000-00003>

Swartz, M. S., Swanson, J. W., Wagner, H. R., Hannon, M. J., Burns, B. J., & Shumway, M. (2003). Assessment of four stakeholder groups’ preferences concerning outpatient commitment for persons with schizophrenia. *American Journal of Psychiatry*, *160*(6), 1139–1146. <https://doi.org/10.1176/appi.ajp.160.6.1139>

Swartz, M. S. : W., H. R. :. Swanson, J. W. :. Hiday, V. A. :. Burns, B. J. (2002). The perceived coerciveness of involuntary outpatient commitment: Findings from an experimental study. *Journal of The American Academy of Psychiatry and The Law*, *30*(2), 207–217.

Szasz, T. (2017). Coercion as cure: A critical history of psychiatry. In *Coercion as Cure: A Critical History of Psychiatry* (p. 278). Taylor and Francis. <https://www.scopus.com/inward/record.uri?eid=2-s2.0-85065331660&doi=10.4324%2f9781315080994&partnerID=40&md5=bb78cf88f20f0328c37646232cd37ee9>

Szasz, T. S. (1972). Voluntary mental hospitalization: An unacknowledged practice of medical fraud. *New Engl. J. Med.*, *287*(6), 277–278. <https://doi.org/10.1056/NEJM197208102870604>

Szasz, T. S. (1975). Medicine and madness. *Journal of Psychiatry & Law*, *3*(2), 215–222.

Szasz, T. S. (2005). *The myth of mental illness: Foundations of a theory of personal conduct*. Perennial. <https://search.ebscohost.com/login.aspx?direct=true&db=psyh&AN=2008-13609-035&site=ehost-live>

Taylor, M. F. (2006). Putting values into practice: Involuntary treatment interventions in mental health. In *Community mental health: Challenges for the 21st century.* (2006-05879-007; pp. 73–81). Routledge. <https://search.ebscohost.com/login.aspx?direct=true&db=psyh&AN=2006-05879-007&site=ehost-live>

Torrey, E. F., & Stanley, J. (2013). “assisted outpatient treatment”: An example of newspeak?: In reply. *Psychiatric Services (Washington, D.C.)*, *64*(11), 1179–1180. <https://doi.org/10.1176/appi.ps.641109>

Van Der Veer, N. L., Drachman, D., Ahad, S., Silvers, G., & Ramos, G. (2011). Voluntariness to consent to research in a voluntarily and involuntarily hospitalized psychiatric population. *Journal of Empirical Research on Human Research Ethics*, *6*(1), 55–61. <https://doi.org/10.1525/jer.2011.6.1.55>

VHA. (2013, September 16). *Inpatient mental health services—Vha handbook 1160.06*. Department of Veterans Affairs, Veterans Health Administration.

Wagner, H. R., Swartz, M. S., Swanson, J. W., & Burns, B. J. (2003). Does involuntary outpatient commitment lead to more intensive treatment? *Psychology Public Policy and Law*, *9*(1–2), 145–158. <https://doi.org/10.1037/1076-8971.9.1-2.145>

Walton, M. T., & Hall, M. T. (2017). Involuntary civil commitment for substance use disorder: Legal precedents and ethical considerations for social workers. *Social Work in Public Health*, *32*(6), 382–393. <https://doi.org/10.1080/19371918.2017.1327388>

Warner, M. D. : E., M. L. :. Dorn, M. R. :. Peabody, C. A. (2000). A brief review of the ethical and legal concerns of treating voluntary psychiatric patients on a locked unit. *American Journal of Forensic Psychiatry*, *21*(1), 5–18.

Wasser, T. (2017). Conclusion: How to learn more about forensic psychiatry. In *Psychiatry and the Law: Basic Principles* (pp. 183–188). Springer International Publishing. <https://www.scopus.com/inward/record.uri?eid=2-s2.0-85043334567&doi=10.1007%2f978-3-319-63148-6_16&partnerID=40&md5=acef3bb30638681e701e453193e83bba>

Weinberger, L. E. : M., E. (2016). Mental institutions: Legal issues and commitments. In *Encycl. Of Ment. Health: Second Ed.* (pp. 123–132). Elsevier Inc.

Werth, J. L., Wright, K. S., Archambault, R. J., & Bardash, R. J. (2003). When does the “duty to protect” apply with a client who has anorexia nervosa? *Counseling Psychologist*, *31*(4), 427–450. <https://doi.org/10.1177/0011000003031004006>

Wettstein, R. M. (1999). The right to refuse psychiatric treatment. *Psychiatric Clinics of North America*, *22*(1), 173–182. <https://doi.org/10.1016/S0193-953X(05)70067-8>

Wilk, R. J. (1988a). Implications of involuntary outpatient commitment for community mental health agencies. *American Journal of Orthopsychiatry*, *58*(4), 580–591. <https://doi.org/10.1111/j.1939-0025.1988.tb01622.x>

Wilk, R. J. (1988b). Involuntary outpatient commitment of the mentally III. *Soc. Work*, *33*(2), 133–137. <https://doi.org/10.1093/sw/33.2.133>

Williams, J. B., & Arvidson, M. M. (2021). Resource document for electroconvulsive therapy in adult correctional settings. *Journal of ECT*, *37*(1), 18–23. <https://doi.org/10.1097/YCT.0000000000000694>

Williams, S. N. : J., Melissa G. (2022). Inpatient treatment for african american women. In *A handbook on counseling African American women: Psychological symptoms, treatments, and case studies.* (2022-31334-011; pp. 209–226). Praeger/ABC-CLIO. <https://search.ebscohost.com/login.aspx?direct=true&db=psyh&AN=2022-31334-011&site=ehost-live>

Wilson, M. P., Brennan, J. J., Modesti, L., Deen, J., Anderson, L., Vilke, G. M., & Castillo, E. M. (2015). Lengths of stay for involuntarily held psychiatric patients in the ED are affected by both patient characteristics and medication use. *American Journal of Emergency Medicine*, *33*(4), 527–530. <https://doi.org/10.1016/j.ajem.2015.01.017>

Yarnell, S. : K., R. (2017). Voluntary and involuntary hospitalization. In *Psychiatry and the Law: Basic Principles* (pp. 53–61). Springer International Publishing. <https://www.scopus.com/inward/record.uri?eid=2-s2.0-85043340311&doi=10.1007%2f978-3-319-63148-6_5&partnerID=40&md5=540d7db844fbb4024f80c6015da567c2>

Zilber, C. (2016, November 29). *Ethics considerations of involuntary outpatient treatment*. APA Publishing -  Psychiatric News. <https://doi.org/10.1176/appi.pn.2016.12a16>

# 4. Appendix. Table of data about included sources including citation, evidence type, and excerpt containing the (in)voluntary definition

| **Search Source** | **Title** | **Full citation** | **Year** | **Evidence type** | **Excerpt containing (in)voluntary definition and relevant surrounding text:** | **Number of (in)voluntary definitions** | **Reference citation(s) for the definition, if applicable.** |
| --- | --- | --- | --- | --- | --- | --- | --- |
| Database search | Vacating an order for civil commitment | Abukamil, R. : M., Douglas. (2017). Vacating an order for civil commitment. *Journal of the American Academy of Psychiatry and the Law*, *45*(4), 493–495. | 2017 | review article | Second, C.C. reminds psychiatrists that society views involuntary hospitalization through a special lens: it is not merely an episode of medical treatment, but state-instituted confinement that "can have calamitous effects on an individual... includ[ing] loss of liberty and potential damage to a person's reputation. (C.C., p 108). | 1 | (C.C., p 108); In the Matter of C.C., 376 P.3d 105 (Mont. 2016) |
| Database search | Essentials of Forensic Psychological assessment, Second Edition | Ackerman, M. J. (2010). Essentials of forensic psychological assessment, 2nd ed. In Essentials of psychological assessment series (2011-12958-000). John Wiley & Sons Inc. https://search.ebscohost.com/login.aspx?direct=true&db=psyh&AN=2011-12958-000&site=ehost-live | 2010 | Book excerpt | According to Parry (1994), there are eight basic types of involuntary commitment: ... Voluntary commitment , in which a competent person is admitted voluntarily, but the individual must satisfy certain bureaucratic criteria before being discharged, and/or the facility has a right to initiate involuntary commitment proceedings rather than discharging the individual. | 1 | Parry, J. (1994). Involuntary civil commitment in the 90s: A constitutional perspective. Mental and Physical Disability Law Reporter, 18, 320-333. |
| Hand search organization/agency/association website | ACLP Interdisciplinary Inpatient Collaborative Care Guide | ACLP Interdisciplinary Education Subcommittee. (2019). ACLP Interdisciplinary Inpatient Collaborative Care Guide. Academy of Consultation-Liaison Psychiatry, Inc. https://www.clpsychiatry.org/wp-content/uploads/ACLP-Interdisciplinary-Inpatient-Collaborative-Care-Guide-2019.pdf | 2019 | educational materials | The article begins with discussing the dilemma of using an involuntary psychiatric hold (civil commitment) to hold patients that lack the capacity to leave against medical advice, despite a substantial number of cases in which the patient does not have a psychiatric illness. Medical and legal concerns are reviewed in light of this situation. It is suggested that hospital policies and procedures, including a “medical incapacity hold,” must be in place to detain medically hospitalized patients that lack capacity for medical decision-making. | 1 |  |
| Database search | Involuntary hospitalization: the social construction of danger | Albers, D. A. : P., R. A. :. Smith, T. C. (1976). Involuntary hospitalization: The social construction of danger. American Journal of Community Psychology, 4(2), 129–132. https://doi.org/10/b2j94j | 1976 | research article | Being involuntary, the decision to commit a person to a psychiatric hospital is made by persons other than the proposed patient. The process may be described as follows: Before a person comes to the hearing, someone must file an affidavit containing "evidence" of mental illness. A court order is then issued to the appropriate legal authority, usually the police, requiring the person to appear before the court. After a short examination, the complainant and the defendant, together with his attorney if he has one, appear before the court, which consists of the judge, a court reporter, and the examining physicians. After hearing the evidence, the court decides if the person is mentally ill and should be hospitalized. | 1 |  |
| Database search | Body searches and the right to privacy | Alexis, A. (1986). Body searches and the right to privacy. Journal of Psychosocial Nursing and Mental Health Services, 24(11), 21–25. https://doi.org/10/gqs8c9 | 1986 | review article | An involuntary psychiatric admission is one in which the individual is forced to seek psychiatric treatment, either by the courts, the community, or the family. This admission is necessary because the safety of the per-son/or the community is in danger (Peszke, 1975). Involuntary hospitalization destroys "the right to be let alone" -the most fundamental of all rights. It also abridges the right to physical liberty to associate with persons of one's choice, and to be free from unreasonable searches of one's person. The supreme court has noted, involuntary hospitalization and treatment are a truly "massive" curtailment of liberty (Humphrey v. Cady, 1972). ... A voluntary psychiatric admission is one in which an individual seeks treatment without force or provocation. According to Ennis and Emery (1978), most voluntary patients have almost no understanding of what it means to be a voluntary patient, or of their rights as a voluntary patient. They also believed that voluntary patients are not, in most hospitals, treated any different from involuntary patients. | 2 | Peszke, M.A. Involuntary treatment of the mentally ill: The problem of autonomy. Springfield, Illinois: Charles C. Thomas, 1975. |
| Cited by another source | The Expert Consensus Guideline Series. Treatment of behavioral emergencies | Allen, M. H., Currier, G. W., Hughes, D. H., Reyes-Harde, M., Docherty, J. P., & Expert Consensus Panel for Behavioral Emergencies. (2001). The Expert Consensus Guideline Series. Treatment of behavioral emergencies. *Postgraduate Medicine*, *Spec No*, 1–88; quiz 89–90. | 2001 | Educational materials | Note that by emergency medication, we mean medication given without consent. Voluntary medication refers to medication given with the patient’s assent or consent.  Voluntary Versus Involuntary Treatment- We also asked the experts about what constitutes voluntary treatment (Question 12, p. 55). For the most part, the experts feel that any dose of oral medication to which a patient assents in an emergency situation can be considered voluntary. They rejected the idea that the situation is so coercive that any medication must be considered involuntary even if it the patient appears to accept it. | 2 |  |
| Database search | Ethical Issues in Emergency Psychiatry | Allen, N. G. : K., J. S. :. Alzahri, M. S. :. Stolar, A. G. (2015). Ethical Issues in Emergency Psychiatry. Emergency Medicine Clinics of North America, 33(4), 863–874. https://doi.org/10/f7zr9b | 2015 | review article | Involuntary treatment, which is treatment despite a patient"s refusal, is fraught with ethical and legal challenges (28).... Broad categories of involuntary treatment of relevance to the emergency medicine provider are (1) treatment of emergency medical conditions in patients incapacitated by an acute psychiatric condition; (2) involuntary admission to a mental health facility; (3) testing or medical therapy required to provide medical clearance for psychiatric hospitalization; (4) therapies to control acute behavioral or psychiatric crisis, including medications, seclusion, and restraint.   To admit a patient involuntarily is to deprive that patient of the right to autonomy in the service of beneficence. During involuntary admission, patients are deprived of their autonomy and liberty rights by those in a position of authority; their doctors with the assistance of the state. This deprivation is done with the justification that it is in the patient’s best interest and benefit to do so (ie, medical paternalism), or justified by the need to protect society from the individual (ie, social paternalism).31 Although paternalism has become a pejorative, involuntary admission is an ethically defensible action when used judiciously.32 | 2 | 28. Wettstein RM. The right to refuse psychiatric treatment. Psychiatr Clin North Am 1999;22:173–82, viii. 31. Kjellin L. Medical and social paternalism: regulation of and attitudes towards compulsory psychiatric care. Acta Psychiatr Scand 1993;88(6):415–9.  32. Derse AR. Law and ethics in emergency medicine. Emerg Med Clin North Am 1999;17(2):307–25 |
| Hand search organization/agency/association website | Resource Document on Non-Emergency Involuntary Medication for Mental Disorders in U.S. Jails | APA Council on Psychiatry and Law. (2020). Resource Document on Non-Emergency Involuntary Medication for Mental Disorders in U.S. Jails (APA Resource Document). American Psychiatric Association. https://www.psychiatry.org/getattachment/a49e5fe1-47ec-468a-b56f-072b8f02f4c8/Resource-Document-2020-Non-Emergency-Involuntary-Medication.pdf | 2020 | Educational materials | The use of non-emergency involuntary medication is a clinical and legal process by which a psychiatric patient is administered medication after they have declined acceptance of prescribed medication for the ongoing treatment of a serious mental illness despite adequate efforts to encourage voluntary acceptance of the medications.1 The legal regulation of the administration of non-emergency involuntary medication has historically centered around treatment in hospital settings. General principles across jurisdictions may include requirements including: the patient lacks the capacity required to make psychiatric treatment decisions, the medication be the least restrictive alternative to treat the patient, and/or the patient demonstrates an acute or chronic risk of dangerousness or grave disability as a result of their mental disorder.  The administration of non-emergency involuntary medication is distinct from the administration of emergency involuntary psychiatric medication in situations involving imminent or actual harm to a patient or others. In an emergency, it is generally acceptable, on a one-time basis, to administer the minimum amount of psychiatric medication necessary to mitigate the immediate crisis regardless of the patient’s wishes. This resource document does not address emergency psychiatric medication | 2 |  |
| Hand search organization/agency/association website | Involuntary Hospitalization. APA Dictionary of Psychology | APA. (2018, April 19). Involuntary Hospitalization. APA Dictionary of Psychology, American Psychological Association. https://dictionary.apa.org/involuntary-hospitalization | 2018 | Dictionary entry | involuntary hospitalization Updated on 04/19/2018 the confinement of a person with a serious mental illness to a mental hospital by medical authorization and legal direction (as in involuntary civil commitment). Individuals so hospitalized may be considered dangerous to themselves or others, may fail to recognize the severity of their illness and the need for treatment, or may be unable to have their daily living and treatment needs otherwise met in the community or to survive without medical attention. Compare voluntary admission. | 1 |  |
| Hand search organization/agency/association website | Position Statement on Involuntary Outpatient Commitment and Related Programs of Assisted Outpatient Treatment | APA. (2020). Position Statement on Involuntary Outpatient Commitment and Related Programs of Assisted Outpatient Treatment (APA Official Actions). https://www.psychiatry.org/getattachment/d50db97b-59aa-4dd4-a0ec-d09b4e19112e/Position-Involuntary-Outpatient-Commitment.pdf | 2020 | Position Statement | In this document, the term ‘involuntary outpatient commitment’ is used to refer to outpatient treatment mandated under state involuntary commitment statutes. Involuntary outpatient commitment is a civil court procedure wherein a judge orders a person with severe mental illness to adhere to an outpatient treatment plan designed to prevent relapse and dangerous deterioration. Persons appropriate for this intervention are those who need ongoing psychiatric care owing to severe illness but who are unable or unwilling to engage in ongoing, voluntary, outpatient care. It can be used on release from involuntary hospitalization, an alternative to involuntary hospitalization or as a preventive treatment for those who do not currently meet criteria for involuntary hospitalization. It should be used in each of these instances for patients who need treatment to prevent relapse or behaviors that are dangerous to self or others. | 1 |  |
| Hand search organization/agency/association website | Voluntary Admission. APA Dictionary of Psychology | APA. (2023, November 15). Voluntary Admission. APA Dictionary of Psychology, American Psychological Association. https://dictionary.apa.org/voluntary-admission | 2023 | Dictionary entry | voluntary admission Updated on 11/15/2023 admission of a patient to a psychiatric hospital or other inpatient unit at their own request, without coercion. Such hospitalization can end whenever the patient sees fit, unlike involuntary hospitalization, the length of which is determined by a court or the hospital. Also called voluntary commitment; voluntary hospitalization. | 1 |  |
| Database search | Commentary: Psychiatric advance directives at a crossroads - When can PADs be overridden? | Appelbaum, P. S. (2006). Commentary: Psychiatric advance directives at a crossroads—When can PADs be overridden? *Journal of the American Academy of Psychiatry and the Law*, *34*(3), 395–397. | 2006 | editorials, commentaries, and letters | For the purposes of this discussion, voluntary patients are those who are not subject to either inpatient or outpatient involuntary commitment proceedings at the time their PADs are invoked. | 1 |  |
| Database search | From courtroom to clinic: Legal cases that changed mental health treatment | Ash, P. (2019). From courtroom to clinic: Legal cases that changed mental health treatment. In From Courtroom to Clinic: Legal Cases that Changed Mental Health Treatment (p. 170). Cambridge University Press. https://www.scopus.com/inward/record.uri?eid=2-s2.0-85097696586&doi=10.1017%2f9781108377171&partnerID=40&md5=c493828155d602ba032b3369b742c974 | 2019 | Book excerpt | The case highlighted the differences between psychiatric and other patients. By 1975 it was already clear that medical and surgical hospital patients across the country had the right to refuse prescribed treatment. For psychiatric patients, the situation differed from state to state. In Massachusetts, a patient could either be "voluntary" or be committed to the hospital. Voluntary patients signed a document at the time they were admitted saying that they were willing to accept treatment. When a patient declined to sign, the hospital had a choice to make. It could allow the patient to remain in the community or make the person an "involuntary" patient. The decision was made by hospital staff based on the patient's condition and on what alternatives were available to admission. Involuntary patients were treated against their wishes, including with medication, if staff felt this was clinically what was required. ... A third approach follows the logic of one Wisconsin court that, "Nonconsensual treatment is what involuntary commitment is all about." (44) This approach incorporates incompetence to make treatment decisions into civil commitment criteria (45). Under this scheme, any detained patient has by definition been found by a court to lack competence to refuse treatment. This formula was adopted in the 1970s by Utah. (46) An attempt to implement the proposal in Florida was struck down on the grounds that commitment courts did not have the authority to make competency decisions. (47) Outside the United States practices vary. Utah's solution most closely resembles practice in other Western countries. (48) | 3 | 44. Stensvadv. Reivitz, 601 F. Supp. 128 at 131 45. Stromberg C, Stone A. Statute. A model state law on civil commitment of the mentally ill. Harvard Journal on Legislation 20, 275—396 (1983) |
| Database search | Is the evidence strong enough to warrant long-term antipsychotic use in compulsory outpatient treatment? | Barnes, S. S. : B., N. (2016). Is the evidence strong enough to warrant long-term antipsychotic use in compulsory outpatient treatment? Psychiatric Services, 67(7), 784–786. https://doi.org/10/f8x563 | 2016 | review article | Assisted outpatient treatment (AOT), also called involuntary outpatient commitment, is a treatment modality that has been gaining popularity as public attention paid to high-profile crimes committed by persons with mental illness has in-creased and inpatient resources available to persons with mental illness have decreased (1). In AOT, an individual meeting specified criteria for mental illness is compelled by court order to comply with outpatient psychiatric treatment as a condition of remaining in the community. The two most well-known versions of AOT are Kendra’s Law in New York and Laura’s Law in California, both named for women who were murdered by assailants with mental illness (2). In California, Laura’s Law mandates that patients under AOT be detained, against their will, for evaluation (72-hour psychiatric hold) if they fail to comply with the court-ordered outpatient treatment plan (3). | 1 |  |
| Database search | Psychology and law: Research and practice | Bartol, C. R. : B., Anne M. (2015). Psychology and law: Research and practice (2015-27852-000). Sage Publications, Inc. https://search.ebscohost.com/login.aspx?direct=true&db=psyh&AN=2015-27852-000&site=ehost-live | 2015 | Book excerpt | Involuntary Outpatient Commitments (heading): In some, but not all states, mentally ill individuals are ordered to seek treatment in the community. Also called "assisted commitment" or "nonhospitalization orders," this mechanism is intended to keep individuals out of institutional settings while providing mental health treatment on a regular basis.  Voluntary Commitments (heading): In the 20th century, the literature on civil commitment often made a sharp demarcation between voluntary and involuntary commitment. An estimated 25% to 30% of all patients in public mental institutions were believed to have been committed involuntarily (Monahan & Shah, 1989; Wexler, 1990). Was the majority, then, composed of people who committed themselves on their own volition? Not necessarily, because an unknown number had probably occurred under the threat of formal commitment proceedings (Carroll, 1990). Moreover, some presumably involuntary commitments were actually voluntary. That is, the person wanted to be hospitalized, but the involuntary commitment process was used in order to assure that treatment would be expedited (Farabee, Shen, & Sanchez, 2002). | 2 |  |
| Database search | Medication refusal by psychiatric inpatients in the military | Beighley, P. S. : B., G. R. (1992). Medication refusal by psychiatric inpatients in the military. Military Medicine, 157(1), 47–49. https://doi.org/10/gqs8tt | 1992 | case report | The specific regulation concerning involuntary treatment of active duty personnel is outlined in AFR 168-4(C3) 12-113 b as follows: "When an emergency diagnostic or therapeutic measure is required to preserve the patient's life or limb, it may be performed with or without the patient's permission. It may also be performed when necessary to protect the health or life of a patient who has been declared by a qualified psychiatrist to be mentally incompetent. When time permits, or the situation is less than an emergency, seek counsel from the base staff judge advocate and the area forensic medicine consultant of the legal propriety of the proposed clinical action." | 1 | AFR 168-4 (C3) Section 12-113, Department of the AirForce, Washington. January 19. 1987 |
| Database search | A Comparison Between the Involuntary and Voluntary Treatment of Patients With Alcohol Use Disorder in a Residential Rehabilitation Treatment Program | Boit, H. : P., G. A. :. Olson, S. A. (2019). A Comparison Between the Involuntary and Voluntary Treatment of Patients With Alcohol Use Disorder in a Residential Rehabilitation Treatment Program. Journal of Addictions Nursing, 30(1), 57–60. https://doi.org/10/gqs8fq | 2019 | research article | Involuntary inpatient treatment involves a legal process in which an individual is court-ordered to receive treatment. It may be considered by patients as a form of coercion, in which patients perceive a lack of control over the decision to enter treatment. | 1 |  |
| Database search | Psychology and the Law | Brogdon, M. G. : B., R. :. Adams, J. H. (2004). Psychology and the Law. In *Handb. Of Forensic Psychol.: Resour. For Ment. Health and Legal Prof.* (pp. 3–26). Elsevier Inc. | 2004 | Book excerpt | Involuntary civil commitment is the process by which persons who have been determined to be a danger to themselves or others are hospitalized against their will, even in the absence of the commission of a criminal act. Civil commitment differs from criminal commitment in that the former contemplates a threat of harm in the future, whereas the latter governs confinement for criminal acts already committed. The authority to involuntarily commit dangerous persons derives from the state's police power and parens patriae, that is, the state's sovereign power of guardianship over persons under disability, such as minors and insane and incompetent persons (Freedman, 1988; Black's Law Dictionary, 1968, p. 264). The state's police power authorizes it to enact laws to protect the health, welfare, and safety of the general community. The state's status as parens patriae empowers it to act on behalf of and in the best interest of persons who lack the capacity to do the same. All 50 states and the District of Columbia provide for laws by which an individual may be civilly committed against his or her will in a mental institution based on some showing that the individual is suffering from a mental disorder. Involuntary civil commitment is one of the means by which a psychotherapist may discharge his or her duty to exercise reasonable care to protect third parties against threatened violence confidentially communicated by a patient. The threshold procedural requirements for securing involuntary civil commitment vary greatly from state to state, and practitioners are encouraged to refer to the local law applicable to their practice for the specific procedures and requirements.^ However, the consensus among the states generally limits involuntary commitment to those persons suffering from mental disorders who are diagnosed to be dangerous to themselves or others. | 1 |  |
| Database search | Psychiatrists' opinions about involuntary civil commitment: Results of a national survey | Brooks, R. A. (2007). Psychiatrists’ opinions about involuntary civil commitment: Results of a national survey. *Journal of the American Academy of Psychiatry and the Law*, *35*(2), 219–228. | 2007 | research article | Involuntary civil commitment laws provide for the forceful detention and commitment to an institution by judicial means of persons with mental illnesses who meet certain further criteria. These particular criteria vary from state to state and have also been subject to historical trends. Until the late 1960s many states, operating under their parens patriae powers, allowed for the commitment of persons who had a mental illness and simply needed treatment. Beginning with California in the late 1960s, states began to tighten the criteria for civil commitment in response to reports of abuses, in concern for civil liberties, and in the recognition that, in some circumstances, prolonged inpatient treatment could have harmful consequences.1 In addition, federal policy began to encourage community-based treatment for persons with serious mental disorders. All states adopted some form of the “dangerousness” standard under their police powers, and many states also provided for commitment of persons with mental illnesses who were unable to meet their basic needs (referred to herein as the grave-disability standard).2 | 1 |  |
| Database search | Comparison of outcomes for clients seeking and assigned to supported housing services | Brown, M. A. : R., P. :. Anthony, W. A. :. Rogers, E. S. (1991). Comparison of outcomes for clients seeking and assigned to supported housing services. Hospital and Community Psychiatry, 42(11), 1150–1153. https://doi.org/10/gqs8f6 | 1991 | research article | Although the clients were not "involuntary" in the judicial sense of being legally committed to housing, they were "involuntary" in the programmatic sense of not asking for or seeking out the services (3) Staff had to enter a long process of engagement with this group, persuading them individually to use the supported housing services that they had not voluntarily sought earlier. | 1 | Blanch AK, Parrish J: Report on a Roundtable on Alternatives to Involuntary Treatment held by the National Institute of Mental Health, Sept 14-15, 1990. Rockville, Md, National Institute of Mental Health, 1990 |
| Database search | Older Adults in the Involuntary Treatment System | Buettner, B. (2017). Older Adults in the Involuntary Treatment System. Journal of Gerontological Social Work, 60(2), 99–103. https://doi.org/10/gqs8f8 | 2017 | editorials, commentaries, and letters | The involuntary treatment system allows a state to detain an individual against her or his will, and older adults are often differentially affected‚ especially under grave disability statutes.  involuntary treatment laws, which exist all over the United States, are one of the rare instances in which an individual may be detained by the state without conviction or even suspicion of a crime, because they have a mental illness and pose a risk to themselves or to others.  involuntary treatment statutes extend even further than the imminent risk of harm to self, to allow for the detention of individuals who do not meet their own basic needs. | 3 |  |
| Database search | Legal and ethical issues in emergency psychiatry | Byatt, N. : P., Debra A. (2010). Legal and ethical issues in emergency psychiatry. In Clinical manual of emergency psychiatry. (2010-09223-012; pp. 261–281). American Psychiatric Publishing, Inc. https://search.ebscohost.com/login.aspx?direct=true&db=psyh&AN=2010-09223-012&site=ehost-live | 2010 | Book excerpt | Psychiatric hospitalization is generally intended co stabilize and provide a cherapeucic environment for paciencs, yet it can be perceived as a violation of one's civil liberty when done involuntarily. Psychiatry is distinct from ocher specialties in that it routinely uses involuntary civil commitment as a means co provide intensive, hospital-level care in certain ci rcumstances when persons are in need of such intervention but are refusing voluntary hospicaJizacion. PES staff muse ensure chat proper restrictions on hospitalization are used to preclude the abuse of power related to civil commitment (Lidz et al. 1989).  Conditional voluntary admission (heading): The conditional voluntary status allows the admitting facility to detain patients in the hospital for a period of time, often up to several days, after the patient has announced his or her desire to leave.  Pure Voluntary Admission Under a pure voluntary status, the patient is free to leave the hospital at any time, much like in medical settings. Many states limit pure voluntary status in psychiatric settings, given the higher likelihood that patients who exercise their right to leave might raise enough clinical concern to warrant petitioning for their civil commitment. | 2 |  |
| Database search | Legal considerations for psychiatric patients | Carroll, P. : M., V. F. (1990). Legal considerations for psychiatric patients. *Advancing Clinical Care : Official Journal of NOAADN*, *5*(6), 16–17. | 1990 | review article | A competent mentally ill patient may voluntarily agree to a psychiatric admission for treatment of his/her illness. In many states, this is called a voluntary commitment. Legally this is treated like a typical medical admission, including the patient's right to refuse treatment and a right to a realistic treatment plan with work toward a prompt discharge. As long as patients remain competent, they maintain the right to a voluntary commitment.If, however, a patient is deemed incompetent and of potential danger to self, others, and in some states, property, an involuntary commitment can be set into motion. | 1 |  |
| Database search | Considerations in civil commitment of individuals with substance use disorders | Cavaiola, A. A. : D., D. (2016). Considerations in civil commitment of individuals with substance use disorders. Substance Abuse, 37(1), 181–187. https://doi.org/10/gqs8gx | 2016 | editorials, commentaries, and letters | Civil commitment or involuntary commitment refers to the legal process by which individuals with mental illness are court-ordered into inpatient and/or outpatient treatment programs. Although initially civil commitment laws were intended for individuals with severe mental illness, these statutes have been extended to cover individuals with severe substance use disorders. | 1 |  |
| Database search | Fair hearing outcomes of patients recommended discharge from methadone maintenance | Chang, J. S. : C., J. D. :. Gruber, V. A. :. Sorensen, J. L. (2017). Fair hearing outcomes of patients recommended discharge from methadone maintenance. Journal of Substance Abuse Treatment, 83, 68–72. https://doi.org/10/gqs8g2 | 2017 | research article | Involuntary discharge, when a patient is discharged from a MMT (methadone maintenance treatment) program or clinic against their will, can occur if there is evidence that treatment program rules have been violated, such as selling drugs (methadone or other illicit substances), loitering on the treatment premises, or violent or threatening behavior (Substance Abuse and Mental Health Services Administration, 2015). | 1 | Substance Abuse and Mental Health Services Administration (2015). Federal guidelines for opioid treatment programs. HHS publication no. (SMA)PEP15-FEDGUIDEOTP. Rockville, MD: SAMHSA. |
| Database search | Voluntary or Involuntary Receipt of State Services | Charlton, M. : F., Terry L. :. Ivandick, Mark J. (2006). Voluntary or Involuntary Receipt of State Services. In Law & mental health professionals: Colorado. (2005-14024-008; pp. 421–452). American Psychological Association. https://search.ebscohost.com/login.aspx?direct=true&db=psyh&AN=2005-14024-008&site=ehost-live | 2006 | Book excerpt | The requisite procedural safeguards essential for an involuntary admission distinguish voluntary and involuntary admissions. In general, a person who is a voluntary admission to mental health treatment may seek or quit treatment as he or she chooses. Voluntary patients are accorded all rights and privileges customarily granted to hospital patients (3). A person involuntarily treated for mental illness must have been deemed a danger to self and others and, therefore, forced into treatment accordingly. Once in involuntary treatment, the person may not quit treatment until an MHP or district court has certified the person is no longer a danger to self or others. | 2 | 3. COLO. REV. STAT. § 27-10-103(6). |
| Database search | The Medical Incapacity Hold: A Policy on the Involuntary Medical Hospitalization of Patients Who Lack Decisional Capacity | Cheung, E. H. : H., J. :. Strouse, T. :. Schneider, P. (2018). The Medical Incapacity Hold: A Policy on the Involuntary Medical Hospitalization of Patients Who Lack Decisional Capacity. Psychosomatics, 59(2), 169–176. https://doi.org/10/gc875g | 2018 | Clinical guidelines | We emphasize that the MIH policy as developed by our institution is intended only to articulate the procedures to be followed for the involuntary detention of medically incapacitated patients, and the policy makes no provisions for treatment without consent (involuntary treatment), as these are separate legal and therapeutic issues for which relevant statutes and hospital policies already exist. ... In this absence, psychiatrists are often called upon to issue an in voluntary psychiatric hold ( civil commitment) to keep the patient from leaving. Yet, civil commitment statutes were not intended for, and generally do not address, the needs of the medically ill patient without psychiatric illness. | 2 |  |
| Database search | Detention, Capacity, and Treatment in the Mentally Ill-Ethical and Legal Challenges | Chin, H. P. (2019). Detention, Capacity, and Treatment in the Mentally Ill-Ethical and Legal Challenges. Cambridge Quarterly of Healthcare Ethics : CQ : The International Journal of Healthcare Ethics Committees, 28(4), 752–758. https://doi.org/10/gqs8g7 | 2019 | editorials, commentaries, and letters | For individuals whose mental illness impair their ability to accept appropriate care—the depressed, acutely suicidal mother, or the psychotic lawyer too paranoid to eat any food—statutes exist to permit involuntary hospitalization, a temporary override of paternalistic benefice over personal autonomy. This exception to the primacy of personal autonomy at the core of bioethics has the aim of restoring the mental health of the temporarily incapacitated individual, and with it, their autonomy. Regional distinctions may exist (between countries, or even jurisdictions within a country) regarding applicable mental health laws, or even bioethical ideals strived for (e.g., relatively stronger emphasis on personal autonomy in so-called Western countries), but the tension between autonomy versus paternalism is universal in acute care psychiatry. | 1 |  |
| Database search | The psychiatric will of Dr. Szasz | Chodoff, P. : P., R. (1983). The psychiatric will of Dr. Szasz. The Hastings Center Report, 13(2), 11–13. | 1983 | editorials, commentaries, and letters | Rather, Thomas S. Szasz maintained, these conditions fall within the realm of myth and metaphor; they are moral, not medical problems. Szasz's bold foray against the received wisdom of psychiatry was greeted with protest and indignation, but he was not deterred. Since that time he has become the guru of a movement aiming to destroy the power of psychiatrists to hospitalize anyone involuntarily, or, as he puts it, to subject them to psychiatric slavery, because of "mental illness." | 1 | article is based on "The Myth of Mental Illness," by Thomas Szasz |
| Database search | Short-term involuntary examination of older adults in Florida | Christy, A. : B., J. :. Young, M. S. (2007). Short-term involuntary examination of older adults in Florida. Behavioral Sciences and the Law, 25(5), 615–628. https://doi.org/10/bb424h | 2007 | research article | Involuntary examination (i.e. emergency commitment) under a state"s civil commitment law is one means by which older adults experience assessment for acute mental health care. | 1 |  |
| Database search | The reported prevalence of mandated community treatment in two Florida samples | Christy, A. : B., R. A. :. Petrila, J. :. Poythress, N. (2003). The reported prevalence of mandated community treatment in two Florida samples. Behavioral Sciences and the Law, 21(4), 493–502. https://doi.org/10/c774j4 | 2003 | research article | A review of the Baker Act data base revealed that forty-eight (42.5%) participants in study 2 had experienced a Baker Act (i.e. involuntary psychiatric) examination from May 1999 through December 2001. ... Baker Act examinations are involuntary psychiatric examinations of up to 72 h at approved Baker Act receiving facilities conducted to determine whether the individual meets statutory standards for involuntary civil commitment (see McGaha, Stiles, & Petrila, 2001, for additional details of Baker Act data). | 1 |  |
| Database search | Involuntary outpatient commitment in florida: Case information and provider experience and opinions | Christy, A. : P., J. :. McCranie, M. :. Lotts, V. (2009). Involuntary outpatient commitment in florida: Case information and provider experience and opinions. Int. J. Forensic Ment. Health, 8(2), 122–130. https://doi.org/10/ccn6n8 | 2009 | research article | Referred to by various terms (involuntary outpatient commitment, assisted outpatient treatment, or community treatment orders), IOC is a "statutory and/or court-derived mandate for treatment in the community" with goals of "increasing outpatient treatment compliance, decreasing use of inpatient resources, and improving quality of life for persons with serious mental illness" (Geller, 2006, p. 234). | 1 | Geller, J. L. (2006). The evolution of outpatient commitment in the USA: From conundrum to quagmire. International Journal of Law and Psychiatry, 29, 234-248. |
| Database search | The Sell effect: involuntary medication treatment is a "clear and convincing" success | Cochrane, R. E. : H., B. L. :. Reardon, M. L. :. Lloyd, K. P. (2013). The Sell effect: Involuntary medication treatment is a “clear and convincing” success. *Law and Human Behavior*, *37*(2), 107–116. | 2013 | research article | For the purposes of this study, involuntary treatment was defined as administration of oral or injectable antipsychotic medication occurring after a judicial hearing and court order specifically authorizing such medication be administered against the will of the defendant to treat his mental disorder in order to restore him to competency. Because Federal Bureau of Prisons policies are designed to limit the staff use of force, the actual implementation of involuntary medication under these circumstances is accomplished by the least amount of coercion possible. The preferred method is to persuade the defendant to comply with oral medication or accept long-acting injections of medication without resistance. Only after these interventions are unsuccessful is a Calculated Use of Physical Force sched-uled. A calculated use of force is a security procedure performed by custody staff whereby the defendant is physically restrained, according to Bureau of Prisons policy, in order that administration of medication can be done in a safe and effective manner. | 1 |  |
| Database search | Involuntary outpatient commitment and homelessness in persons with severe mental illness | Compton, S. N. : S., J. W. :. Wagner, H. R. :. Swartz, M. S. :. Burns, B. J. :. Elbogen, E. B. (2003). Involuntary outpatient commitment and homelessness in persons with severe mental illness. Mental Health Services Research, 5(1), 27–38. https://doi.org/10/db25wd | 2003 | research article | Involuntary outpatient commitment (OPC), in which a judge orders patients to adhere to treatment, is designed as a less restrictive alternative to hospitalization for persons with severe mental disorders who are at risk of becoming dangerous or gravely disabled without treatment but reluctant or unable to follow through with community-based treatment. | 1 |  |
| Database search | Involuntary hospitalization of the mentally ill in Alabama: a critical analysis | Cranton, J. R. (1968). Involuntary hospitalization of the mentally ill in Alabama: A critical analysis. *Journal of the Medical Association of the State of Alabama*, *37*(11), 1266–1271. | 1968 | review article | Involuntary hospitalization, or commitment, may be defined essentially as the legal procedure which confines the mentally ill to an appropriately designated hospital. | 1 |  |
| Database search | The case for mandatory outpatient treatment | Cullen-Drill, M. : S., K. (2008). The case for mandatory outpatient treatment. Journal of Psychosocial Nursing & Mental Health Services, 46(2), 33–41. https://doi.org/10/b2mwwx | 2008 | review article | Mandatory outpatient treatment, sometimes referred to as outpatient commitment, involuntary outpatient treatment, or assisted outpatient treatment, involves a court order mandating an individual to follow a treatment plan or become subject to involuntary hospitalization or legal consequences (Torrey & Zdanowicz, 2001). There are two main kinds of mandatory outpatient treatment—diversionary and preventive. Diversionary applies to individuals who meet criteria for involuntary hospitalization but are diverted to less restrictive mandatory outpatient treatment. Preventive applies to those who do not meet criteria for inpatient commitment, but mandatory treatment is sought to prevent further deterioration and their becoming a danger to themselves or society. | 1 | Torrey, E.F., & Zdanowicz, M. (2001). Outpatient commitment: What, why, and for whom. Psychiatric Services, 52, 337-341. |
| Database search | Determining the number of state psychiatric hospital beds by measuring quality of care with artificial neural networks | Davis, G. E. : L., W. E. :. Davis, G. L. (1998). Determining the number of state psychiatric hospital beds by measuring quality of care with artificial neural networks. *American Journal of Medical Quality : The Official Journal of the American College of Medical Quality*, *13*(1), 13–24. | 1998 | research article | Most beds in state hospitals currently are "involuntary" meaning that patients are committed against their will because they are a danger to themselves or others despite the efforts of primary and secondary facilities in the community. The necessity for and the number of involuntary beds are major concerns for mental health planners. | 1 |  |
| Database search | Antipsychotic medication: Regulation through the right to refuse | Deaton, R. : B., Harold. (1994). Antipsychotic medication: Regulation through the right to refuse. In Psychiatric practice under fire: The influence of government, the media, and special interests on somatic therapies. (1994-98445-004; pp. 85–101). American Psychiatric Association. https://search.ebscohost.com/login.aspx?direct=true&db=psyh&AN=1994-98445-004&site=ehost-live | 1994 | Book excerpt | It is worth noting that at least one study (Schwartz et al.1988), although based on a small sample, found that the majority of involuntarily medicated patients, once adequately treated, are approving of the decision to have overridden their treatment refusal and assert that, should they refuse again in similar circumstances, they would wish to be involuntarily medicated again. | 1 | Schwartz HI, Vingiano W, Bezerganian Perez C: Autonomy and the right to refuse treatment: patient's attitudes after involuntary medication. Hosp Community Psychiatry 39:1049-1054, 1988 |
| Database search | Compulsory supervision and methadone maintenance | Desmond, D. P. : M., J. F. (1996). Compulsory supervision and methadone maintenance. Journal of Substance Abuse Treatment, 13(1), 79–83. https://doi.org/10/fnh4bf | 1996 | research article | Subjects were classified as having compulsory supervision if they reported being on parole or probation at admission in either the initial research interview or the follow-up interview. Among those subjects who initially denied parole or probation were 44 who later said that they were on compulsory supervision at admission. Subjects who did not report compulsory supervision in either interview were classified as voluntary. | 1 |  |
| Database search | Mental illness, dangerousness, and involuntary commitment | Drogin, E. Y. : S., Carol. (2016). Mental illness, dangerousness, and involuntary commitment. In Gun violence and mental illness. (2015-55277-007; pp. 159–183). American Psychiatric Association. https://search.ebscohost.com/login.aspx?direct=true&db=psyh&AN=2015-55277-007&site=ehost-live | 2015 | Book excerpt | If mental health professionals determine that someone with serious mental illness may become dangerous but is unwilling to seek treatment, then perhaps the process of involuntary psychiatric commitment could be invoked, confining the individual against his or her wishes to a psychiatric hospital in order to provide treatment and to keep the public safe. ... According to Swartz and Swanson (2013), Involuntary outpatient commitment is a controversial policy that involves providing court-ordered community services to adults with severe mental illness who are nonadherent to treatment. | 2 | Swartz MS, Swanson JW: Economic grand rounds: can states implement involuntary outpatient commitment within existing state budgets? Psychiatr Serv 64(1):7-9, 2013 23280454 |
| Database search | Psychologists and civil commitment: Review of state statutes | Drude, K. P. (1978). Psychologists and civil commitment: Review of state statutes. Prof. Psychol. Res. Pract., 9(3), 499–506. https://doi.org/10/bsnc7v | 1978 | research article | There are basically four types of civil admission/commitment: voluntary, emergency, judicial, and administrative. Voluntary admission/commitment is a nonprotested form of hospitalization initiated by an individual or by someone who can legally act in his or her behalf. Emergency commitment is an involuntary form of detention initiated for immediate detention or hospitalization. The usual criterion for this type of hospitalization is that persons represent a significant or serious potential harm to themselves or others. Judicial commitment typically involves a court that decides upon involuntary hospitalization and often includes evaluations by physicians and/or psychologists as part of that process. The fourth, and much less frequent, type of civil commitment is by an administrative tribunal or lay board. This process involves a group of people outside the judicial system who are empowered to determine whether a person is to be involuntarily hospitalized. | 1 |  |
| Database search | Effects of temporary psychiatric holds on length of stay and readmission risk among persons admitted for psychotic disorders | Durns, T. A. : O., P. H. :. Shvartsur, A. :. Grey, J. S. :. Kious, B. M. (2021). Effects of temporary psychiatric holds on length of stay and readmission risk among persons admitted for psychotic disorders. International Journal of Law and Psychiatry, 76. https://doi.org/10/gqs8kd | 2021 | research article | Because involuntary psychiatric treatment (or treatment over objection) typically cannot occur in Utah or other states in the U.S. unless a patient has already received an involuntary commitment (and generally only after a second hearing that specifically evaluates the involuntary treatment proposed) voluntarily admitted patients may refuse virtually all psychiatric interventions. ... Subjects were defined as admitted voluntarily if there was no record that a temporary hold was issued, or as admitted involuntarily otherwise. | 2 |  |
| Database search | Effects of Legal Mechanisms on Perceived Coercion and Treatment Adherence Among Persons with Severe Mental Illness | Elbogen, E. B. : S., J. W. :. Swartz, M. S. (2003). Effects of Legal Mechanisms on Perceived Coercion and Treatment Adherence Among Persons with Severe Mental Illness. Journal of Nervous and Mental Disease, 191(10), 629–637. https://doi.org/10/bdxhzk | 2003 | research article | Formally mandated community treatment (i.e., involuntary outpatient commitment (OPC)) uses avoidance of hospitalization as leverage to increase adherence to prescribed treatment (Steadman et al., 2001b; Swartz et al., 2001).  One of the more controversial legal mechanisms applied to persons with SMI is involuntary outpatient commitment. Outpatient commitment is a civil procedure in which a judge orders a patient to adhere to treatment in the community.Outpatient commitment is designed as a less restrictive alternative to hospitalization for persons with SMI who are at risk of becoming dangerous or gravely disabled without treatment but are reluctant or unable to follow through with community-based treatment. | 1 | Steadman H, Gounis K, Dennis D, Hopper K, Roche B, Swartz M, Robbins PC (2001b) Assessing the New York City involuntary outpatient commitment pilot program. Psychiatr Serv. 52:330 ‚Äì336.  Swartz MS, Swanson JW, Wagner HR, Burns BJ, Hiday VA (2001) Effects of involuntary outpatient commitment and depot antipsychotics on treatment adherence in persons with severe mental illness. J Nerv Ment Dis. 189:583‚Äì592. |
| Database search | The psychiatric hospital and therapeutic jurisprudence: Applying the law to promote mental health | Elbogen, E. B. : T., Alan J. (1999). The psychiatric hospital and therapeutic jurisprudence: Applying the law to promote mental health. In The role of the state hospital in the twenty-first century. (1999-04443-006; pp. 71–84). Jossey-Bass. https://search.ebscohost.com/login.aspx?direct=true&db=psyh&AN=1999-04443-006&site=ehost-live | 1999 | Book excerpt | Currently, statutory provisions exist for some form of involuntary outpatient commitment (IOC) in at least forty states (McCafferty and Dooley, 1990). IOC compels persons with mental illness who need treatment to submit to treatment on an outpatient basis either as an alternative to psychiatric hospitalization or after discharge from inpatient psychiatric facilities (for an overview,see Hiday, 1996). | 1 | Hiday, V. "Outpatient Commitment: Official Coercion in the Community." In D. L. Dennis and J. Monahan (eds.), Coercion and Aggressive Community Treatment: A New Frontier in Mental Health Law. New York: Plenum, 1996. |
| Database search | Involuntary Commitment : StatPearls | Fariba, K. : G., Vikas. (2021). Involuntary Commitment: StatPearls. http://ovidsp.ovid.com/ovidweb.cgi?T=JS&PAGE=reference&D=medp&NEWS=N&AN=32491309 | 2021 | review article | Varying by title and subtle nuances, civil commitment is ubiquitous throughout the practice of psychiatry. Defined by the United States Health and Human Services, civil commitment - involuntary hospitalization of a patient - is the legal process by which a person is confined in a psychiatric hospital because of a treatable mental disorder, against his or her wishes. | 1 | United States Health and Human Services (no specific citation provided) |
| Database search | Insurance status and length of stay for involuntarily hospitalized patients | Fisher, W. H. : B., P. J. :. Lincoln, A. K. :. Simon, L. J. :. White, A. W. :. Roy-Bujnowski, K. :. Sudders, M. (2001). Insurance status and length of stay for involuntarily hospitalized patients. *Journal of Behavioral Health Services and Research*, *28*(3), 334–346. | 2001 | research article | Because admission under 'conditional voluntary' status alluded to all above triggers a different type of decision making around discharge and other factors, only 'true involuntary' patients (i.e. those committed under Section 12b and remaining under that status for at least 24 hours before accepting a conditional voluntary stay) were included in the study. | 1 |  |
| Database search | Sexual Offender Commitment in the United States: Legislative and Policy Concerns | Fitch, W. L. (2003). Sexual offender commitment in the United States: Legislative and policy concerns. Ann. New York Acad. Sci., 989((Fitch W.L., fitchl@dhmh.state.md.us) Forensic Services, Department of Health/Mental Hygiene, Jessup, MD 20794-1000, United States), 489–501. https://doi.org/10/d8vz5b | 2000 | review article | Every state has a law for the civil commitment (involuntary hospitalization) of people with a mental illness. Generally reserved for individuals with serious psychiatric disorders like schizophrenia or bipolar disorder, these laws are used in most states only when an individual's symptoms become acute and place the individual at imminent risk of serious harm. Once a committed patient's symptoms begin to remit (and the risks abate), he or she ordinarily is discharged, typically with a referral for "aftercare" services in the community. Hospital stays for such patients rarely exceed 30 days, although some require rehospitalization periodically. | 1 |  |
| Database search | Pre-Trial Civil Commitment of Criminal Defendants | Frazier, A. : C., I. (2021). Pre-Trial Civil Commitment of Criminal Defendants. In Handb. On Pretrial Justice (pp. 144–167). Taylor and Francis. https://www.scopus.com/inward/record.uri?eid=2-s2.0-85131469215&doi=10.4324%2f9781003149842-9&partnerID=40&md5=66da021d7362a02760a6cf513e209d4d | 2001 | Book excerpt | Involuntary commitment is a legal intervention by the judicial system whereby a person with a serious mental illness or substance use problem, who is deemed in need of evaluation, supervision, or intervention, may be confined to a treatment facility for a specified period of time - typically between six months to one year (Chambers, 1972; SAMHSA, 2019). While most commitment statutes include a limit to the duration of confinement, some statutes do not set such a limit, allowing for indefinite confinement or an unlimited number of consecutive commitments (Banks, 1986; Rowe, 2017). At times, confinement under civil commitment statutes may include involuntary medication or other treatment, though treatment is not always required or provided (Glasgow, 2004). Although commitment standards, eligibility criteria, and duration of confinement vary across jurisdictions, the vast majority of jurisdictions require a determination of mental illness or abnormality, as well as a high likelihood of resulting harm that cannot be mitigated by other means (Gordon, 2016; Jackson v. Indiana, 1972). | 1 | Chambers, D. L. (1972) Alternatives to civil commitment of the mentally ill: Practical guides and constitutional imperatives. Michigan Law Review, 70, 1107‚Äì200.  Substance Abuse and Mental Health Services Administration (2019). Civil commitment and the mental health care continuum: Historical trends and principles for law and practice. Office of the Chief Medical Officer, Substance Abuse and Mental Health Services Administration. www.samhsa.gov/sites/default/files/civilcommitment-continuum-of-care.pdf |
| Database search | Involuntary Treatment of Psychosis in Pregnancy | Friedman, S. H. : H., R. C. W. :. Sorrentino, R. M. (2018). Involuntary Treatment of Psychosis in Pregnancy. The Journal of the American Academy of Psychiatry and the Law, 46(2), 217–223. https://doi.org/10.29158/JAAPL.003759-18 | 2018 | review article | Historically involuntary treatment referred to the judicial authorization of antipsychotic treatment in hospitalized mentally ill patients who lacked capacity to make medical decisions. Today, there are significant jurisdictional differences regarding authorization of involuntary antipsychotic treatment in the community. | 1 |  |
| Database search | Coercion and Procedural Justice in Psychiatric Care: State of the Science and Implications for Nursing | Galon, P. A. : W., N. M. (2010). Coercion and Procedural Justice in Psychiatric Care: State of the Science and Implications for Nursing. Archives of Psychiatric Nursing, 24(5), 307–316. https://doi.org/10/dqg2wn | 2010 | review article | In 2009, O'Brien called on the international behavioral health nursing community to debate the merits of involuntary outpatient treatment, a form of coercive treatment referred to in the United States as OPC, and join in the burgeoning research related to coercion (O'Brien, 2009). | 1 | O'Brien, A. (2009). Nurses need to debate involuntary outpatient treatment. Archives of Psychiatric Nursing, 23, 89. |
| Database search | Voluntary psychiatric hospitalization and patient-driven requests for discharge: A statutory review and analysis of implications for the capacity to consent to voluntary hospitalization | Garakani, A. : S., E. :. Burstin, S. C. :. Brendel, R. W. :. Appel, J. M. (2014). Voluntary psychiatric hospitalization and patient-driven requests for discharge: A statutory review and analysis of implications for the capacity to consent to voluntary hospitalization. Harvard Review of Psychiatry, 22(4), 241–249. https://doi.org/10/gqs8mn | 2014 | review article | However, while termed "voluntary," these inpatient psychiatric admissions commonly abridge autonomy regarding discharge and other rights. For example, in attempting to balance autonomy and the safety of patients and others, most jurisdictions employ "formal" voluntary admissions: patients petition for admission under a series of specified, state-variable criteria but, in so doing, agree to relinquish their rights to immediate discharge for specified time periods (hours to days, depending on the state).12 If the patient requests discharge, the treating institution must determine whether the patient is safe for discharge and then either discharge the patient within a prescribed time period or, if longer hospitalization is warranted, obtain an involuntary commitment authorization, often from a court. A small number of U.S. states and facilities also permit "informal" voluntary admissions, in which the patient need not meet criteria established by state law for admission, is not bound by any formal agreement, and must be permitted to leave upon request.13-23 | 2 | 12. Gilboy JA, Schmidt JR. "Voluntary" hospitalization of the mentally ill. Northwest Univ Law Rev 1971;66:429-53. 13. Appelbaum PS, Gutheil TG. Clinical handbook of psychiatry and the law. Philadelphia: Lippincott Williams & Wilkins, 2007. 14. Gilboy JA. Informal admission of patients to state psychiatric institutions. Am J Orthopsychiatry 1977;47:321-30. 15. Owens H. When is a voluntary commitment really voluntary? Am JOrthopsychiatry 1977;47:104-10. 16. Conn. Gen. Stat. S17a-506 (2013). 17. 405 IlI. Comp. Stat. S5/3-300 (2012). 18. La. Rev. Stat. Ann. S28:52 (2012). 19. Me. Rev. Stat. Ann. tit. 34-B, $3831 (2013). 20. Md. Code, Health Law $10-609 (2012). 21. Mich. Comp. Laws $330.1411 (2012). 22. N.Y. Mental Hyg. Law S9.15 (Consol. 2012). 23. Okla. Stat. tit. 3A, $5-302 (2012). |
| Database search | Assessment and management of agitation in psychiatry: Expert consensus | Garriga, M. : P., I. :. Kasper, S. :. Zeller, S. L. :. Allen, M. H. :. Vázquez, G. :. Baldacąra, L. :. San, L. :. McAllister-Williams, R. H. :. Fountoulakis, K. N. :. Courtet, P. :. Naber, D. :. Chan, E. W. :. Fagiolini, A. :. Möller, H. J. :. Grunze, H. :. Llorca, P. M. :. Jaffe, R. L. :. Yatham, L. N. :. Hidalgo-Mazzei, D. :. Passamar, M. :. Messer, T. :. Bernardo, M. :. Vieta, E. (2016). Assessment and management of agitation in psychiatry: Expert consensus. World Journal of Biological Psychiatry, 17(2), 86–128. https://doi.org/10.3109/15622975.2015.1132007 | 2016 | Clinical guidelines | Allen et al. (2001), recommend verbal intervention or voluntary medication (medication given with the patient's consent) before moving to more intrusive strategies. | 1 | Allen MH, Currier GW, Hughes DH, Reyes-Harde M, Docherty JP. 2001. The Expert Consensus guideline series. Treatment of behavioral emergencies. Postgrad Med. 1;88. quiz 89-90. Available from: http://www.ncbi.nlm.nih.gov/pubmed/ 11500996 |
| Database search | Management Strategies To Reduce Psychiatric Readmissions | Gaynes, B. N. : B., Carrie :. Lux, Linda J. :. Ashok, Mahima :. Coker-Schwimmer, Emmanuel :. Hoffman, Valerie :. Sheitman, Brian :. Viswanathan, Meera. (2015). Management Strategies To Reduce Psychiatric Readmissions. http://ovidsp.ovid.com/ovidweb.cgi?T=JS&PAGE=reference&D=medp&NEWS=N&AN=26020093 | 2015 | technical brief | Involuntary outpatient commitment or compulsory treatment orders: Involuntary OPC, existing in the United States, and CTOs, existing abroad, are legal orders that compel individuals with mental illness to engage in outpatient treatment to avoid future rehospitalization (66-75). | 1 | 66. Burns I, Rugkasa J, Molodynski A, et al. Community treatment orders for patients with psychosis (OCTET): a randomised controlled trial. Lancet. 2013 May 11;381(9878):1627-33. Epub: 2013/03/30. PMID: 23537605. 67. Fernandez GA, Nygard S. Impact of involuntary outpatient commitment on the revolving-door syndrome in North Carolina. Hosp Community Psychiatry. 1990 Sep;41(9):1001-4. Epub: 1990/09/01. PMID: 2210692. 68. Frank D, Perry JC, Kean D, et al. Effects of compulsory treatment orders on time to hospital readmission. Psychiatr Serv. 2005 Jul;56(7):867-9. Epub: 2005/07/16. PMID: 16020822. 69. Kisely S, Preston N, Xiao J, et al. An eleven-year evaluation of the effect of community treatment orders on changes in mental health service use. J Psychiatr Res. 2013 May;47(5):650-6. Epub: 2013/02/19. PMID: 23415453. 70. Nakhost A, Perry JC, Frank D. Assessing the outcome of compulsory treatment orders on management of psychiatric patients at 2 McGill University-associated hospitals. Can J Psychiatry. 2012 Jun;57(6):359-65. Epub: 2012/06/12. PMID: 22682573. 71. Segal SP, Burgess P. Extended outpatient civil commitment and treatment utilization. Soc Work Health Care. 2006;43(2-3):37-51. Epub: 2006/09/08. PMID: 16956852. 72. Swartz MS, Swanson JW, Wagner HR, et al. Can involuntary outpatient commitment reduce hospital recidivism?: Findings from a randomized trial with severely mentally ill individuals. Am J Psychiatry. 1999 Dec;156(12):1968-75. Epub: 1999/12/10. PMID: 10588412. 73. Compton SN, Swanson JW, Wagner HR, et al. Involuntary outpatient commitment and homelessness in persons with severe mental illness. Ment Health Serv Res. 2003 Mar;5(1):27-38. Epub: 2003/02/27. PMID: 12602644. 74. Vaughan K, McConaghy N, Wolf C, et al. Community treatment orders: relationship to clinical care, medication compliance, behavioural disturbance and readmission. Aust N Z J Psychiatry. 2000 Oct;34(5):801- 8. Epub: 2000/10/19. PMID: 11037366. 75. Hiday VA, Scheid-Cook TL. Outpatient commitment for "revolving door" patients: compliance and treatment. J Nerv Ment Dis. 1991 Feb;179(2):83-8. Epub: 1991/02/01. PMID: 1990075. |
| Database search | The quandaries of enforced community treatment and unenforceable outpatient commitment statutes | Geller, J. L. (1986). The quandaries of enforced community treatment and unenforceable outpatient commitment statutes. Journal of Psychiatry and Law, 14(1–2), 149–158. https://doi.org/10/gqs8mt | 1986 | research article | The dilemma facing contemporary American psychiatry-one which is both practical and moral-is how to address the needs of the casualties of deinstitutionalization without resurrecting the ruins of the state hospitals. One partial solution is involuntary outpatient commitment, a judicial process which results in coerced treatment in the community.2 The debate over the advisability and efficacy of outpatient commitment has begun. 3This article examines the quandaries posed by enforced community treatment and unenforced outpatient commitment statutes. 'One suggested component of a comprehensive system of community mental health services has been coercive or involuntary outpatient treatment, referred to in many of its forms as outpatient commitment. | 1 | V. A. Hiday and R. R. Goodman, "The Least Restrictive Alternative to Involuntary Hospitalization, Outpatient Commitment: Its Use and Effectiveness," 10 Journal of Psychiatry and Law 81-96 (1982); R. D. Miller and P B. Fiddleman, "Outpatient Commitment: Treatment in the Least Restrictive Environment?", 35 Hospital and Community Psychiatry 147-51 (1984); D. Band, A. Heine, J.Goldfrartk- et al., "Outpatient Commitment: A 13-Year Experience" (Paper presented at 15th Annual Meeting AAPL, Nassau, The Bahamas, October 25-28, 1984); R. D. Miller, "Commitment to Outpatient Treatment: A National Survey," 36 Hospital and Community Psychiatry 265-67 (1985); K. Schneider-Braus, "Civil Commitment to Outpatient Psychotherapy: A Case Study" (Paper presented at 16th Annual Meeting AAPL, Albuquerque, N.M., October 16-20, 1985) |
| Database search | Clinical encounters with outpatient coercion at the CMHC: Questions of implementation and efficacy | Geller, J. L. (1992). Clinical encounters with outpatient coercion at the CMHC: Questions of implementation and efficacy. Community Mental Health Journal, 28(2), 81–94. https://doi.org/10/b3hvqn | 1992 | review article | Clinical encounters with three outpatients at a CMHC who were managed with alternating periods of voluntary or uncoerced and involuntary or coerced treatment are presented. ... The treatment was involuntary or coercive in its structure because the patients had no alternative site for treatment other than the CMHC; they had no options for treatment at the CMHC other than the model outlined; they had no choice of psychiatrist; noncompliance resulted in commitment; and they could not initiate changes in their treatment plan until they had been out of the hospital at least one year, a condition imposed upon them. | 2 |  |
| Database search | Civil Commitment for Substance Use Disorders: Coercion or Compassion? | Geppert, C. M. A. (2022). Civil Commitment for Substance Use Disorders: Coercion or Compassion? *Psychiatric Times*, *39*(6), 20–23. | 2022 | editorials, commentaries, and letters | As of May 2021, if Jeff is a resident of 34 states or DC, the psychiatrist will be able to tell Dr F that civil commitment is a possibility. The Prescription Drug Abuse Policy System (PDAPS) defines substance use laws for involuntary commitment as follows: "Involuntary commitment is a legal process through which an individual who is deemed by a qualified agent to have symptoms of substance use disorder is ordered by a court into treatment in an inpatient setting." The PDAPS identified the jurisdictions where SUDs may meet criteria for civil commitment (Figure 1).11 In other states, like Arizona and Arkansas, substance use is specifically excluded from the definition of mental illness utilized in the involuntary commitment statute.8 Civil commitment for SUD is distinct from involuntary commitment for mental illness and from legally mandated treatment in a criminal justice context, such as through drug courts. Psychiatrists know well that many involuntarily committed patients have cooccurring addiction and mental illness.' ... It is no wonder that there has been growing interest in laws authorizing civil (involuntary) commitment for SUDs. | 2 | The Prescription Drug Abuse Policy System (PDAPS) |
| Database search | Resource document on mandatory outpatient treatment | Gerbasi, J. B. : B., R. J. :. Binder, R. L. (2000). Resource document on mandatory outpatient treatment. *The Journal of the American Academy of Psychiatry and the Law*, *28*(2), 127–144. | 2000 | Educational materials | Although most statutes and much of the literature use the term outpatient commitment, many psychiatrists prefer other phrases, such as "mandatory outpatient treatment" or "assisted outpatient treatment" to refer to this practice. The phrase "outpatient commitment" implies a much more coercive approach than is envisioned by proponents of judicial treatment orders or directives. In practice, these devices are used primarily to reinforce the patient's own resolve and are not imposing treatment against the patient's will (the idea ordinarily conveyed by the term "involuntary"). Indeed, the use of therapeutic leverage for psychiatric patients closely resembles the tools (such as "directly observed treatment" for patients with tuberculosis) sometimes used on an outpatient basis for patients with contagious diseases, a context in which the term "commitment" is never used. In this Resource Document, whenever appropriate, the phrase "mandatory outpatient treatment" will be used in preference to "outpatient commitment. | 1 |  |
| Database search | Reductions in arrest under assisted outpatient treatment in New York | Gilbert, A. R. : M., L. L. :. Van Dorn, R. A. :. Swanson, J. W. :. Wilder, C. M. :. Robbins, P. C. :. Keator, K. J. :. Steadman, H. J. :. Swartz, M. S. (2010). Reductions in arrest under assisted outpatient treatment in New York. Psychiatric Services, 61(10), 996–999. https://doi.org/10/dqhdvq | 2010 | research article | New York State"s assisted outpatient treatment (AOT) program, and the intensive services provided under the program, could decrease criminal justice involvement for adults with serious mental illness. AOT is court-mandated treatment designed for individuals who are unlikely to live safely in the community without supervision and who are also unlikely to voluntarily participate in treatment. The court order stipulates that intensive case management services must be provided to individuals while they are in the AOT program. Under an alternative arrangement, some individuals for whom an AOT order is pursued in court sign a voluntary service agreement in lieu of a formal court order. Under a voluntary agreement, the individual is directed to the same array of services as those who receive an AOT order; however, the individual signs a statement that he or she will adhere to a prescribed community treatment plan rather than receiving a mandate from the court. | 1 |  |
| Database search | Legal rights and involuntary transfer following voluntary admission | Griffith, E. E. H. : E., K. (1981). Legal rights and involuntary transfer following voluntary admission. Hospital and Community Psychiatry, 32(5), 319–322. https://doi.org/10/gqs8nd | 1981 | research article | Connecticut law provides for two kinds of voluntary admission to psychiatric facilities. The first is termed an informal admission and requires no written application from the patient, who is free to leave the hospital at any time. The second, termed a voluntary admission, requires that a patient apply in writing for admission for observation and treatment. Under this voluntary status, the patient must give written notice of his desire to leave. After such notice is received, the institution may not confine him for more than five working days unless an application for commitment has been filed with the probate court. According to the law, the patient must be notified on admission of these restrictions on his right to leave (3).' | 2 | 3 Connecticut General Statutes. 17-187 |
| Database search | Forensic ethics and involuntary outpatient commitment | Griffith, E. E. H. : P., Daniel. (2018). Forensic ethics and involuntary outpatient commitment. In Ethics challenges in forensic psychiatry and psychology practice. (2018-10500-008; pp. 116–131). Columbia University Press. https://search.ebscohost.com/login.aspx?direct=true&db=psyh&AN=2018-10500-008&site=ehost-live | 2015 | Book excerpt | The American Psychiatric Association (2015, 1) defined IOC as "a civil court procedure wherein a judge orders a person with severe mental illness to adhere to an outpatient treatment plan designed to prevent relapse and dangerous deterioration | 1 | American Psychiatric Association. 2015. "Position Statement on Involuntary Outpatient Commitment and Related Programs of Assisted Outpatient Treatment." |
| Database search | Guidelines for involuntary civil commitment | Guidelines for involuntary civil commitment. (1986). *Mental and Physical Disability Law Reporter*, *10*(5), 409–514. | 1986 | Clinical guidelines | Involuntary civil commitment is the legal, medical, and psychosocial process whereby a person deemed to be men tally ill and dangerous to self or others and in need of treatment is forced into involuntary mental health care. The ways in which this authority is exercised reflect dif ferent combinations of legal criteria, which establish the situations and characteristics of persons subject to com mitment,1 and important duties of the state, including the police function of protecting society and the parens patriae function of protecting the individual.2 Today, in voluntary civil commitment is usually the last resort of family members, law enforcement officers, mental health and social services professionals, and judicial officers for providing treatment and care to individuals who are either unwilling or unable to receive such service voluntarily. Few exercises of governmental authority engender as much controversy and debate as involuntary civil commitment. | 1 |  |
| Database search | Anger rumination and imagined violence as related to violent behavior before and after psychiatric hospitalization | Hardin, K. M. : C., I. M. :. Kosiak, K. :. Novaco, R. W. (2022). Anger rumination and imagined violence as related to violent behavior before and after psychiatric hospitalization. Journal of Clinical Psychology. https://doi.org/10/gqs8nm | 2022 | research article | Approximately 40% of the patients were involuntarily committed (i.e., a court or physician found the person to be of danger to themselves or others or unable to meet their basic needs due to mental illness, and thus were detained in emergency psychiatric hospitalization for evaluation and treatment) | 1 |  |
| Database search | Community-based forensic treatment | Heilbrun, K. : G., Patricia A. (1998). Community-based forensic treatment. In Treatment of offenders with mental disorders. (1998-07168-004; pp. 168–210). The Guilford Press. https://search.ebscohost.com/login.aspx?direct=true&db=psyh&AN=1998-07168-004&site=ehost-live | 1998 | Book excerpt | CBFT programs treating individuals in this category may be either outpatient clinics or residential ("halfway house") facilities. The clinics tended to offer both assessment and treatment services to individuals in a variety of legal categories including pretrial, parole, probation, and sometimes insanity acquittee. Certain clinics offering substance abuse treatment also accepted self-referred (voluntary) clients, underscoring the heterogeneity of the client population for such programs. | 1 |  |
| Database search | Right to refuse psychotropic medication: an interdisciplinary proposal | Hickman, F. J. : R., Phillip J. :. Olson, Kathryn B. (1982). Right to refuse psychotropic medication: An interdisciplinary proposal. *Mental Disability Law Reporter*, *6*(2), 122–130. | 1982 | editorials, commentaries, and letters | 2. An "involuntary patient" is a patient who has been admitted to a hospital under one of the following provisions: a. emergency medical certificate; b. judicial certificate; c. after a finding of not guilty by reason of insanity; d. after a finding of incompetency to stand trial; e. penal transfers. | 1 |  |
| Database search | Does your patient have the right to refuse medications? A brief guide to the legal aspects of administering involuntary medications | Hicks, C. : W., S. (2020). Does your patient have the right to refuse medications? A brief guide to the legal aspects of administering involuntary medications. *Curr. Psychiatry*, *19*(4), 22–30. | 2020 | Educational materials | Involuntary medications vs emergency medications: Administering medications despite a patient's objection differs from situations in which medications are provided during a psychiatric emergency. In an emergency, courts do not have time to weigh in. Instead, emergency medications (most often given as IM injections) are administered based on the physician's clinical judgment. The criteria for psychiatric emergencies are delineated at the state level, but typically are defined as when a person with a mental illness creates an imminent risk of harm to self or others. Alternative approaches to resolving the emergency may include verbal deescalation, quiet time in a room devoid of stimuli, locked seclusion, or physical restraints. These measures are often exhausted before emergency medications are administered. | 1 |  |
| Database search | Patient perceptions of coercion in mental hospital admission | Hiday, V. A. : S., M. S. :. Swanson, J. :. Wagner, H. R. (1997). Patient perceptions of coercion in mental hospital admission. International Journal of Law and Psychiatry, 20(2), 227–241. https://doi.org/10/czjhz6 | 1997 | research article | Although subjugation to the state's coercive power legally distinguishes involuntary from voluntary patients, coerced hospitalization or treatment may occur in the absence of legal involuntary status. | 1 |  |
| Database search | A prospective, multicenter study of patients' refusal of antipsychotic medication | Hoge, S. K. : A., P. S. :. Lawlor, T. :. Beck, J. C. :. Litman, R. :. Greer, A. :. Gutheil, T. G. :. Kaplan, E. (1990). A prospective, multicenter study of patients’ refusal of antipsychotic medication. *Archives of General Psychiatry*, *47*(10), 949–956. | 1990 | research article | Voluntary Reacceptance. Fifty percent (49/98) of completed refusal episodes ended with patients taking medication voluntarily, and in an additional 4% (4) patients voluntarily reaccepted neuroleptics after receiving involuntary medication to control emergency situations (chemical restraints). Thus, a majority of refusal episodes (55%)were resolved with voluntary reacceptance. | 1 |  |
| Database search | Expedited Diversion of Criminal Defendants to Court-Ordered Treatment | Hoge, S. K. : B., R. J. (2021). Expedited Diversion of Criminal Defendants to Court-Ordered Treatment. The Journal of the American Academy of Psychiatry and the Law, 49(4), 517–525. https://doi.org/10.29158/JAAPL.210076-21 | 2021 | review article | In summary, they are specialty criminal courts that operate under existing prosecutorial and judicial authority, typically without specific governing statutes. Each local mental health court must be designed, planned, funded, and staffed by the agreement of various stakeholders, including the judiciary, prosecutors, defense bar, and community providers. In addition, the stakeholders must agree on defendants" clinical and legal eligibility criteria. Defendants" participation in mental health courts is voluntary. Therefore, defendants must be competent to stand trial and agree to the diversion to mental health court. The legal process either involves a conditional guilty plea or, in some courts, a preadjudication suspension of proceedings for the duration of court-supervised treatment with the expectation that the charges will be dropped upon successful completion. Defendants must be functioning sufficiently well to be placed in the community and receive outpatient services. As the cited figures indicate, diversion programs have not been broadly implemented, and these innovations have had little aggregate national impact on the increasing flow of mentally disordered individuals entering the criminal justice system. Clearly there is room for new approaches. | 1 |  |
| Database search | Perceptions of coercion in the admission of voluntary and involuntary psychiatric patients | Hoge, S. K. : L., C. W. :. Eisenberg, M. :. Gardner, W. :. Monahan, J. :. Mulvey, E. :. Roth, L. :. Bennett, N. (1997). Perceptions of coercion in the admission of voluntary and involuntary psychiatric patients. International Journal of Law and Psychiatry, 20(2), 167–181. https://doi.org/10/bkg3q9 | 1997 | research article | Perhaps the best-known criticisms of admission practices have focused on the coercion of individuals to sign themselves into the hospital as voluntary patients ("coerced voluntaries"). ... Conversely, it has been reported that some patients admitted under involuntary hospitalization procedures may actually seek hospitalization ("uncoerced involuntaries"). Anecdotal reports suggest that patients may contrive to arrange their own commitment in order to overcome obstacles to voluntary admission in mental health systems (Miller, 1980). Similarly, impediments to voluntary admission may lead admitting clinicians to employ involuntary mechanisms to admit willing patients. | 2 |  |
| Database search | Involuntary Processes: Knowledge Base of Health Care Professionals in a Tertiary Medical Center in Upstate South Carolina | Holder, S. M. : W., C. :. Rogers, K. :. Griffeth, B. :. Peterson, E. :. Blackhurst, D. :. Ochonma, C. (2018). Involuntary Processes: Knowledge Base of Health Care Professionals in a Tertiary Medical Center in Upstate South Carolina. Community Mental Health Journal, 54(2), 149–157. https://doi.org/10/gcx98j | 2018 | research article | Involuntary hospitalization is a unique medico-legal process through which a person who presents a danger to oneself or others can be hospitalized for stabilization and treatment without his express permission to do so. This process has been evolving in the United States since the early eighteenth century (Anfang and Appelbaum 2006; Appelbaum 1997; Testa and West 2010). The current process for the involuntary hospitalization of persons with mental illness has provided a legal means by which loved ones, caregivers and healthcare professionals (and others) can intervene when a person with a mental illness becomes so impaired that the said person represents a danger to self and/or others (Anfang and Appelbaum 2006; Appelbaum 1997; Johnson and St) | 1 |  |
| Database search | Self-reported symptoms and treatment outcome among non-offending involuntary inpatients | Hopko, D. R. : A., P. M. :. Cowan, K. :. Shah, N. (2002). Self-reported symptoms and treatment outcome among non-offending involuntary inpatients. Journal of Forensic Psychiatry, 13(1), 88–106. https://doi.org/10/ddqtxn | 2002 | research article | In the present context, the term "involuntary legal status" refers to those patients admitted without consent, according to the statutes of Texas state law. The term is used in a general manner to encompass all non-consenting adults, regardless of the specific precipitating factors of their admission and with attention to the fact that the sample being studied is primarily a non offending or non-criminal group. It also is important to note that the concept of involuntary commitment is not meant to imply that all patients admitted under such conditions are necessarily coerced. In fact, data have emerged that suggest legal status is only a blunt index of whether a patient experienced coercion during inpatient admission. In reality, a minority of legally voluntary inpatients report being coerced into hospitalization while a significant number of legally involuntary inpatients indicate that they "freely" chose to be admitted. (Monahan, Hoge, Lidz et al., 1995; Monahan, Lidz, Hoge et al., 1999). Further adding to the equation, alternative factors such as poverty, lack of insight and more severe mental illness may independently or concurrently (with coercion) play a significant role in the process of involuntary commitment. Finally, regardless of whether the use of coercion is evident during involuntary admission, involuntary status should not be equated with an unchanging attitude toward hospitalization or an unwillingness to be treated. To the contrary, there are data to suggest that patients" views concerning the need for hospitalization as well as their receptivity of treatment can change over time (Monahan, Lidz, Hoge et al., 1999; Spence, Goldney and Costain, 1988). | 1 | Texas state law (no specific citation) |
| Database search | The Sell decision: Implications for psychological assessment and treatment | Hunter, R. H. : R., A. J. :. Spaulding, W. D. (2005). The Sell decision: Implications for psychological assessment and treatment. Prof. Psychol. Res. Pract., 36(5), 467–475. https://doi.org/10/dxkb85 | 2005 | Clinical guidelines | This makes irrelevant the question of involuntary medication for the sole purpose of restoring competence to stand trial. Involuntary treatment (using medications against the person's will) would proceed if the person were determined dangerous or otherwise incompetent to make decisions concerning his or her own behalf. | 1 |  |
| Database search | Legal, ethical and clinical implications of prescribing involuntary, life-threatening treatment: The case of the sunshine kid | Irvin, T. L. (2003). Legal, ethical and clinical implications of prescribing involuntary, life-threatening treatment: The case of the sunshine kid. Journal of Forensic Sciences, 48(4), 856–860. https://doi.org/10/gqs8pc | 2003 | case report | Involuntary treatment is a familiar concept to psychiatrists. In clinical practice, this usually involves the hospitalization and pharmacological management of patients with severe mental disorders. The scope of involuntary treatment is not limited to the management of mental illness alone. Psychiatric patients are commonly afflicted with medical illnesses as well and may require hospitalization and invasive procedures for optimal management of these disorders. In many instances, these patients lack the capacity to consent to or refuse medical treatment (1,2). In the state of New York, patients who are found to lack the capacity to decide their treatment may have a guardian, conservator, or committee consent to treatment on their behalf. | 1 |  |
| Database search | Involuntary hospitalization of primary care patients | Johnson, J. M. : S., T. A. (2014). Involuntary hospitalization of primary care patients. Primary Care Companion to the Journal of Clinical Psychiatry, 16(3). https://doi.org/10/gqs8pg | 2014 | review article | WHAT IS INVOLUNTARY COMMITMENT? Involuntary commitment, often referred to as civil commitment, represents the legal process of hospitalizing a person against his or her stated wishes (1). State governments, acting under 2 major legal principles, have enacted laws to guide the process of involuntary commitment of patients with psychiatric illness. These 2 legal principles are parens patriae, or the responsibility of the state to intervene on behalf of its citizens who cannot act in their own best interest (2,3) and "police power," or the necessity of the state to broadly protect the interests of its citizens (2). The parens patriae component of government has traditionally been viewed as the basis for the "need for treatment of patients in commitment, and it fueled most commitments prior to the 1960s, when patients were typically involuntary hospitalized on the basis of their need to be treated, irrespective of the risk to oneself or others. The police power component of government, on the other hand, has been viewed as providing the "dangerousness" criteria for commitment (2,3). | 1 | 1. Schouten R. Civil commitment and the patient refusing treatment. In: Stern TA, Herman JB, Slavin PL, editors. Massachusetts General Hospital Guide to Primary Care Psychiatry. 2nd ed. New York, NY: McGraw-Hill Companies; 2004. pp. 577‚Äì587. [Google Scholar] |
| Database search | Involuntary medication of patients who are incompetent to stand trial: A review of empirical studies | Ladds, B. : C., A. (1994). Involuntary medication of patients who are incompetent to stand trial: A review of empirical studies. *Bulletin of the American Academy of Psychiatry and the Law*, *22*(4), 519–532. | 1994 | review article | "Rodenhauser el al. define "refuser" as any patient who persists in nonacceptance of medication for one week, or less if a patient is considered dangerous. In addition, "involuntary treatment" is defined as the medication given to any patient who refused medication and who "posed an imminent serious physical threat to themselves or others." Thus, these studies included instances in which medication was administered involuntarily on a temporary emergency basis." | 1 | Rodenhauser P, et al: Relationships between legal and clinical factors among forensic hospital patients. Bull Am Acad Psychiatry Law l6:32 1-32. 1988 |
| Database search | Civil commitment and involuntary hospitalization of the mentally ill | Lareau, C. R. (2013). Civil commitment and involuntary hospitalization of the mentally ill. In Handbook of psychology: Forensic psychology., Vol. 11, 2nd ed. (2012-28467-014; pp. 308–331). John Wiley & Sons, Inc. https://search.ebscohost.com/login.aspx?direct=true&db=psyh&AN=2012-28467-014&site=ehost-live | 2013 | Book excerpt | Civil commitment is commonly known as involuntary civil commitment. For purposes of this chapter, the term civil commitment is used throughout because all commitment is by definition involuntary. ... Civil commitment refers to the process and procedure by which the state mandates hospitalization for persons with mental illness who require incapacitation, treatment, or care due to concerns of self-harm, or harm to others. | 1 |  |
| Database search | Incidences of involuntary psychiatric detentions in 25 U.S. States | Lee, G. : C., D. (2021). Incidences of involuntary psychiatric detentions in 25 U.S. States. Psychiatric Services, 72(1), 61–68. https://doi.org/10/gqcn56 | 2021 | research article | Involuntary inpatient civil commitment refers to a legal intervention used in every U.S. state where individuals not accused of a crime but suspected by laypersons or after a psychiatric evaluation to pose a danger to themselves or others because of mental illness or substance abuse can be seized, transported, or held in custody at a hospital or other authorized facility for examination and sometimes involuntary treatment. Civil commitment raises vexing clinical, ethical, legal, and human rights issues (1). However, how often it is employed in the United States is unknown. No federal data set appears to track commitments (2), unlike rates of other formal deprivations of liberty, such as criminal arrest or imprisonment, which are documented yearly by the federal government (3, 4). | 1 |  |
| Database search | Developing trends in prisoners' rights to mental health treatment | Leibman, F. H. : L., Neil. (1991). Developing trends in prisoners’ rights to mental health treatment. *American Journal of Forensic Psychology*, *9*(1), 19–28. | 1991 | review article | Basically, the developing law granting prisoners rights to mental health treatment evolved out of case law in two-areas: the right of involuntarily (civilly) committed patients in mental hospitals and the rights of prisoners to medical care. | 1 |  |
| Database search | Involuntary psychiatric hospitalization and social control | Leifer, R. (1966). Involuntary psychiatric hospitalization and social control. *The International Journal of Social Psychiatry*, *13*(1), 53–58. | 1966 | review article | The identification of psychiatry with medicine tends to distract from some important differences between them, particularly with respect to the practice of involuntary hospitalization: medical patients are only rarely, if ever, hospitalized without their consent, while psychiatric patients often are. Since the practice of involuntary institutionalization is more closely associated with the function of social control than with the function of medical treatment, it would seem to require some other justification than that provided by the medical model | 1 |  |
| Database search | Psychiatry, language and freedom | Leifer, R. (1982). Psychiatry, language and freedom. Metamedicine, 3(3), 397–416. https://doi.org/10/fph3m4 | 1982 | review article | My views on psychiatry and freedom may be briefly and succinctly stated in the form of a thesis and a corollary. The thesis is that one of the major social functions of psychiatry is to provide a paralegal system of social control. The foundation of this system is involuntary mental hospitalization by which certain individuals are deprived of freedom against their will and confined in psychiatric institutions. For political reasons, the social control functions of psychiatry are not recognized as such. They are disguised by the medical model of psychiatry, which ideologically represents the political, moral and religious functions of psychiatry as benevolent medical treatment. The essential feature of the medical model of psychiatry is that it uses the mechanical metaphors of the physical sciences together with the language and social context of medicine to define and explain not only the behavior of psychiatric patients, but also of their psychiatric physicians. | 1 |  |
| Database search | Involuntary psychiatric hospitalization | Leifer, R. (2018). Involuntary psychiatric hospitalization. Ethical Human Psychology and Psychiatry, 19(3), 182–184. https://doi.org/10/gqs8qr | 2018 | editorials, commentaries, and letters | More disturbingly, it is next to impossible to actually distinguish between the involuntary and voluntary status of a patient. This is because institutional psychiatrists typically ask admitted patients to sign in voluntarily, but if they refuse, they are threatened with being involuntarily committed. So if they agree to sign in voluntarily, they are classified as such, even though in actuality they were forced. This conceals the widespread repression and lack of true informed consent that occurs during the psychiatric admission process. A patient admitted by this fraudulent practice is facetiously called "an involuntary voluntary." | 1 |  |
| Database search | Ethics in psychiatry | Leong, G. B. : E., S. (1991). Ethics in psychiatry. Current Opinion in Psychiatry, 4(6), 872–875. https://doi.org/10/dctgjz | 1991 | review article | Involuntary hospitalization (civil commitment) is perhaps the cardinal ethical issue that arises in psychiatric prac tice. In philosophical terms, the moral difficulty associ ated with involuntary commitment reflects the potential conflict between two important values - the traditional duty of physicians to heal the sick (beneficence) versus the premium Western societies place on personal freedom (autonomy). As long as the patient and psychiatrist agree on a course of hospital treatment, consent is freely given and the conflict is avoided. However, should the patient refuse the required inpatient care, then the battle over voluntary hospitalization is joined. | 1 |  |
| Database search | Compulsory treatment for drug abuse | Leukefeld, C. G. : T., F. M. (1990). Compulsory treatment for drug abuse. International Journal of the Addictions, 25(6), 621–640. https://doi.org/10/cz2x5r | 1990 | editorials, commentaries, and letters | Nonvoluntary Treatment Admission=treatment while on probation or parole, awaiting prosecution, in prisoner status, or under civil commitment. | 1 |  |
| Database search | Commitment: the consistency of clinicians and the use of legal standards | Lidz, C. W. : M., E. P. :. Appelbaum, P. S. :. Cleveland, S. (1989). Commitment: The consistency of clinicians and the use of legal standards. *The American Journal of Psychiatry*, *146*(2), 176–181. | 1989 | research article | Involuntary commitment is a legal institution designed to facilitate psychiatric treatment. As such, it involves a unique power in our society to deprive an individual of liberty. For that reason, it is legally restricted in a variety of ways. Because history has shown that such powers are granted only tenuously, the mental health professions have a major interest in seeing that the power is not abused. | 1 |  |
| Database search | Sources of coercive behaviours in psychiatric admissions | Lidz, C. W. : M., E. P. :. Hoge, S. K. :. Kirsch, B. L. :. Monahan, J. :. Bennett, N. S. :. Eisenberg, M. :. Gardner, W. :. Roth, L. H. (2000). Sources of coercive behaviours in psychiatric admissions. Acta Psychiatrica Scandinavica, 101(1), 73–79. https://doi.org/10/cq2r4j | 2000 | research article | In table 2 titled 'Pressure and pressure types': Legal force definition: Using the authority of the law to effect the admission, i.e. involuntary commitment | 1 |  |
| Database search | Psychiatric involuntary commitment: A brief critique of modern day policy and practice | Lozovatsky, M. (2016). Psychiatric involuntary commitment: A brief critique of modern day policy and practice. Ethics in Biology, Engineering and Medicine, 7(1–2), 43–63. https://doi.org/10/gqs8q7 | 2016 | editorials, commentaries, and letters | To initiate an involuntary psychiatric commitment a state must temporarily and perhaps indefinitely suspend a person’s right to liberty and self-determination. ... A voluntary admission for treatment occurs at the request of the admittee (18 years of age or older) and with formal documentation of the applicants admission, or if a person is younger than 18 years of age, at the behest of their custodian, legal guardian, or parent, who can submit an admission application on the admittees behalf. These admittees are permitted to leave at any time following their submission of a written request for discharge, or, if the individual is under 18 years of age, after the submission of a request for discharge from the original petitioner, an individual with closer or equal relationship to the original petitioner, or the psychiatric centers legal department (i.e., Mental Hygiene Legal Services). Unless the director of the psychiatric center believes the patient meets criteria for an involuntary admission, which requires the approval of a judge within 72 hours of the written request for discharge, these individuals are released from their hospital commitment. | 2 |  |
| Database search | Who are the 'committed'? Update | Mahler, H. : C. J., B. T. (1984). Who are the “committed”? Update. *Journal of Nervous and Mental Disease*, *172*(4), 189–196. | 1984 | research article | "Involuntary" represents the number of hospitalizations in which the patient was civilly committed at least once at admission or during the hospitalization | 1 |  |
| Database search | Involuntary patient length-of-stay at a suburban emergency department | Maniaci, M. J. : L., C. :. Vadeboncoeur, T. F. :. Hodge, D. O. :. Dawson, N. L. :. Rummans, T. A. :. Roy, A. :. Burton, M. C. (2020). Involuntary patient length-of-stay at a suburban emergency department. American Journal of Emergency Medicine, 38(3), 534–538. https://doi.org/10/gqs8rg | 2020 | research article | An involuntary hold is a legal directive that provides for emergency services and temporary detention for mental health evaluation and treatment. These patients usually require a variety of interventions which may increase LOS. What is known regarding involuntary patients and LOS comes from studies of patients seen at urban academic medical centers where hospitalization need, patient suicidality, benzodiazepine and antipsychotic medication administration, and the timing of the involuntary hold were associated with increased LOS [5-7]. | 1 |  |
| Database search | Efficacy of coercion in substance abuse treatment | Marlowe, D. B. : G., David J. :. Merikle, Elizabeth P. :. Festinger, David S. :. DeMatteo, David S. :. Marczyk, Geoffrey R. :. Platt, Jerome J. (2001). Efficacy of coercion in substance abuse treatment. In Relapse and recovery in addictions. (2001-01126-008; pp. 208–227). Yale University Press. https://search.ebscohost.com/login.aspx?direct=true&db=psyh&AN=2001-01126-008&site=ehost-live | 2001 | research article | Coercion in substance abuse treatment is commonly viewed as being synonymous with a legal mandate to receive services. Clients who are involuntarily committed to treatment or referred to treatment by criminal justice authorities are typically defined as "coerced," while the remaining clients are defined as"voluntary." This has had several untoward consequences for measuring the effects of coercion on treatment outcome. | 2 |  |
| Database search | Forensic psychiatry - Civil law | Martone, C. A. : M., Cameron L. :. Singh, Amarpreet :. Hira-Brar, Shabneet :. Paul, Robindra :. Strassnig, Martin. (2008). Forensic psychiatry—Civil law. In Oxford American handbook of psychiatry. (2008-13270-017; pp. 807–885). Oxford University Press. https://search.ebscohost.com/login.aspx?direct=true&db=psyh&AN=2008-13270-017&site=ehost-live | 2008 | Book excerpt | "Types of civil commitments -Voluntary hospitalization. - Involuntary hospitalization. -Involuntary outpatient treatment."  "Voluntary hospitalization A voluntary patient, as an individual, retains the right to decide to accept or to reject treatment, including medications and ECT, and retains the (modified) right to leave the hospital. Essentially two types of voluntary admissions exist: Informal admission - Patient must be discharged immediately upon his/her request. -No statutory period during which he/she can be held in the hospital following a request for discharge. -Patient may leave the institution, often in the midst of treatment and against the advice of physicians who may not prevent it, assuming involuntary commitment is not justified. Formal admission -Includes a mandatory period (3-5 days), defined by statute, during which the hospital has the discretion to hold the patient against his/her wishes should they attempt to leave. -Most states require patients to give informed consent for voluntary hospitalization and written notice of a desire to leave against medical advice (AMA). -In the case of seriously mentally ill patients, where continued hospitalization is necessary t o protect the patient or others, this provides time for the patient's family or the institution to seek an involuntary commitment from the court." + Intents of voluntary hospitalization: - To promote patient autonomy. -To promote a collaborative relationship between physician and patient.   Involuntary hospitalization: Legal bases -Parens patriae: The state takes responsibility for those unable to care for themselves. -Police power: The state has authority to prevent harm to the community, including harm to mentally il persons themselves. | 2 |  |
| Database search | Commitment to assessment and treatment: Comprehensive care for patients gravely disabled by alcohol use disorders | McCormack, R. P. : W., A. R. :. Goldfrank, L. R. :. Caplan, A. L. :. Ross, S. :. Rotrosen, J. (2013). Commitment to assessment and treatment: Comprehensive care for patients gravely disabled by alcohol use disorders. The Lancet, 382(9896), 995–997. https://doi.org/10/f2mg3s | 2013 | review article | For some patients, this approach might only be possible with involuntary commitment (ie, therapeutic detention). | 1 |  |
| Database search | Clinical guidelines for involuntary outpatient treatment | Meloy, J. R. : H., Ansar :. Schiller, Eugene F. (1990). Clinical guidelines for involuntary outpatient treatment. In Practitioner’s resource series (1990-97292-000). Professional Resource Exchange, Inc. https://search.ebscohost.com/login.aspx?direct=true&db=psyh&AN=1990-97292-000&site=ehost-live | 1990 | Clinical guidelines | involuntary outpatient commitment has various meanings, and we would like to define our terms before proceeding. For our purposes, involuntary outpatient commitment is the legal commit ment of a mentally disordered person to outpatient psychi atric treatment. It includes, but is not limited to, condition al release, which is involuntary outpatient commitment that follows commitment to an inpatient setting. From our perspective, JOC may be the least restrictive alternative necessary for treating the patient, and inpatient hospitalization may never be needed. | 1 |  |
| Database search | The US Supreme Court looks at voluntariness and consent | Miller, R. D. : F., P. B. (1984). Outpatient commitment: Treatment in the least restrictive environment? *Hospital & Community Psychiatry*, *35*(2), 147–151. | 1984 | review article | Second, predeprivation remedies were easily implemented in this situation, since Florida already had an established procedure for those who were unwilling to consent to hospitalization (i.e., the involuntary admission process). All that need be done was to extend that protection to those who are unable to consent. Third, the state could not claim that the actions of its employees were unauthorized in the same way in which the guards" conduct was in Parratt and Hudson, since the statutory scheme explicitly delegated the authority to hospital staff to affect the very deprivation of which Burch complained. The statute also delegated the duty to initiate safeguards to prevent unlawful admissions (e.g.,"voluntary" admission of patients incompetent to consent). It was the breach of that duty which created the rights violation recognized by the Court"s majority. | 2 |  |
| Database search | Voluntary "involuntary" commitment--the briar-patch syndrome | Miller, R. D. (1980). Voluntary “involuntary” commitment—The briar-patch syndrome. *The Bulletin of the American Academy of Psychiatry and the Law*, *8*(3), 305–312. | 1980 | review article | involuntarily committed patients are by definition unwilling to seek or accept hospitalization has been accepted as axiomatic, and unchallenged | 1 |  |
| Database search | Outpatient commitment: treatment in the least restrictive environment? | Miller, R. D. (1994). The US Supreme Court looks at voluntariness and consent. International Journal of Law and Psychiatry, 17(3), 239–252. https://doi.org/10/d66krv | 1994 | review article | North Carolina statutes provide for only two types of admissions to mental hospitals: voluntary (initiated by the patient, who can leave within 72 hours of requesting discharge) and involuntary (initiated by any adult). The involuntary process (36) involves concurrence by a judicial official, such as a clerk of the court or a magistrate, and, except in emergency situations (37), examination by a community based physician. Examination by the admitting physician at the receiving hospital is also required. | 2 | North Carolina General Statutes, section 122-58.3 through section 12258.6; North Carolina General Statutes, section 122-58.18 |
| Database search | Legal issues in psychiatric treatment | Mills, M. J. (1984). Legal issues in psychiatric treatment. *Psychiatric Medicine*, *2*(3), 245–261. | 1984 | Book excerpt | Components of Consent- Most commentators agree that the requisites of informed consent are competence, knowledge, and voluntariness. These standards, first explicitly discussed in the Nuremberg Trials, were applieed to the topic of informed consent in the Kaimowitz case. They note that, for informed consent to occur, the patient must have the abiliyty to weigh the physician"s information (intelligence and judgment), has to have knowledge about the treatment (the risks, benefits, and alternatives), and has to be voluntarily situated (acting without improper inducement or coercion) | 1 |  |
| Database search | Prevalence of involuntary commitment for alcohol dependence | Mindock, S. : W., K. :. Fleming, M. F. (2012). Prevalence of involuntary commitment for alcohol dependence. *Wisc. Med. J.*, *111*(2), 55–57. | 2012 | research article | The aim of this article is to provide a statewide snapshot of involuntary commitment (IC) for alcohol dependence. In 1968 congress passed the Alcoholic Rehabilitation Act of 1968 (Public Law 90-574), which was the first federal law to address the need for alcoholism to be treated as a health problem vs a criminal problem. By 1971 this legislation was expanded to The Uniform Alcoholism and Intoxication Treatment Act, which allowed states to provide the health and legal guidelines to treat alcoholism.1 Following this federal act, the state of Wisconsin enacted legislation that addressed the issue of involuntary commitment for alcoholism. This state statute defines an involuntary alcohol commitment as "a civil, legal proceeding which allows for an alcohol or drug dependent individual who is dangerous as a result of that use, to be placed in a treatment setting against his/her will."2 Other states have enacted similar statutes. | 1 | Wisconsin state law: . Wis Stat ¬ß 51.45. https://docs.legis.wisconsin.gov/statutes/statutes/51/45. Accessed March 5, 2012. |
| Database search | Integrated treatment for dual disorders: A guide to effective practice | Mueser, K. T. : N., Douglas L. :. Drake, Robert E. :. Fox, Lindy :. Barlow, David H. (2003). Integrated treatment for dual disorders: A guide to effective practice. In Treatment manuals for practitioners (2003-07130-000). The Guilford Press. https://search.ebscohost.com/login.aspx?direct=true&db=psyh&AN=2003-07130-000&site=ehost-live | 2003 | Book excerpt | (Ch 17, Page 249)  In this chapter, we discuss the use of involuntary and coerced interventions in the treatment of clients with dual disorders. Involuntary interventions can be defined as strategies that involve the physical or legal restriction of clients, their property, or their authority to make decisions. Civil commitment to a psychiatric hospital, payeeship, and guardianship are all commonly used involuntary interventions. Coerced interventions are strategies used to engage people in mental health or substance abuse treatment by threatening the loss of personal choice and freedom if individuals do not comply with treatment recommendations. For example, an individual with dual disorders who is convicted of a crime may be mandated by the criminal justice system to receive treatment for his or her disorders, or someone with multiple psychiatric hospitalizations and admissions to inpatient detoxification units may be civilly committed to outpatient treatment. In either case, the failure to receive the appropriate outpatient treatment can result in imprisonment or involuntary hospitalization. | 1 |  |
| Database search | Neuroprediction, violence, and the law: Setting the stage | Nadelhoffer, T. : B., S. :. Grafton, S. :. Kiehl, K. A. :. Mansfield, A. :. Sinnott-Armstrong, W. :. Gazzaniga, M. (2012). Neuroprediction, violence, and the law: Setting the stage. Neuroethics, 5(1), 67–99. https://doi.org/10/fmtr82 | 2012 | review article | involuntary civil commitment - i.e., "the state-sanctioned involuntary hospitalization of mentally disordered individuals who are thought to need treatment, care, or incapacitation because of self-harming or antisocial tendencies" ([12], p. 297). | 1 | Melton, G., J. Petrila, N. Poythress, and C. Slobogin. 1997. Psychological evaluations for the courts: A handbook for mental health professionals and lawyers,2nded.New York: Guilford. |
| Database search | Civil commitment and involuntary outpatient commitment | Nakic, M. (2017). Civil commitment and involuntary outpatient commitment. In *Psychiatry and the Law: Basic Principles* (pp. 63–73). Springer International Publishing. | 2017 | Book excerpt | (PAGE 65) In order to ensure safety and administer necessary treatment, psychiatrists must resort to involuntary or civil commitment, a legal process through which an individual with symptoms of severe mental illness is court-ordered into psychiatric treatment. Involuntary commitment decisions present a unique challenge for psychiatrists [2, 3]. Psychiatric interventions are guided by four ethical principles of medical practice; non-maleficence, autonomy, beneficence, and justice. Physicians are expected to refrain from causing harm (non-maleficence) and obliged to provide treatments that are in the best interest of their patients (beneficence). The principle of autonomy recognizes the right of an individual to selfdetermination and requires that a physician respects the authority of a patient to make their own medical decisions, even when these decisions appear to be unwise. Justice dictates that medical benefits should be dispensed fairly. Intuitively, these principles seem to be of clear value and application. However, challenges arise when principles of autonomy and beneficence are in conflict, such as when considering involuntary commitment [4–6]. Clinical decisions related to depriving patients of their civil liberties carry tremendous responsibility, and thus psychiatrists rely on state law and hospital regulations to guide their decision-making process. Contemporary involuntary commitment statutes are the product of over two centuries of work to effect a balance between individual liberty rights and concerns for patients’ best interests. | 1 |  |
| Cited by another source | TREND IN PSYCHIATRIC INPATIENT CAPACITY, UNITED STATES AND EACH STATE, 1970 TO 2014 | NASMHPD. (2017). TREND IN PSYCHIATRIC INPATIENT CAPACITY, UNITED STATES AND EACH STATE, 1970 TO 2014. National Association of State Mental Health Program Directors. https://www.nasmhpd.org/sites/default/files/TACPaper.2.Psychiatric-Inpatient-Capacity_508C.pdf | 2017 | Technical report | Psychiatric patients may enter inpatient and other 24-hour residential treatment facilities either on a voluntary basis or involuntarily, under their state’s civil commitment statute, if they are determined by a court to be at risk to harming themselves or others as a result of the symptoms of their mental illness. There are two types of involuntary admissions that are tracked in the SAMHSA datasets: “involuntary, non-forensic” (persons civilly committed) and “involuntary, forensic” (individuals ordered by a criminal court to evaluation or treatment). | 1 |  |
| Database search | Psychotic denial of pregnancy: legal and treatment considerations for clinicians | Nau, M. : B., H. E. :. Street, J. (2011). Psychotic denial of pregnancy: Legal and treatment considerations for clinicians. *The Journal of the American Academy of Psychiatry and the Law*, *39*(1), 31–39. | 2011 | Educational materials | The first question with which we are faced in this case is whether there are grounds to hold the patient for psychiatric reasons - that is, what is the legal basis for involuntary commitment? | 1 |  |
| Database search | Involuntary interventions in dual disorders programs | Noordsy, D. L. : M., Carolyn C. :. Drake, Robert E. (2002). Involuntary interventions in dual disorders programs. In Ethics in community mental health care: Commonplace concerns. (2002-00412-007; pp. 95–115). Kluwer Academic/Plenum Publishers. https://search.ebscohost.com/login.aspx?direct=true&db=psyh&AN=2002-00412-007&site=ehost-live | 2002 | Book excerpt | In mental health care, involuntary interventions are legally mandated mechanisms for limiting a person's choices, and the specifics vary from state to state. The mechanisms fall into three categories— protections for person and property, mandates for treatment, and orders associated with illegal behaviors. Protections for person and property take the form of payeeships for Supplemental Security Income, Social Security Disability Insurance, and other funds, as well as guardianships or conservatorships over the person, the person's medical care, or the person's property. Mandates for treatment include orders for emergency inpatient or outpatient treatment, involuntary hospitalization, conditional discharge from the hospital, and inpatient or outpatient commitments. Orders associated with illegal behaviors, which can be tailored to address dual disorders treatment goals, encompass restraining orders, conditions for avoiding prosecution, detention, conditions of sentencing, and stipulations or conditions associated with probation or parole. | 1 |  |
| Database search | Involuntary civil commitment: Applying evolving policy and legal determination in community mental health | Nunley, W. : N., Bernadette :. Cutler, David L. :. Dentinger, Jean :. McFarland, Bentson. (2013). Involuntary civil commitment: Applying evolving policy and legal determination in community mental health. In Modern community mental health: An interdisciplinary approach. (2013-08779-004; pp. 49–61). Oxford University Press. https://search.ebscohost.com/login.aspx?direct=true&db=psyh&AN=2013-08779-004&site=ehost-live | 2013 | Book excerpt | Involuntary civil commitment is the legal procedure used by the courts to require mentally ill and dangerous individuals to accept psychiatric treatment. It is a process that is basic to all mental health systems, yet most mental health professionals know little about it. Its coercive nature is to a large extent what causes much of the avoidance and perhaps explains the lack of attention paid to it in academic course work. However, understanding this process is really quite important for anyone working in the field. Th e policies and procedures referred to here as "civil commitment" have significantly changed during the past forty years in the United States (Faulkner et al. 1985). | 1 |  |
| Hand search organization/agency/association website | Psychiatric – Mental Health Nursing Orientation Guidebook | ONS. (2018). Psychiatric – Mental Health Nursing Orientation Guidebook. Office of Nursing Services (ONS) Mental Health Field Advisory Committee - U.S. Department of Veteran’s Affairs. https://www.va.gov/covidtraining/docs/ONS_Psych_Mental_Health_Nursing_Orientation_Guide.pdf | 2018 | educational materials | (1) "If the refusal of care and/or treatment is deemed to pose a significant likelihood or imminent risk of harm to self or others, a patient may be committed as an involuntary patient."  (2) on page 34/39: “The P-MHN has a responsibility to be aware of legal and regulatory statutes relating to involuntary treatment. Involuntary treatment is guided by state law and individual facility policy. The guidelines and statutes required for an involuntary commitment vary from state to state. Please be familiar with your state policy and facility standard procedure for involuntary treatment/commitment.”  (3) There are definitions on page 107/112, under committment status: “Voluntary hospitalization allows the person to freely choose to discharge from the facility by utilizing the proper process. Involuntary hospitalization results in a period of commitment established in compliance with state legal guidelines. Involuntary commitment may also be referred to as “detained”, “on a hold”, or state variations regarding verbiage of such.” | 2 |  |
| Database search | Involuntary commitment and the right to refuse medication | Oriol, M. D. : O., R. Dale. (1986). Involuntary commitment and the right to refuse medication. Journal of Psychosocial Nursing and Mental Health Services, 24(11), 15–20. https://doi.org/10/gqs86j | 1986 | magazine style article | Although mental health laws vary from state to state, involuntary commitment is confinement of an unwilling individual to an institution for the mentally ill, because of displays of behavior considered dangerous to the self or others (Haber, 1982). Furthermore, an individual may be involuntarily hospitalized on a temporary basis for the purpose of observation and diagnosis. In either case, each state requires some standard of proof that the individual is dangerous or mentally ill. | 1 | Haber et al., 1982. J Haber, A Leach, S Schudy, B Sideleau. Comprehensive psychiatric nursing. (2nd edition), McGraw-Hill, New York (1982) |
| Database search | Involuntary use of interventions: pros and cons | Parrish, J. (1993). Involuntary use of interventions: Pros and cons. *Innovations & Research*, *2*(1), 15–22. | 1993 | review article | In the context of this discussion, I use the term "involuntary intervention" to refer to civil commitment to an inpatient facility and/or forced medication. | 1 |  |
| Database search | Revisiting the civil commitment/involuntary treatment stalemate using limited guardianship, substituted judgment and different due process considerations: A work in progress | Parry, J. W. : B., James C. (1990). Revisiting the civil commitment/involuntary treatment stalemate using limited guardianship, substituted judgment and different due process considerations: A work in progress. *Mental & Physical Disability Law Reporter*, *14*(2), 102–107. | 1990 | review article | Legally, a treatment decision, which is a voluntary, knowledgeable, and competent is informed and thus binding. "Voluntary" means the individual is not coerced into making the decision. | 1 |  |
| Cited by another source | Involuntary Civil Commitment in the 90s: A Constitutional Perspective | Parry, J. W. (1994). Involuntary civil commitment in the 90s: A constitutional perspective. *Mental & Physical Disability Law Reporter*, *18*(3), 320–336. | 1994 | review article | "Adults. A legal guardian or a parent with guardianship powers can con sent to have the ward/adult child civilly committed. Depending on the jurisdiction and circumstances, the commitment may either be "voluntary," whereby the guardian can withdraw consent and initiate a pro cess leading to discharge unless invol untary commitment proceedings are instituted, or "involuntary," whereby if consent is withdrawn some type of proceeding?either administrative or judicial?must be held before dis charge will be granted. Minors. A parent or legal guardian can consent, subject to administra tive review as established in Parham case (discussed further below), to have a child civilly committed. Like the commitment of an adult, the commitment of a minor can either be "voluntary" in that the parent's with drawal of consent will lead to dis charge or "involuntary" in that dis charge depends on an administrative or legal proceeding."  "Of all the civil commitment types discussed here, only one type - informal commitment - is really voluntary in the sense of allowing the patient self-determination. All the other types involve some level of coercion and/or substituted consent of another person. Thus, for most purposes, civil commitment should be viewed as a series of interventions involving differene degrees and types of involuntariness" | 3 |  |
| Database search | Norms, commitment, and psychotherapy with the involuntarily hospitalized psychiatric patient | Parsons, P. J. (1984). Norms, commitment, and psychotherapy with the involuntarily hospitalized psychiatric patient. Psychotherapy Patient, 1(2), 81–87. https://doi.org/10/cpznxk | 1984 | Book excerpt | In addition to the above-mentioned, commitment also refers to consignment to a mental institution. Individuals are committed to a psychiatric hospital in the United States for either one of two purposes. The first is protection of society or even of the individual because of his or her dangerousness. The second is treatment and care, determined by a psychiatrist to be in the client's best interest (parens patriae model). This commitment is most frequently involuntary-not under the client's own volition. Individuals committed to state psychiatric hospitals are a population who have traditionally "slipped through the cracks" or filtered through private practitioners, private psychiatric hospitals, general hospitals, community mental-health centers, county hospitals, and so forth to finally end up in a state psychiatric facility. | 1 |  |
| Database search | The historic antecedents to the current polemics on involuntary psychiatric hospitalization | Peszke, M. A. (1984). The historic antecedents to the current polemics on involuntary psychiatric hospitalization. Comprehensive Psychiatry, 25(2), 238–246. https://doi.org/10/d5c352 | 1984 | research article | Most of the current problems regarding the practice and the principle underlying the involuntary psychiatric hospitalization (or civil commitment of the mentally ill) have occurred in the past, some in the distant past. Psychiatry has many roots but in spite of many calls uttered in recent years for the return of psychiatry to the medical fold, it can be documented that psychiatry has never been as integrated with medicine as it is now. The historical antecedents of psychiatry are based on the institutional model of the asylum and on the social mandate to protect society from the unpredictable and irrational behavior of the mentally disordered; i.e., the exercise of the police power of the State. Thus, it is fair to refer to this practice as a public-health speciality. Psychiatry has been practiced under various names for centuries, in medieval days English bishops were empowered to license physicians to treat the melancholic and mad. | 1 |  |
| Database search | Treatment engagement, access to services, and civil commitment reform: Would these strategies help reduce firearm-related risks? | Pinals, D. A. (2016). Treatment engagement, access to services, and civil commitment reform: Would these strategies help reduce firearm-related risks? In Gun violence and mental illness. (2015-55277-011; pp. 291–315). American Psychiatric Association. https://search.ebscohost.com/login.aspx?direct=true&db=psyh&AN=2015-55277-011&site=ehost-live | 2016 | Book excerpt | Another exception to voluntary care is involuntary commitment. Involuntary civil commitment represents an order by a court to a form of care, usually but not always in an inpatient setting, as the vehicle to mandate into treatment specific individuals with mental illness. These individuals typically do not want mental health care but meet some (usually risk-related) criteria that justify society's authority to mandate such an involuntary approach. Although individuals can refuse medical hospitalization, involuntary psychiatric commitment laws allow and provide due process for requiring that an individual with mental illness be hospitalized or comply with outpatient services. Whether care provided under involuntary commitment statutes includes mandated medications varies across jurisdictions, although involuntary medication administration generally follows separate legal provisions and pathways. | 1 |  |
| Database search | Outcomes of patients in a low-intensity, short-duration involuntary outpatient commitment program | Pollack, D. A. : M., B. H. :. Mahler, J. M. :. Kovas, A. E. (2005). Outcomes of patients in a low-intensity, short-duration involuntary outpatient commitment program. Psychiatric Services, 56(7), 863–866. https://doi.org/10/cj99b2 | 2005 | research article | Involuntary outpatient commitment refers to court-ordered ambulatory treatment for persons with severe mental illness who are unlikely to adhere to care plans without external leverage (1). Typical implementations of involuntary outpatient commitment include its use as an alternative to hospitalization for patients who meet criteria for involuntary inpatient commitment (major psychiatric illness, with danger to self or others, or inability to meet basic needs) or as a step-down from inpatient treatment (conditional release) | 1 | 1. Torrey EF, Kaplan RJ: A national survey of the use of outpatient commitment. Psychiatric Services 46:778‚Äì784, 1995 |
| Database search | Retention and treatment issues on the psychiatric inpatient unit | Rachlin, S. (1994). Retention and treatment issues on the psychiatric inpatient unit. In Psychiatric-legal decision making by the mental health practitioner: The clinician as de facto magistrate. (1994-97201-004; pp. 77–99). John Wiley & Sons. https://search.ebscohost.com/login.aspx?direct=true&db=psyh&AN=1994-97201-004&site=ehost-live | 1994 | Book excerpt | A voluntary psychiatric patient, one who is virtually by definition competent, thus may not be treated over objection. An involuntary patient is presumed to be competent also, unless evidence exists to demonstrate otherwise. Only in this latter situation would involuntary treatment be possible | 1 |  |
| Database search | De-escalation in the emergency department | Richmond, J. S. (2021). De-escalation in the emergency department. In Behavioral emergencies for healthcare providers., 2nd ed. (2021-36863-021; pp. 221–229). Springer Nature Switzerland AG. https://search.ebscohost.com/login.aspx?direct=true&db=psyh&AN=2021-36863-021&site=ehost-live | 2021 | Book excerpt | Criteria for Psychiatric HospitalizationInvoluntary commitment, also known as civil commitment, is the legal process of hospitalizing a person against his or her stated wishes. The criteria for involuntary admission to a psychiatric unit are met when a patient is deemed to be at danger to themselves or others, or is unable to provide for himself or herself because of mental illness [2]. In the United States, forcing someone to enter a facility to undergo treatment is considered a significant curtailment of his or her rights. Two major legal principles have been used by lawmakers to protect individuals who are involuntarily admitted. | 1 |  |
| Database search | Regional differences in New York's assisted outpatient treatment program | Robbins, P. C. : K., K. J. :. Steadman, H. J. :. Swanson, J. W. :. Wilder, C. M. :. Swartz, M. S. (2010). Regional differences in New York’s assisted outpatient treatment program. Psychiatric Services, 61(10), 970–975. https://doi.org/10.1176/appi.ps.61.10.970 | 2010 | research article | Involuntary outpatient commitment statutes permit courts to mandate persons with severe mental illness to comply with treatment in the community. Failure to adhere to the conditions of the court order can result in a range of consequences, including involuntary hospitalization' | 1 |  |
| Database search | Anorexia: enigma and dilemma -- reflections on contemporary clinical interventions | Rosedale, M. : M., V. F. (1993). Anorexia: Enigma and dilemma—Reflections on contemporary clinical interventions. *Journal of the New York State Nurses Association*, *24*(4), 9–14. | 1993 | review article | It should be noted that this discussion is restricted to adult, voluntary, inpatients -- persons who presented for admission, requested, and consented to treatment of the illness. | 1 |  |
| Cited by another source | Civil commitment and the patient refusing treatment | Schouten, R. (2004). Civil commitment and the patient refusing treatment. In T. A. Stern, J. B. Herman, & P. L. Slavin (Eds.), *Massachusetts General Hospital Guide to Primary Care Psychiatry, 2nd ed* (pp. 577–587). McGraw Hill, New York, N.Y. | 2004 | Book excerpt | Individuals with serious mental illness may require hospitalization to protect themselves or others. One of the most difficult problems in this area is that symptoms of the illness may include a lack of insight into the need for hospitalization or treatment. The process of hospitalizing a person against his or her will is referred to as involuntary civil commitment. The law in this area can cause considerable confusion and frustration for the family and for the physician of a patient with serious mental illness. This frustration increases when caregivers realize that involuntary commitment in most states does not automatically mean that the patient can be forced to accept treatment. | 1 |  |
| Database search | Factors in the use of coercive retention in civil commitment evaluations in psychiatric emergency services | Segal, S. P. : L., T. A. :. Segal, M. J. (2001). Factors in the use of coercive retention in civil commitment evaluations in psychiatric emergency services. Psychiatric Services, 52(4), 514–520. https://doi.org/10.1176/appi.ps.52.4.514 | 2001 | research article | Coercive disposition. Patients who clearly expressed the wish to avoid hospitalization and who were detained were considered to have a coerced involuntary retention. | 1 |  |
| Database search | Once the wheels are in motion: Involuntary hospitalization and forced medicating | Seitler, B. (2008). Once the wheels are in motion: Involuntary hospitalization and forced medicating. Ethical Human Psychology and Psychiatry, 10(1), 31–42. https://doi.org/10.1891/1559-4343.10.1.31 | 2008 | review article | Involuntary hospitalization, formerly called civil commitment, is a term used to designate a legal procedure designed to compel an individual to receive inpatient treatment for a presumed emotional problem, all the while against his or her will. The general assumptions regarding the so-called necessity of imposing such an extreme and rightfully controversial course of action are 1. that such individuals are somehow dangerous to themselves, or to others, or that such individuals, because of their presumed problems, suffer from diminished capacity, and thus lack insight into and are unaware that they have any problems (Amador & David, 1998). | 1 |  |
| Database search | Predictors of aggression on the psychiatric inpatient service | Serper, M. R. : G., B. R. :. Herman, K. G. :. Richarme, D. :. Chou, J. :. Dill, C. A. :. Cancro, R. (2005). Predictors of aggression on the psychiatric inpatient service. Comprehensive Psychiatry, 46(2), 121–127. https://doi.org/10.1016/j.comppsych.2004.07.031 | 2005 | research article | Sixty-nine participants arrived at the hospital on their own accord (voluntarily), whereas 48 were brought in involuntary (against the patient"s will). Voluntary participants walked in or arrived freely, involuntary participants were brought in without their consent by police or emergency medical services. Arrival status information was unavailable for one participant.' | 2 |  |
| Database search | Concise guide to psychiatry and law for clinicians, 3rd ed | Simon, R. I. (2001). Concise guide to psychiatry and law for clinicians, 3rd ed. In Concise guides (2001-06677-000). American Psychiatric Association. https://search.ebscohost.com/login.aspx?direct=true&db=psyh&AN=2001-06677-000&site=ehost-live | 2001 | Book excerpt | The term civil commitment is not used in this book in its strict legal sense, which encompasses both voluntary and involuntary commit-ment. Psychiatrists do not usually think of a voluntary hospitalization as a commitment, even though patients who undergo conditional voluntary admission may be detained against their will for varying periods of time. When psychiatrists speak of involuntary hos-pitalization, they usually just say "commitment." In order not to confuse the reader, the terms voluntary and involuntary hospi-talization are used whenever possible. Civil commitment is used synonymously with involuntary hospitalization, except when referring to outpatient commitment. ... Hospitalization of patients is either voluntary or involuntary. Vol untary hospitalization consists of two types: informal and condi-tional. They are distinguished by the degree of freedom a patient is given in leaving the hospital. An informal voluntary admission permits a patient to leave at any time during the hospitalization, although minimal conditions may be attached (e.g., the discharge can only occur during a day shift, Monday through Friday). Only moral suasion can be used to induce the patient to stay in the hospital; no coercion is permissible. A conditional voluntary admission statutorily authorizes the hospital to detain a patient thought to be potentially dangerous to self or others. The patient may be held only for a specified number of days after the initial written notice of discharge is given in order to be evaluated for possible involuntary hospitali-zation. Civil commitment, or involuntary hospitalization. is the hospitalization of a person against his or her will for a statutorilv determined time based on the assessment that the person is mentally ill and a danger to self or others. | 3 |  |
| Database search | Outpatient commitment in mental health: is coercion the price of community services? | Sirica, C. (2000). Outpatient commitment in mental health: Is coercion the price of community services? *Issue Brief (George Washington University. National Health Policy Forum)*, *757*, 1–12. | 2000 | editorials, commentaries, and letters | So far, 37 state legislatures - many of them in the last decade - have passed laws authorizing involuntary outpatient commitment (OPC). In OPC, a court can order a patient to receive treatment in a community setting, providing patients with fewer restrictions than would be possible in a psychiatric institution but allowing professionals to make sure the patients comply with treatment regimens. Before these laws were enacted, the principal alternative available to state and local authorities for coercive intervention on behalf of people with severe mental illness was forcibly institutionalizing them when they became dangerous to themselves or others. This was done through a process of "inpatient commitment," typically following the decision of two psychiatrists that the individual constituted a threat to his or her own safety or that of others. Inpatient commitment is still in use in all 50 states. These days, however, psychiatric hospitalization in the United States serves mostly as a locus of short-term care. | 1 |  |
| Database search | Assessing Relationship Quality in Mandated Community Treatment: Blending Care With Control | Skeem, J. L. : L., J. E. :. Polaschek, D. :. Camp, J. (2007). Assessing Relationship Quality in Mandated Community Treatment: Blending Care With Control. Psychol. Assess., 19(4), 397–410. https://doi.org/10.1037/1040-3590.19.4.397 | 2007 | research article | As observed by Howgego et al. (2003), conventional measures of the therapeutic alliance may poorly fit patients who "do not voluntarily seek help and enter a relationship motivated to engage" (p. 180). Such patients may compose a large group. First, a variety of legal tools are now being used to require that patients attend treatment and take psychotropic medication. Formal treatment mandates come in many forms, including involuntary outpatient commitment and special conditions of probation, which are civil and criminal judicial orders (respectively) for a patient to adhere to a community treatment plan. Based on a sample of 1,000 outpatients drawn from public community outpatient settings, Monahan et al. (2005) found that nearly half (44%-66%) had experienced at least one of four types of formal mandates to participate in treatment. | 1 |  |
| Database search | Use of involuntary outpatient commitment in community care of the seriously and persistently mentally ill patient | Smith, C. A. (1995). Use of involuntary outpatient commitment in community care of the seriously and persistently mentally ill patient. *Issues in Mental Health Nursing*, *16*(3), 275–284. | 1995 | research article | Compliance with outpatient treatment programs and prescribed medication schedules is emphasized to prevent exacerbation of the symptoms in the chronically mentally disabled patient. Involuntary outpatient commitment (IOC) statutes are state civil laws that require mental patients to comply with a community-basedprogram to avoid commitment to an institutional setting (Schwartz & Constanzo, 1987). The IOC mechanism compels persons with mental illness to comply with outpatient treatment (McCafferty & Dooley, 1990). Although the IOC approach can force compliance with treatment, the legal justification and effectiveness of IOC have not been clearly determined. | 1 | Schwartz, S., & Constanzo, C. (1987). Compelling treatment in the community:Distorted doctrines and violated values. Loyola of Los Angeles Law Review, 20, 1329-1429. |
| Database search | Client Outreach in Los Angeles County"s Assisted Outpatient Treatment Program: Strategies and Barriers to Engagement | Starks, S. L. : K., E. L. :. Castillo, E. G. :. Meldrum, M. L. :. Bourgois, P. :. Braslow, J. T. (2020). Client Outreach in Los Angeles County’s Assisted Outpatient Treatment Program: Strategies and Barriers to Engagement. Res. Soc. Work Pract. https://doi.org/10.1177/1049731520949918 | 2020 | research article | AOT - also referred to as involuntary outpatient commitment or community treatment orders - seeks to provide outpatient services to treatment-refusing individuals with SMI who have a recent history of arrests or psychiatric hospitalizations and/or are at risk of clinical deterioration, disability, or harm to self or others. Although AOT programs vary, a key feature is a civil court process in which a judge orders the individual to adhere to a treatment plan within the community. Since there are no legal consequences for nonadherence, it is hoped that individuals will respond to the "black robe" effect of being ordered into treatment by a judge (Kisely et al., 2017). When individuals continue to refuse services, AOT staff also may take them to a hospital for evaluation and/or initiate additional efforts to persuade them to accept services (Swartz et al., 2017). | 1 |  |
| Database search | Uses of coercion in addiction treatment: Clinical aspects | Sullivan, M. A. : B., F. :. Boyarsky, B. K. :. Frances, R. J. :. Fromson, J. A. :. Galanter, M. :. Levin, F. R. :. Lewis, C. :. Nace, E. P. :. Suchinsky, R. T. :. Tamerin, J. S. :. Tolliver, B. :. Westermeyer, J. (2008). Uses of coercion in addiction treatment: Clinical aspects. American Journal on Addictions, 17(1), 36–47. https://doi.org/10.1080/10550490701756369 | 2008 | review article | Coerced or involuntary treatment comprises an integral, often positive component of treatment for addictive disorders. | 1 |  |
| Database search | Balancing Medical Ethics to Consider Involuntary Administration of Electroconvulsive Therapy | Surya, S. : B., R. J. :. Shashank, R. B. (2019). Balancing Medical Ethics to Consider Involuntary Administration of Electroconvulsive Therapy. Journal of ECT, 35(3), 150–151. https://doi.org/10.1097/YCT.0000000000000624 | 2019 | review article | Do they reject their prescribed treatment, or do they agree with the generally favorable view of ECT reported by individuals who receive consensual ECT? Is the risk-benefit ratio of involuntary (without individual consent) treatments similar to that of voluntary treatments (with individual consent). In this context, surveys by Takamiya et al5 and Besse et al,6 published in this issue of the journal, make important contribution to the field of ECT | 2 |  |
| Database search | Involuntary out-patient commitment and reduction of violent behaviour in persons with severe mental illness | Swanson, J. W. : S., M. S. :. Borum, R. :. Hiday, V. A. :. Wagner, H. R. :. Burns, B. J. (2000). Involuntary out-patient commitment and reduction of violent behaviour in persons with severe mental illness. British Journal of Psychiatry, 176(APR.), 324–331. https://doi.org/10.1192/bjp.176.4.324 | 2000 | research article | Involuntary out-patient commitment (OPC) is a legal intervention designed to benefit individuals with severe mental illness (SMI) who need ongoing psychiatric care and support to prevent dangerous relapse, but who are reluctant or unable to follow through with community based treatment. Proponents of OPC assert that it may improve treatment adherence and may also act as a lever on a mental health service system: mobilising supportive health services and motivating clinical vigilance. This paper addresses the question of whether OPC can help to prevent violent behaviour in psychiatric populations with a history of `revolving-door' hospital admissions often associated with dangerousness. Findings are presented from the first randomised study of the effectiveness of OPC combined with case management (Swartz et al, 1999). ... Involuntary OPC is a promising legal intervention that may significantly reduce violent behaviour associated with these particular problems by improving compliance with medications that mitigate high-risk psychotic symptoms; by improving access to substance misuse treatment for persons with dual diagnoses; by increasing clinical surveillance; and by augmenting case management intensity, thus leveraging scarce resources in community care systems (Swanson et al 1997b). | 2 | Swartz, M., Swanson, J., Hiday, V. ,Wagner, H., et al (1999) Can involuntary outpatient commitment reduce hospital recidivism? Findings from a randomised trial in severely mentally ill individuals. American Journal of Psychiatry, 156, 1968-1975. |
| Database search | Effects of involuntary outpatient commitment on subjective quality of life in persons with severe mental illness | Swanson, J. W. : S., M. S. :. Elbogen, E. B. :. Wagner, H. R. :. Burns, B. J. (2003). Effects of involuntary outpatient commitment on subjective quality of life in persons with severe mental illness. Behavioral Sciences and the Law, 21(4), 473–491. https://doi.org/10.1002/bsl.548 | 2003 | research article | Legally mandated mental health treatment in the community, also known as involuntary outpatient commitment (OPC), poses a particular challenge with respect to such considerations of benefit to consumers’ quality of life. On its face, OPC seems to preclude patient autonomy in treatment decision-making, insofar as it is, almost inherently, an unwanted intervention from the point of view of individuals subjected to it. At the same time, the main goal of OPC is to enable more consistent adherence to treatment (presumably needed and beneficial) for persons whose disorders by nature tend to impair one’s ability to seek and comply voluntarily with treatment. Paradoxically, then, insofar as individual autonomy in decision-making is a component of a high quality of life, OPC may theoretically exert both negative and positive effects. By ensuring more consistent treatment and services delivery, it is hypothesized OPC may enhance quality of life. However, by infringing on an individual’s liberty—suspending his or her right to refuse treatment in the community—OPC could also detract from quality of life, as perceived by the person under court-ordered treatment (Swartz, Wagner, Swanson, Hiday, & Burns, 2002). | 1 |  |
| Database search | Interpreting the effectiveness of involuntary outpatient commitment: A conceptual model | Swanson, J. W. : S., M. S. :. George, L. K. :. Burns, B. J. :. Hiday, V. A. :. Borum, R. :. Wagner, H. R. (1997). Interpreting the effectiveness of involuntary outpatient commitment: A conceptual model. *Journal of the American Academy of Psychiatry and the Law*, *25*(1), 5–16. | 1997 | research article | The primary independent variable in this model is the court order to comply with OPC, a formal, legally sanctioned use of coercion applied to the behavior of mentally ill individuals. | 1 |  |
| Database search | Gun-Related and Other Violent Crime After Involuntary Commitment and Short-Term Emergency Holds | Swanson, J. W. : T., G. :. Robertson, A. G. :. Swartz, M. S. (2020). Gun-Related and Other Violent Crime After Involuntary Commitment and Short-Term Emergency Holds. The Journal of the American Academy of Psychiatry and the Law, 48(4), 454–467. https://doi.org/10.29158/JAAPL.200082-20 | 2020 | research article | Deinstitutionalization and a tightening of civil commitment statutes across the country brought a steep decline in use of involuntary civil commitment, which had served not only as a legal means to confine persons with incapacitating mental illness who required inpatient-level treatment to mitigate dangerousness, but also as a mechanism for firearm disqualification applied to such individuals. A corresponding increase occurred in short-term involuntary holds for emergency psychiatric evaluation, which usually do not result in extended inpatient treatment or confer loss of gun rights. | 1 |  |
| Database search | The ethical challenges of a randomized controlled trial of involuntary outpatient commitment | Swartz, M. S. : B., B. J. :. George, L. K. :. Swanson, J. :. Hiday, V. A. :. Borum, R. :. Wagner, H. R. (1997). The ethical challenges of a randomized controlled trial of involuntary outpatient commitment. *Journal of Mental Health Administration*, *24*(1), 35–43. | 1997 | review article | Involuntary outpatient commitment (OPC) is a civil justice procedure intended to enhance compliance with community mental health treatment, to improve functioning, and to reduce recurrent dangerousness and hospital recidivism. ... This article focuses on efforts to study the effectiveness of court-ordered community-based mental health treatment, referred to as involuntary outpatient commitment (OPC). | 2 |  |
| Database search | Involuntary Outpatient Commitment and the Elusive Pursuit of Violence Prevention: A View from the United States | Swartz, M. S. : B., S. :. Robertson, A. G. :. Swanson, J. W. (2017). Involuntary Outpatient Commitment and the Elusive Pursuit of Violence Prevention: A View from the United States. Canadian Journal of Psychiatry, 62(2), 102–108. https://doi.org/10.1177/0706743716675857 | 2017 | review article | Involuntary outpatient commitment (OPC) - also referred to as "assisted outpatient treatment" or "community treatment orders" - are civil court orders whereby persons with serious mental illness and repeated hospitalisations are ordered to adhere to community-based treatment. ...  Involuntary outpatient civil commitment (OPC), as implemented in the United States, is a civil court procedure wherein a judge directs a person with severe mental illness to adhere to an outpatient treatment plan designed to prevent relapse and dangerous deterioration.1,4,10 Typically, enforcement of compliance with an OPC order involves law enforcement transporting a noncompliant patient to a treatment facility for clinical reevaluation and an attempt to persuade the patient to comply with the treatment plan. Involuntary administration of medication is usually explicitly prohibited under the authority of OPC and, if indicated, requires separate legal authority and procedures for administration of involuntary medication.10 | 2 | 1. Monahan J, Bonnie RJ, Appelbaum PS, et al. Mandated community treatment: beyond outpatient commitment. Psychiatr Serv. 2001;52(9):1198-1205. 4. Swanson JW, Swartz MS, George LK, et al. Interpreting the effectiveness of involuntary outpatient commitment: a conceptual model. J Am Acad Psychiatry Law. 1997;25(1): 5-16. 10. Gerbasi JB, Bonnie RJ, Binder RL. Resource document on mandatory outpatient treatment. J Am Acad Psychiatry Law. 2000;28(2):127-144. |
| Database search | A randomized controlled trial of outpatient commitment in north carolina | Swartz, M. S. : S., J. W. :. Hiday, V. A. :. Wagner, H. R. :. Burns, B. J. :. Borum, R. (2001). A randomized controlled trial of outpatient commitment in north carolina. Psychiatric Services, 52(3), 325–329. https://doi.org/10.1176/appi.ps.52.3.325 | 2001 | research article | "Involuntary outpatient commitment is a legal intervention intended to benefit severely mentally ill persons who need ongoing psychiatric care to prevent relapse, rehospitalizaton, and dangerous behavior and who have difficulty following through with community-based treatment (1-4). In all but a small minority of states, the criteria for outpatient commitment are identical to those for inpatient commitment, which limits the preventive use of outpatient commitment. However, one relatively recent variant of outpatient commitment implemented in several states allows for a preventive form. Outpatient commitment orders typically require that patients comply with recommended outpatient treatment. Some outpatient commitment statutes stop short of permitting forced medication, as in North Carolina, where this study took place." | 1 | cited the following 4 articles for the definition:   1. McCafferty G, Dooley J: Involuntary outpatient commitment: an update. Mental and Physical Disability Law Reporter 14:277‚Äì287, 1990   2. Hiday VA: Coercion in civil commitment: process, preferences, and outcome. International Journal of Law and Psychiatry 15:359‚Äì377, 1992   3. Swartz MS, Burns BJ, Hiday VA, et al: New directions in research on involuntary outpatient commitment. Psychiatric Services 46:381‚Äì385, 1995   4. Torrey EF, Kaplan RJ: A national survey of the use of outpatient commitment. Psychiatric Services 46:778‚Äì784, 1995 |
| Database search | Effects of involuntary outpatient commitment and depot antipsychotics on treatment adherence in persons with severe mental illness | Swartz, M. S. : S., J. W. :. Wagner, H. R. :. Burns, B. J. :. Hiday, V. A. (2001). Effects of involuntary outpatient commitment and depot antipsychotics on treatment adherence in persons with severe mental illness. Journal of Nervous and Mental Disease, 189(9), 583–592. https://doi.org/10.1097/00005053-200109000-00003 | 2001 | research article | Involuntary outpatient commitment (OPC), wherein a judge orders a patient to adhere to treatment, is designed to benefit individuals with SMI who are reluctant or unable to follow through with community-based treatment. | 1 |  |
| Database search | Assessment of four stakeholder groups' preferences concerning outpatient commitment for persons with schizophrenia | Swartz, M. S. : S., J. W. :. Wagner, H. R. :. Hannen, M. J. :. Burns, B. J. :. Shumway, M. (2003). Assessment of four stakeholder groups’ preferences concerning outpatient commitment for persons with schizophrenia. American Journal of Psychiatry, 160(6), 1139–1146. https://doi.org/10.1176/appi.ajp.160.6.1139 | 2003 | research article | Involuntary outpatient commitment is a civil procedure whereby a judge orders a person with mental illness to comply with outpatient treatment or risk sanctions such as being forcibly brought to treatment by law enforcement officers. | 1 |  |
| Database search | Economic grand rounds: Can states implement involuntary outpatient commitment within existing state budgets? | Swartz, M. S. : S., Jeffrey W. (2013). Economic grand rounds: Can states implement involuntary outpatient commitment within existing state budgets? Psychiatric Services (Washington, D.C.), 64(1), 7–9. https://doi.org/10.1176/appi.ps.201200467 | 2013 | research article | Involuntary outpatient commitment is a controversial policy that involves providing court-ordered community services to adults with severe mental illness who are nonadherent to treatment. Research has shown, with some exceptions, that sustained court ordered outpatient treatment can improve a range of consumer outcomes (1,2). | 1 |  |
| Database search | The perceived coerciveness of involuntary outpatient commitment: Findings from an experimental study | Swartz, M. S. : W., H. R. :. Swanson, J. W. :. Hiday, V. A. :. Burns, B. J. (2002). The perceived coerciveness of involuntary outpatient commitment: Findings from an experimental study. *Journal of the American Academy of Psychiatry and the Law*, *30*(2), 207–217. | 2002 | research article | One form of court-ordered treatment, involuntary outpatient commitment (OPC), is a civil procedure intended to improve adherence to treatment by ordering a patient to comply with the outpatient regimen. OPC is permitted in some form in virtually all states in the U.S. | 1 |  |
| Hand search organization/agency/association website | RESOURCE DOCUMENT ON INVOLUNTARY OUTPATIENT COMMITMENT AND RELATED PROGRAMS OF ASSISTED OUTPATIENT TREATMENT | Swartz, M. S., Hoge, S. K., Pinals, D. A., Lee, E., Lee, L.-W., Sidor, M., Bell, T., Ford, E., & Scott Johnson, R. (2015). RESOURCE DOCUMENT ON INVOLUNTARY OUTPATIENT COMMITMENT AND RELATED PROGRAMS OF ASSISTED OUTPATIENT TREATMENT (APA Resource Document). American Psychiatric Association. | 2015 | Educational materials | Involuntary outpatient commitment is a form of court-ordered outpatient treatment for patients who suffer from severe mental illness and who are unlikely to adhere to treatment without such a program. It can be used as a transition from involuntary hospitalization, an alternative to involuntary hospitalization or as a preventive treatment for those who do not currently meet criteria for involuntary hospitalization. It should be used in each of these instances for patients who need treatment to prevent relapse or behaviors that are dangerous to self or others.” o 1 Outpatient court-ordered treatment may be referred to as ‘assisted outpatient treatment’, ‘involuntary outpatient commitment’, ‘mandated community treatment’, or ‘community treatment orders’. Some regard the term ‘assisted outpatient treatment’ as a euphemistic term for treatment under coercion. In this document the term ‘involuntary outpatient commitment’ is used to refer to these programs. The current document is adapted from: Gerbasi JB, Bonnie RJ, Binder RL: Resource document on mandatory outpatient treatment. Journal of the American Academy of Psychiatry and the Law 2000; Vol 28(2): 127-144 o Involuntary outpatient commitment is a civil court procedure wherein a judge orders a person with severe mental illness to adhere to an outpatient treatment plan designed to prevent relapse and dangerous deterioration (2-4). Persons appropriate for this intervention are those who need ongoing psychiatric care owing to severe mental illness but who are unable or unwilling to engage in ongoing, voluntary, outpatient care. It should be distinguished from ‘conditional release,’ a form of treatment wherein a patient committed to an inpatient hospital is released to the community but remains under the ongoing supervision of the hospital -- if the patient’s condition deteriorates he or she can be returned to the hospital (see Figure 1.). Additionally, there are three types of involuntary outpatient commitment: 1) the most common type is outpatient commitment as part of a discharge plan from an involuntary hospitalization; 2) an alternative to hospitalization for patients who otherwise meet the criteria for involuntary hospitalization; and 3) a ‘preventive’ treatment for those patients who do not presently meet criteria for inpatient hospitalization, but who are in need of treatment to prevent such decompensation. Orders initiated as a ‘stepdown’ from involuntary inpatient commitment (Type 1) are often later renewed as a method to prevent relapse (Type 3). Figure 1. General types of involuntary outpatient commitment Type 1 Post-discharge involuntary outpatient commitment plan unattached to hospital supervision Type 2 Alternative to hospitalization for those meeting civil commitment criteria but for whom outpatient commitment is sufficient Type 3 Preventive treatment for individuals who do not meet criteria for inpatient hospitalization but are in need of treatment to prevent decompensation Although recently enacted statutes use the term ‘assisted outpatient treatment’, other phrases, such as ‘mandatory outpatient treatment’, ‘community treatment orders’ or ‘involuntary outpatient commitment,’ are also in use. The phrase “involuntary outpatient commitment” implies a more coercive approach than is envisioned by proponents of judicial treatment orders, however the term ‘assisted outpatient treatment’ is sometimes criticized as euphemistic. In practice, these legal devices are intended to reinforce the patient’s own resolve to adhere to a treatment plan while marshalling the resources of local mental health authorities to more effectively serve the patient. In this Resource Document, the phrase ‘involuntary outpatient commitment’ will be used. In addition with a few exceptions the Document will focus on U.S. experience with outpatient commitment. | 3 | 2. Gerbasi JB, Bonnie RJ, Binder RL: Resource document on mandatory outpatient treatment. Journal of the American Academy of Psychiatry and the Law 2000; Vol 28(2): 127-144 3. Monahan J, Swartz M, Bonnie R: Mandated treatment in the community for people with mental disorders. Health Affairs 2003; 22(5):28-38 4. Swartz MS, Swanson, JW: Involuntary outpatient commitment in the United States: Practice and Controversy, in A. Buchanan (ed), Care of the Mentally Ill Disordered Offender in the Community. Oxford University Press 1999a |
| Database search | Coercion as cure: A critical history of psychiatry | Szasz, T. (2017). Coercion as cure: A critical history of psychiatry. In Coercion as Cure: A Critical History of Psychiatry (p. 278). Taylor and Francis. https://www.scopus.com/inward/record.uri?eid=2-s2.0-85065331660&doi=10.4324%2f9781315080994&partnerID=40&md5=bb78cf88f20f0328c37646232cd37ee9 | 2017 | Book excerpt | The typical relationship between doctor and patient rests, and has always rested, on consent. In contrast, the typical relationship between mad-doctor and madman rests, and has always rested, on coercion. The paradigmatic psychiatric act is the use of force, which takes two forms. One is involuntary mental hospitalization, called "civil commitment," the alleged justification for this deprivation of liberty being the need to protect the patient from himself (his illness), and the public from the patient (his dangerousness). The other intervention is the excusing of the person guilty of a crime by attributing his unlawful behavior to mental illness, a practice called the "insanity defense." The alleged justification for this deprivation of liberty is that it "humanizes" the criminal law by sentencing the mentally ill offender to treatment instead of punishment. In both cases, the result is the subject"s incarceration, called "hospitalization." Once we recognize these facts, and the self-evidently fictitious nature of mental illnesses, the barriers identified by Hunter and Macalpine against writing a definitive history of psychiatry disappear. Perforce, such a history must differ radically from conventional hagiographies of mad-doctoring modeled on histories of medicine. | 1 |  |
| Database search | Voluntary Mental Hospitalization: An Unacknowledged Practice of Medical Fraud | Szasz, T. S. (1972). Voluntary Mental Hospitalization: An Unacknowledged Practice of Medical Fraud. New Engl. J. Med., 287(6), 277–278. https://doi.org/10.1056/NEJM197208102870604 | 1972 | editorials, commentaries, and letters | THERE are, so our language and laws tell us, two types of mental hospitalization: voluntary and involuntary.* This terminology would lead us to believe that voluntary mental hospitalization occurs when a person defines himself as a mental patient and seeks admission to a mental hospital, which he is free to leave when he wishes, and that involuntary mental hospitalization occurs when someone is defined as a mental patient by others and is confined in a mental hospital against his will until such time as those in charge of him release him. Actually, only half of the foregoing statement is correct: involuntary mental hospitalization is just that - hospitalization in opposition to the will of the so-called patient. The other half is incorrect: voluntary mental hospitalization is often actually a type of involuntary psychiatric confinement. ...  In their comprehensive review of "The Rights of the Mentally IlI," Lindman and McIntyre devote a chapter to "Voluntary Admission."' The semantics is, again, important: Chapter 2 of their book is enti-ted "Involuntary Hospitalization," and Chapter 3 "Voluntary Admission." There is no chapter entitled "Voluntary Hospitalization." Perhaps this is because there is no such thing. Moreover, not even the "voluntary admissions" considered by these authors are truly voluntary. "As used in this Report," they write, "the term 'voluntary admission' refers to procedures for admission to a mental hospital which are commenced originally by the affirmative action of the patient himself or of someone empowered by law to act in the patient's behalf." | 2 | Webster's dictionary |
| Database search | Medicine and madness | Szasz, T. S. (1975). Medicine and madness. *Journal of Psychiatry & Law*, *3*(2), 215–222. | 1975 | Book excerpt | The most important deprivation of human and constitutional rights inflicted on persons said to be mentally ill is involuntary mental hospitalization; that is, the coerced confinement of the person in an institution called a mental hospital. Many thousands of persons are now so confined in the United States, and countless more in other countries. While the precise legal requirements for commitment differ from state to state and from nation to nation, the procedure, in effect, is based on and justified by the closely connected ideas of mental illness and dangerousness. This is exemplified by the traditional American legal formula for commitment, which speaks of the alleged patient as suffering from a "mental disease or disorder" and of being "dangerous to himself or others." Regardless of the legal phraseology in which commitment laws are enshrouded, their implementation depends almost entirely on the ideology that animates the psychiatrists and judges who practice this sort of "medicine." Their ideology has been, quite simply, paternalistic. ... Especially in contemporary free societies, there is, for all practical purposes, no such thing as the involuntary medical treatment of adults. The social act of medical treatment comes into being not so much because the patient is sick as because he wants treatment and is willing to submit to it. In short, what ultimately justifies medical treatment is not disease but consent. Conversely, what characterizes involuntary psychiatric diagnosis, hospitalization, and treatment is that each comes into being not because the alleged patient wants or is willing to submit to it, but because someone other than the "patient" claims that the "patient" is "mentally ill." | 2 |  |
| Database search | The myth of mental illness (1960) | Szasz, T. S. (2009). The myth of mental illness (1960). In Foundations of psychological thought: A history of psychology. (2008-13609-035; pp. 577–588). Sage Publications, Inc. https://search.ebscohost.com/login.aspx?direct=true&db=psyh&AN=2008-13609-035&site=ehost-live | 1960 | Book excerpt | Such considerations lead to two diametrically opposed points of view about mental illness and psychiatry. According to the traditional and at present generally accepted view, mental illness is like any other illness; psychiatric treatment is like any other treatment; and psychiatry is like any other medical specialty. According to the view I have endeavored to develop and clarify, however, there is, and can be, no such thing as mental illness or psychiatric treatment; the interventions now designated as "psychiatric treatment" must be clearly identified as voluntary or involuntary: voluntary interventions are things a person does for himself in an effort to change, whereas involuntary interventions are things done to him in an effort to change him against his will; and psychiatry is not a medical, but a moral and political, enterprise. This book is an attempt to demonstrate the fallacy of the former view and the validity of the latter. ... Another way of distinguishing among the various psychiatric interventions is by dividing them into two classes - voluntary and involuntary. The typical voluntary psychiatric interventions are psychoanalysis, the various types of individual and group psychotherapy, and a great variety of both office and hospital psychiatry employing psychological or physical methods of treatment with the informed consent of the patient. Typical involuntary psychiatric interventions are commitment or measures carried out under the threat of commitment, and psychiatric "diagnoses" and "treatments" imposed on persons by parents, schools, courts, military authorities, and other social or governmental agencies. | 2 |  |
| Database search | Putting values into practice: Involuntary treatment interventions in mental health | Taylor, M. F. (2006). Putting values into practice: Involuntary treatment interventions in mental health. In Community mental health: Challenges for the 21st century. (2006-05879-007; pp. 73–81). Routledge. https://search.ebscohost.com/login.aspx?direct=true&db=psyh&AN=2006-05879-007&site=ehost-live | 2006 | Book excerpt | Involuntary treatment can be defined as "mandated services, both inpatient and outpatient, that are provided to consumers, often despite their wishes to the contrary" (Taylor, 2002, p. 56). | 1 | (Taylor, 2002, p. 56). Tavlor, M.F. (2002). Professional dissonance among social workers: the collision between values and job tasks in mental health practice (doctoral dissertation, Virginia Commonwealth University) Dissertation Abstracts International, 63, 2000. |
| Database search | "Assisted outpatient treatment": an example of newspeak? | Torrey, E. F. : S., Jonathan. (2013). “Assisted outpatient treatment”: An example of newspeak?: In reply. Psychiatric Services (Washington, D.C.), 64(11), 1179–1180. https://doi.org/10.1176/appi.ps.641109 | 2013 | editorials, commentaries, and letters | In the first case, we assume that the man has a normally functioning brain and can make informed choices. Forcing him to take medication is truly involuntary treatment. In the second, we know that the man does not have a normally functioning brain and suspect he cannot make informed choices. Forcing him to take medication is assisting him to make the choice we think he would make if he had a normally functioning brain. | 1 |  |
| Database search | Voluntariness to consent to research in a voluntarily and involuntarily hospitalized psychiatric population | Van Der Veer, N. L. : D., D. :. Ahad, S. :. Silvers, G. :. Ramos, G. (2011). Voluntariness to consent to research in a voluntarily and involuntarily hospitalized psychiatric population. Journal of Empirical Research on Human Research Ethics, 6(1), 55–61. https://doi.org/10.1525/jer.2011.6.1.55 | 2011 | research article | Involuntary and voluntary statuses are legal terms in the United States that refer to a person"s willingness to be hospitalized and obtain psychiatric treatment when they are considered dangerous to themselves or others. | 1 |  |
| Hand search organization/agency/association website | INPATIENT MENTAL HEALTH SERVICES - VHA HANDBOOK | VHA. (2013). INPATIENT MENTAL HEALTH SERVICES - VHA HANDBOOK 1160.06. Department of Veterans Affairs, Veterans Health Administration. | 2013 | educational materials | (1) page 3/5 under the definition section “. Commitment and Involuntary Mental Health Treatment: Commitment and involuntary mental health treatment refer to the totality of applicable state laws governing involuntary mental health evaluation and treatment, including time limited holds for evaluation, involuntary outpatient treatment, and forced administration of psychotropic medication.” | 1 |  |
| Database search | Does involuntary outpatient commitment lead to more intensive treatment? | Wagner, H. R. : S., M. S. :. Swanson, J. W. :. Burns, B. J. (2003). Does involuntary outpatient commitment lead to more intensive treatment? Psychology, Public Policy, and Law, 9(1–2), 145–158. https://doi.org/10.1037/1076-8971.9.1-2.145 | 2003 | research article | Many states have implemented the use of involuntary outpatient commitment (OPC) with the goal of reducing high rates of relapse and other negative outcomes among persons with severe mental illness (SMI; Swartz & Monahan, 2001). OPC is a civil court procedure in which a judge orders a patient to comply with recommended treatment or risk sanctions such as being forcibly transported to treatment by law enforcement officers. | 1 |  |
| Database search | Involuntary Civil Commitment for Substance Use Disorder: Legal Precedents and Ethical Considerations for Social Workers | Walton, M. T. : H., M. T. (2017). Involuntary Civil Commitment for Substance Use Disorder: Legal Precedents and Ethical Considerations for Social Workers. Social Work in Public Health, 32(6), 382–393. https://doi.org/10.1080/19371918.2017.1327388 | 2017 | research article | In an effort to prevent negative health outcomes and deaths resulting from SUD, several U. S. states have passed involuntary civil commitment (ICC) laws that allow concerned persons to petition local courts to forcibly remand individuals with SUDs to treatment (Christopher, Pinals, Stayton, Sanders, & Blumberg, 2015). These laws uphold that a severe SUD that endangers self and/or others satisfies necessary criteria to legally override an individual"s right to refuse treatment.  AND: In ICC cases, concerned persons (e.g., family members, physicians, police officers) petition the court to enforce a mandate that an individual enter substance use treatment without needing to rely on the criminal court system. In these hearings, the petition activates the court"s power to issue a ruling rather than a criminal charge, and if the judge grants the petition, the ICC process proceeds and the individual is mandated to drug addiction treatment. This route offers benefits not present in criminal hearings. Foremost, it allows for intervention before circumstances have become so severe that criminal charges have been filed. This may allow individuals to avoid the challenges associated with carrying a criminal record, which include limited employment opportunities (Saxonhouse, 2004), access to credit (Henderson, 2005), and property rental (Clark, 2007). In sum, earlier intervention, facilitated by ICC laws, is intended to reduce harms and respond more quickly to improve future well-being for individuals with SUDs. | 2 | Christopher, P. P., Pinals, D. A., Stayton, T., Sanders, K., & Blumberg, L. (2015). Nature and utilization of civil commitment for substance abuse in the United States. Journal of the American Academy of Psychiatry and the Law Online, 43(3), 313–320. |
| Database search | A brief review of the ethical and legal concerns of treating voluntary psychiatric patients on a locked unit | Warner, M. D. : E., M. L. :. Dorn, M. R. :. Peabody, C. A. (2000). A brief review of the ethical and legal concerns of treating voluntary psychiatric patients on a locked unit. *American Journal of Forensic Psychiatry*, *21*(1), 5–18. | 2000 | review article | Historically, mental institutions were asylums for confinement and virtually all patients were confined involuntarily (i.e., hospitalized in locked facilities without their consent). Civil commitment was based on a parens patriae concept of acting in place of the parent and protecting those individuals who could not care for themselves. | 1 |  |
| Database search | Conclusion: How to learn more about forensic psychiatry | Wasser, T. (2017). Conclusion: How to learn more about forensic psychiatry. In Psychiatry and the Law: Basic Principles (pp. 183–188). Springer International Publishing. https://www.scopus.com/inward/record.uri?eid=2-s2.0-85043334567&doi=10.1007%2f978-3-319-63148-6_16&partnerID=40&md5=acef3bb30638681e701e453193e83bba | 2017 | Book excerpt | Involuntary or civil commitment, a legal process through which an individual with symptoms of severe mental illness is court-ordered into psychiatric treatment. (p 65)  Involuntary admission is the process by which a person can be admitted to a psychiatric hospital or psychiatric unit within a general hospital against his or her will (1). (p 55) | 2 | 1. Hashmi A, Shad M, Rhoades HM, Parsaik AK. Involuntary detention: do psychiatrists clinically justify continuing involuntary hospitalization? Psychiatry Q. 2014;85:285–93. |
| Database search | Mental Institutions: Legal Issues and Commitments | Weinberger, L. E. : M., E. (2016). Mental Institutions: Legal Issues and Commitments. In *Encycl. Of Ment. Health: Second Ed.* (pp. 123–132). Elsevier Inc. | 2016 | Book excerpt | Treatment in a psychiatric hospital may be categorized as 'voluntary or 'involuntary.' Voluntary patients are those who seek psychiatric treatment and consent to obtaining it in mental institutions. They retain the right to accept or reject treatment as well as to leave the hospital without legal con- straints. Although the patient has these rights, many psychiatric hospitals do not admit voluntary patients unless they are willing to accept the treatment provided and not request discharge before a specified period of time deemed necessary for adequate treatment. Although voluntary batients outnumber involuntary patients today, this is a relatively recent occurrence. Civil commitment historically and legally involved involuntary psychiatric comitment (Rosner, 2003). | 1 |  |
| Database search | When Does the "Duty to Protect" Apply with a Client Who has Anorexia Nervosa? | Werth, J. L., Jr. :. Wright, K. S. :. Archambault, R. J. :. Bardash, R. J. (2003). When Does the “Duty to Protect” Apply with a Client Who has Anorexia Nervosa? Couns. Psychol., 31(4), 427–450. https://doi.org/10.1177/0011000003031004006 | 2003 | review article | Distinguishing between involuntary hospitalization and compulsory treatment is important. In the treatment of eating disorders, involuntary hospitalization refers to the client"s being placed into a restrictive environment where she is able to partake of the same treatment program as voluntary clients. Compulsory treatment is more specific and may include forced or nasogastric feeding. This distinction is not always made clear in the literature, but it is significant in the United States because of the assumption that a person is competent, and therefore able to refuse treatment, unless proven otherwise in court. | 1 |  |
| Database search | The right to refuse psychiatric treatment | Wettstein, R. M. (1999). The right to refuse psychiatric treatment. Psychiatric Clinics of North America, 22(1), 173–182. https://doi.org/10.1016/S0193-953X(05)70067-8 | 1999 | review article | When clinical or family intervention fails to prompt hospitalized patients to accept medication that is critical to the overall treatment plan, the staff needs to consider, and discuss with patients, the possibility of discharge to the community or a partial hospital program. Patients may protest this, and involuntary discharge (i.e., over the patient's objection) can be implemented when safe to do so. Of course, patients may continue to refuse the necessary medication during their outpatient care. Clinic staffs then have the responsibility to address patients' reasons for refusal. Repeat hospitalizations may be needed before patients come to accept the need for treatment. | 1 |  |
| Database search | Implications of involuntary outpatient commitment for community mental health agencies | Wilk, R. J. (1988a). Implications of involuntary outpatient commitment for community mental health agencies. American Journal of Orthopsychiatry, 58(4), 580–591. https://doi.org/10.1111/j.1939-0025.1988.tb01622.x | 1988 | review article | According to Keilitz and Hall (1985), " . . . involuntary outpatient commitment is the legal and psychosocial process whereby an allegedly mentally disordered and dangerous person is forced to undergo mental health treatment or care in an outpatient setting (p. 378)." The idea itself is not new; it was discussed in the literature as early as 1967 (Bleicher, 1967). | 1 | Keilitz, I., & Hall, T. (1985). State statutes governing involuntary outpatient civil commitment. Mental & Physical Disability Law Reporter, 9(5), 378-397. |
| Database search | Involuntary outpatient commitment of the mentally III | Wilk, R. J. (1988b). Involuntary outpatient commitment of the mentally III. Soc. Work, 33(2), 133–137. https://doi.org/10.1093/sw/33.2.133 | 1988 | review article | Simply put, involuntary outpatient commitment is "the legal and psychosocial process whereby an allegedly mentally disordered and dangerous person is forced to undergo mental health treatment or care in an outpatient setting."4 It is usually, though not necessarily, under the supervision of a community mental health center. Treatment most commonly consists of mandatory medication and little else; however, this varies by state. For example, in Arizona, the statute prohibits a patient from going to certain places, or associating with certain people. | 1 | I. Keilitz and T. Hall, "State Statutes Governing Involuntary Outpatient Civil Commitment," Mental & Physical Disability Law Reporter, 9 (September-October 1985), p. 378. |
| Database search | Resource Document for Electroconvulsive Therapy in Adult Correctional Settings | Williams, J. B. : A., M. M. (2021). Resource Document for Electroconvulsive Therapy in Adult Correctional Settings. The Journal of ECT, 37(1), 18–23. https://doi.org/10.1097/YCT.0000000000000694 | 2021 | Educational materials | Two additional topics deserving of attention are involuntary ECT (by which we mean ECT over objection) for inmates and ECT for competence restoration within correctional settings. Because there is overlap between these 2 topics, we address them together. | 1 |  |
| Database search | Inpatient treatment for African American women | Williams, S. N. : J., Melissa G. (2022). Inpatient treatment for African American women. In A handbook on counseling African American women: Psychological symptoms, treatments, and case studies. (2022-31334-011; pp. 209–226). Praeger/ABC-CLIO. https://search.ebscohost.com/login.aspx?direct=true&db=psyh&AN=2022-31334-011&site=ehost-live | 2022 | Book excerpt | Psychiatric patients enter into inpatient facilities on a voluntary or involuntary basis. Involuntary patients are placed in hospitals under their state's civil or forensic commitment statutes (Lutterman et al., 2017). | 1 | Lutterman, T., Shaw, R., Fisher, W. H., & Manderscheid, R. (2017). Trend in psychiatric inpatient capacity, United States and each state, 1970 to 2014. Alexandria, VA: National Association of State Mental Health Program Directors. https:// www.nasmhpd.org/sites/default/files/TACPaper.2.Psychiatric-Inpatient -Capacity_508C.pdf |
| Database search | Lengths of stay for involuntarily held psychiatric patients in the ED are affected by both patient characteristics and medication use | Wilson, M. P. : B., J. J. :. Modesti, L. :. Deen, J. :. Anderson, L. :. Vilke, G. M. :. Castillo, E. M. (2015). Lengths of stay for involuntarily held psychiatric patients in the ED are affected by both patient characteristics and medication use. American Journal of Emergency Medicine, 33(4), 527–530. https://doi.org/10.1016/j.ajem.2015.01.017 | 2015 | research article | One population that is relatively understudied with respect to longer hold times in the ED is patients who have been placed on involuntary mental health holds. Involuntary mental health holds are a legal type of restraint in which patients are not allowed to leave the ED [15-17]. | 1 | [15] Vilke GM, Wilson MP. Agitation: what every emergency physician should know. Emerg Med Rep 2009;30(19):233‚Äì44.  [16] Wilson MP, Sloane C. Chemical restraints, physical restraints, and other demonstrations of force. In: Jesus J, Rosen P, Adams J, Derse A, Wolfe R, Grossman S, editors. Ethical problems in emergency medicine: a discussion-based review. Oxford: Wiley-Blackwell; 2012. p. 139‚Äì48.  [17] Wilson MP, Nordstrom K, Zeller SL. Practical management of the suicidal patient in the emergency department. Emerg Med Rep 2014;35(1):1‚Äì12 |
| Database search | Voluntary and involuntary hospitalization | Yarnell, S. : K., R. (2017). Voluntary and involuntary hospitalization. In Psychiatry and the Law: Basic Principles (pp. 53–61). Springer International Publishing. https://www.scopus.com/inward/record.uri?eid=2-s2.0-85043340311&doi=10.1007%2f978-3-319-63148-6_5&partnerID=40&md5=540d7db844fbb4024f80c6015da567c2 | 2017 | Educational materials | Treatment of acute psychiatric illness frequently requires hospitalization. In some instances, patients recognize the need for hospitalization and agree to be admitted for their own safety. Other times, patients refuse or are unable to understand the need for hospital admission. In these situations, psychiatrists must pursue an emergency involuntary admission (henceforth referred to as involuntary admission). Involuntary admission is the process by which a person can be admitted to a psychiatric hospital or psychiatric unit within a general hospital against his or her will [1]. In the United States, criteria for involuntary psychiatric hospitalization vary from state to state but customarily require a diagnosis of mental illness and either a risk of harm to self or others or evidence of deterioration in the person's mental condition to the extent that the person cannot care for himself or herself. | 1 | Hashmi A, Shad M, Rhoades HM, Parsaik AK.¬†Involuntary detention: do psychiatrists clinically justify continuing involuntary hospitalization? Psychiatry Q. 2014;85:285‚Äì93. |
| Hand search organization/agency/association website | Ethics Considerations of Involuntary Outpatient Treatment | Zilber, C. (2016, November 29). Ethics Considerations of Involuntary Outpatient Treatment. APA Publishing -  Psychiatric News. https://doi.org/10.1176/appi.pn.2016.12a16 | 2016 | magazine style article | On December 2015, the APA Board of Trustees adopted a position statement on involuntary outpatient commitment, defined as “a civil court procedure wherein a judge orders a person with severe mental illness to adhere to an outpatient treatment plan designed to prevent relapse and dangerous deterioration. Persons appropriate for this intervention are those who need ongoing psychiatric care owing to severe illness but who are unable or unwilling to engage in ongoing, voluntary, outpatient care.” Involuntary outpatient treatment pits two important ethical principles against each other, creating a tension between beneficence and autonomy. Beneficence, an ethical principle that dates from the Hippocratic Oath, holds that a physician’s efforts shall be focused on providing treatment that helps a patient. In involuntary outpatient treatment, beneficence derives from the belief that treatment will mitigate symptoms of illness that are so severe that they place the patient or others in danger. Patients who are court-ordered to involuntary outpatient treatment have demonstrated past failures to participate in outpatient treatment, subsequent clinical deterioration, and a return to involuntary inpatient treatment once the patient’s symptoms create an imminent risk of harm once again. | 1 | 2015 APA Board of Trustees position statement on involuntary outpatient commitment |

# 5. Illustrative definitions for (in)voluntary alone and five most common (in)voluntary terms identified in scoping review search

| (In)voluntary term | Illustrative Definitions |
| --- | --- |
| *(In)voluntary* | **Involuntary – decision to commit:** "Being involuntary, the decision to commit a person to a psychiatric hospital is made by persons other than the proposed patient.” (Albers et al., 1976)  **Voluntary – treatment decision:** “Legally, a treatment decision, which is a voluntary, knowledgeable, and competent is informed and thus binding. ‘Voluntary’ means the individual is not coerced into making the decision.” (J. Parry & Beck, 1990)  **Involuntary – state hospital beds:** “Most beds in state hospitals currently are "involuntary" meaning that patients are committed against their will because they are a danger to themselves or others despite the efforts of primary and secondary facilities in the community.” (Davis et al., 1998)  **Involuntary**: “The phrase "outpatient commitment" implies a much more coercive approach than is envisioned by proponents of judicial treatment orders or directives. In practice, these devices are used primarily to reinforce the patient's own resolve and are not imposing treatment against the patient's will (the idea ordinarily conveyed by the term "involuntary").” (Gerbasi et al., 2000)  **Involuntary and voluntary** **– arrived at the hospital**: “Sixty-nine participants arrived at the hospital on their own accord (voluntarily), whereas 48 were brought in involuntary (against the patient’s will).” (Serper et al., 2005)  **Involuntary and voluntary - treatments (ECT):** “Is the risk-benefit ratio of involuntary (without individual consent) treatments similar to that of voluntary treatments (with individual consent)?” (Surya et al., 2019) |
| *Involuntary outpatient commitment* | "Involuntary outpatient commitment has various meanings, and we would like to define our terms before proceeding. For our purposes, involuntary outpatient commitment is the legal commitment of a mentally disordered person to outpatient psychiatric treatment.” (Meloy, 1990)  “Involuntary outpatient commitment is a civil procedure whereby a judge orders a person with mental illness to comply with outpatient treatment or risk sanctions such as being forcibly brought to treatment by law enforcement officers.” (Swartz, 2003)  “Involuntary outpatient commitment is a form of court-ordered outpatient treatment for patients who suffer from severe mental illness and who are unlikely to adhere to treatment without such a program. It can be used as a transition from involuntary hospitalization, an alternative to involuntary hospitalization or as a preventive treatment for those who do not currently meet criteria for involuntary hospitalization. It should be used in each of these instances for patients who need treatment to prevent relapse or behaviors that are dangerous to self or others. … Outpatient court-ordered treatment may be referred to as ‘assisted outpatient treatment’, ‘involuntary outpatient commitment’, ‘mandated community treatment’, or ‘community treatment orders’. Some regard the term ‘assisted outpatient treatment’ as a euphemistic term for treatment under coercion. In this document the term ‘involuntary outpatient commitment’ is used to refer to these programs. …Involuntary outpatient commitment is a civil court procedure wherein a judge orders a person with severe mental illness to adhere to an outpatient treatment plan designed to prevent relapse and dangerous deterioration (2-4). Persons appropriate for this intervention are those who need ongoing psychiatric care owing to severe mental illness but who are unable or unwilling to engage in ongoing, voluntary, outpatient care.”(M. S. Swartz et al., 2015)  “In this document, the term ‘involuntary outpatient commitment’ is used to refer to outpatient treatment mandated under state involuntary commitment statutes. Involuntary outpatient commitment is a civil court procedure wherein a judge orders a person with severe mental illness to adhere to an outpatient treatment plan designed to prevent relapse and dangerous deterioration.” (APA, 2020) |
| *Involuntary hospitalization* | “Involuntary hospitalization, or commitment, may be defined essentially as the legal procedure which confines the mentally ill to an appropriately designated hospital.” (Cranton, 1968)  “C.C. reminds psychiatrists that society views involuntary hospitalization through a special lens: it is not merely an episode of medical treatment, but state-instituted confinement that ‚’can have calamitous effects on an individual... includ[ing] loss of liberty and potential damage to a person's reputation. (C.C., p 108).’” (Abukamil, 2017)  “Involuntary hospitalization: the confinement of a person with a serious mental illness to a mental hospital by medical authorization and legal direction (as in involuntary civil commitment). Individuals so hospitalized may be considered dangerous to themselves or others, may fail to recognize the severity of their illness and the need for treatment, or may be unable to have their daily living and treatment needs otherwise met in the community or to survive without medical attention. Compare voluntary admission.” (APA, 2018)  "Varying by title and subtle nuances, civil commitment is ubiquitous throughout the practice of psychiatry. Defined by the United States Health and Human Services, civil commitment - involuntary hospitalization of a patient, is the legal process by which a person is confined in a psychiatric hospital because of a treatable mental disorder, against his or her wishes” (Fariba, 2021) |
| *Involuntary treatment* | Involuntary treatment is a familiar concept to psychiatrists. In clinical practice, this usually involves the hospitalization and pharmacological management of patients with severe mental disorders.” (Irvin, 2003)  Involuntary treatment (using medications against the person"s will) would proceed if the person were determined dangerous or otherwise incompetent to make decisions concerning his or her own behalf.” (Hunter et al., 2005, p. 200)  “Is the risk-benefit ratio of involuntary (without individual consent) treatments similar to that of voluntary treatments (with individual consent)?” (Surya et al., 2019)  “…the policy makes no provisions for treatment without consent (involuntary treatment), as these are separate legal and therapeutic issues for which relevant statutes and hospital policies already exist.” (Cheung et al., 2018) |
| *Involuntary commitment* | “Involuntary commitment, often referred to as civil commitment, represents the legal process of hospitalizing a person against his or her stated wishes.” (Johnson & Stern, 2014)  “Involuntary commitment, also known as civil commitment, is the legal process of hospitalizing a person against his or her stated wishes.” (Richmond, 2021)  “In order to ensure safety and administer necessary treatment,psychiatrists must resort to involuntary or civil commitment, a legal process through which an individual with symptoms of severe mental illness is court-ordered into psychiatric treatment.” (Nakic, 2017)  “Involuntary or civil commitment, a legal process through which an individual with symptoms of severe mental illness is court-ordered into psychiatric treatment.” (Wasser, 2017) |
| *Involuntary Civil Commitment* | "Involuntary civil commitment is the legal, medical, and psychosocial process whereby a person deemed to be mentally ill and dangerous to self or others and in need of treatment is forced into involuntary mental health care.” (“Guidelines for Involuntary Civil Commitment,” 1986)  "Civil commitment is commonly known as involuntary civil commitment… Civil commitment refers to the process and procedure by which the state mandates hospitalization for persons with mental illness who require incapacitation, treatment, or care due to concerns of self-harm, or harm to others.” (Lareau, 2013)  “Involuntary civil commitment represents an order by a court to a form of care, usually but not always in an inpatient setting, as the vehicle to mandate into treatment specific individuals with mental illness.” (Pinals, 2016) |

# 6. (In)voluntary parts of speech and specific concepts

There were a range of terms where (in)voluntary was a part of speech other than adjective, namely in adverb form (e.g., “involuntarily committed,” “arrived at the hospital involuntarily,” and “hospitalize involuntarily,”) and noun form (e.g., “voluntaries” and “involuntaries,” (S. K. Hoge et al., 1997)). Less commonly, there were also terms that subdivided (in)voluntary concepts, such as “pure voluntary admission” versus “conditional voluntary admission,” “true involuntary patient” (Fisher et al., 2001), “‘formal’ voluntary admissions” versus “’informal’ voluntary admission” (Garakani et al., 2014; E. E. H. Griffith & Etkin, 1981) and “‘involuntary voluntary’ patient” (Leifer, 2018). The term “nonvoluntary” was only used once (Leukefeld & Tims, 1990).

# 7. Repeated definitions

Repeated definitions occurred due to sources in the final inclusion list citing each other. There are six identical or very similar definitions which are included twice and one definition that is included three times. “Involuntary outpatient commitment” had this occur most frequently: Geller (Geller, 1986, p. 198) cited Miller 1984 (Miller, 1994); Johnson (Johnson & Stern, 2014) cited Schouten (Stern et al., 2004); Drogin (Drogin & Spaderna, 2016) cited Swartz (M. S. Swartz & Swanson, 2013); Swartz (M. S. Swartz et al., 2015) cited (Gerbasi et al., 2000); and both Griffith (E. Griffith & Papapietro, 2018) and Zilber (Zilber, 2016) quote an APA position statement from 2015, updated 2020 (APA, 2020). Additionally, Garriga (Garriga et al., 2016) cited Allen (M. H. Allen et al., 2001) for voluntary medication, and Ackerman (Ackerman, 2010) cited (J. W. Parry, 1994) for voluntary commitment.

There are two sources cited twice each by included articles; they did not meet inclusion criteria. Two of Wilk’s papers (Wilk, 1988b, 1988a) quoted Keilitz (Keilitz & Hall, 1985) for involuntary outpatient commitment. Swartz (M. S. Swartz, Swanson, Hiday, et al., 2001) and Pollack (Pollack et al., 2005) both cited Torrey for involuntary outpatient commitment. Four included sources cited four other included sources for definitions, but the definitions were less than similar (N. G. Allen et al., 2015; Compton et al., 2003; Elbogen et al., 2003; Gaynes, 2015; NASMHPD, 2017; M. S. Swartz, Swanson, Wagner, et al., 2001; Wettstein, 1999; Williams, 2022).

# 8. Comparison of key definition words pre-2005 versus post-2005

To broadly test for any possible changes over time, we compared the most frequent (in)voluntary-associated definition words for the 79 sources published 2004 and earlier with the 83 sources published 2005 and afterward. Pre-2005, the most frequent words were: “coercion” (14 sources), comply (9 sources), consent (9 sources), order (8 sources), confine (6 sources), and against the patient’s will/wishes (5 sources). Post-2005, the most frequent definition words were: order (18 sources), against the patient’s will/wishes (11 sources), adhere (10 sources), mandate (9 sources), force (8 mentions), and coercion (6 sources). Coercion, against the patient’s will/wishes, and order were consistently among the six most frequent definitions words. Consent (5 sources post-2005), comply (4 sources post-2005), and confine (5 sources post-2005) were less frequently mentioned post-2005; adhere (3 sources pre-2005), mandate (3 sources pre-2005), and force (5 sources pre-2005) were more frequently mentioned post-2005.

The earliest evidence sources included in this review, published 1966 and 1968, coincide with a transformative period in U.S. mental healthcare (Geller, 2000; George et al., 2023; Pow et al., 2015). First-generation antipsychotics were widely used in the years following their discovery in 1954. The trend in the U.S. towards reducing the hospitalized psychiatric patient population increased in the 1960s, which included the passage of the Community Mental Health Act in 1963. The civil rights of mental health patients received increasing attention, with authors such as Michael Foucault drawing attention to the social control approach to mental illness. Novel mental health interventions were introduced in the 1950s-60s, including general hospital psychiatric units, outpatient clinics, emergency service programs, hospital readmissions, and integration of services across organizations (Geller, 2000). The two definitions we identified from that time period defined involuntary hospitalization and focused on consent and confinement (Leifer, 1966) (Cranton, 1968).

# 9. Term Equivalence for Civil Commitment and Others

In some definitions, the concept of “civil commitment” was explicitly identified as interchangeable with other terms, such as “commitment” (Simon, 2001), “involuntary hospitalization” (Fitch & Ortega, 2000; Leong & Eth, 1991; Mahler & Co, 1984; Simon, 2001), “involuntary psychiatric hold” (Cheung et al., 2018) and “involuntary commitment” (Leibman, 1991) In other cases, “civil commitment” was used interchangeably with other terms but not explicitly stated to be so, for the terms “commitment” (J. W. Parry, 1994). Additionally, “civil commitment” was said to have subtypes depending on the jurisdiction and circumstances. For example, one publication stated that civil commitment was included several “voluntary” and “involuntary” subtypes (J. W. Parry, 1994).

Other terms that were explicitly used interchangeably were “involuntary psychiatric examination” for “a Baker Act examination” (Christy et al., 2003), “involuntary examination” for “emergency commitment” (Christy et al., 2007), “involuntary medication” for “chemical restraints” (S. Hoge et al., 1990), “involuntary commitment” for “therapeutic detention” (McCormack et al., 2013), and “involuntary intervention” for “civil commitment to an inpatient facility and/or forced medication” (Parrish, 1993). In one case, “involuntary psychiatric hospitalization” was defined as “civil commitment of the mentally ill” (Simon, 2001).

# 10. Force and Adhere – Key Definition Words

**Force***:* Force was part of 13 definitions, nearly all of which were involuntary terms. Most commonly, force was used as a verb (“patient forced,” “forcing… to take medication,” “forced to undergo,” “forced,”) to define involuntary civil commitment laws (Brooks, 2007), persons treated involuntarily for mental illness (Charlton, 2006), involuntary commitment (Richmond, 2021), outpatient commitment (Wilk, 1988b, 1988a), civil commitment (“Guidelines for Involuntary Civil Commitment,” 1986), involuntary treatment (Torrey & Stanley, 2013), and psychiatric admission (Alexis, 1986). Force was used as a noun as well, in the cases of “legal force” for involuntary commitment (Lidz et al., 2000), “use of force” for involuntary treatment (Szasz, 2017), “comply or risk force” for outpatient commitment (Wagner et al., 2003). In one case, the adverb form “forcibly remand individuals” was used in the definition of involuntary civil commitment laws (Walton & Hall, 2017). Some special terms were also defined: “involuntary voluntary” patient as someone “actually forced” (Leifer, 2018), and “truly involuntary treatment” as forcing a patient to be treated (Torrey & Stanley, 2013). A “voluntary psychiatric admission” was defined as one without force or provocation (Alexis, 1986).

**Adhere***:* Forms of the word adhere were found in 12 definitions. All 11 involuntary terms were “involuntary outpatient commitment” and had definition words of “order[ed] to adhere” (Skeem et al., 2007; Starks et al., 2022; M. S. Swartz et al., 2015, 2017; M. S. Swartz, Swanson, Wagner, et al., 2001; Zilber, 2016), directed to adhere (M. S. Swartz et al., 2017), “improve adherence” (Swanson et al., 2000; M. S. : W. Swartz H. R. :. Swanson, J. W. :. Hiday, V. A. :. Burns, B. J., 2002), “nonadherent” (M. S. Swartz & Swanson, 2013), and “unlikely to adhere without” (M. S. Swartz et al., 2015). A “voluntary service agreement” was defined signing a statement that “he/she will adhere instead of a mandate” in the context of New York’s assisted outpatient treatment program (Gilbert et al., 2010).

# 11. Discussion of MeSH Terms

One attempt to standardize language in scientific research are controlled vocabularies, which are created and constantly updated to organize, label, and find content in specific context. Each controlled vocabulary term is tightly defined to clearly inform the user, searcher, or indexer what exactly that word or phrase will identify. An example are medical subject heading (MeSH) terms from the National Library of Medicine which include the terms “Involuntary Treatment” and “Involuntary Commitment.” Their definitions mention different concepts, including consent, confinement, and non-voluntariness. Potential questions include what “consent” refers to (the informed consent process, or consent in the general meaning of the term?), and the fact that “involuntary commitment” is circular to the degree that “non-voluntary” is in the definition yet not explained, given arguments by others that the concepts are distinct (Iltis et al., 2023; Sisti, 2017). Furthermore, the involuntary treatment definition describes situations where the patient is *without active agreement*, while the involuntary commitment definition refers to patients who are *actively opposing* or *without active agreement*, two situations with different ethical implications.

**REFERENCES**

Abukamil, R. : M., Douglas. (2017). Vacating an order for civil commitment. *Journal of the American Academy of Psychiatry and the Law*, *45*(4), 493–495.

Ackerman, M. J. (2010). Essentials of forensic psychological assessment, 2nd ed. In *Essentials of psychological assessment series* (2011-12958-000). John Wiley & Sons Inc. https://search.ebscohost.com/login.aspx?direct=true&db=psyh&AN=2011-12958-000&site=ehost-live

Albers, D., Pasewark, R., & Smith, T. (1976). Involuntary hospitalization: The social construction of danger. *American Journal of Community Psychology*, *4*(2), 129–132.

Alexis, A. (1986). Body searches and the right to privacy. *Journal of Psychosocial Nursing and Mental Health Services*, *24*(11), 21–25.

Allen, M. H., Currier, G. W., Hughes, D. H., Reyes-Harde, M., Docherty, J. P., & Expert Consensus Panel for Behavioral Emergencies. (2001). The expert consensus guideline series. Treatment of behavioral emergencies. *Postgraduate Medicine*, *Spec No*, 1–88; quiz 89–90.

Allen, N. G., Khan, J. S., Alzahri, M. S., & Stolar, A. G. (2015). Ethical Issues in Emergency Psychiatry. *Emergency Medicine Clinics of North America*, *33*(4), 863–874. https://doi.org/10.1016/j.emc.2015.07.012

APA. (2018, April 19). Involuntary hospitalization. *APA Dictionary of Psychology, American Psychological Association*. https://dictionary.apa.org/involuntary-hospitalization

APA. (2020, December). *Position statement on involuntary outpatient commitment and related programs of assisted outpatient treatment (APA official actions)*. https://www.psychiatry.org/getattachment/d50db97b-59aa-4dd4-a0ec-d09b4e19112e/Position-Involuntary-Outpatient-Commitment.pdf

Brooks, R. A. (2007). Psychiatrists’ opinions about involuntary civil commitment: Results of a national survey. *Journal of The American Academy of Psychiatry and The Law*, *35*(2), 219–228.

Charlton, M. : F., Terry L. :. Ivandick, Mark J. (2006). Voluntary or Involuntary Receipt of State Services. In *Law & mental health professionals: Colorado.* (2005-14024-008; pp. 421–452). American Psychological Association. https://search.ebscohost.com/login.aspx?direct=true&db=psyh&AN=2005-14024-008&site=ehost-live

Cheung, E. H., Heldt, J., Strouse, T., & Schneider, P. (2018). The Medical Incapacity Hold: A Policy on the Involuntary Medical Hospitalization of Patients Who Lack Decisional Capacity. *Psychosomatics*, *59*(2), 169–176. https://doi.org/10.1016/j.psym.2017.09.005

Christy, A., Bond, J., & Young, M. S. (2007). Short‐term involuntary examination of older adults in Florida. *Behavioral Sciences & the Law*, *25*(5), 615–628. https://doi.org/10.1002/bsl.786

Christy, A., Boothroyd, R. A., Petrila, J., & Poythress, N. (2003). The reported prevalence of mandated community treatment in two Florida samples. *Behavioral Sciences & the Law*, *21*(4), 493–502. https://doi.org/10.1002/bsl.550

Compton, S. N., Swanson, J. W., Wagner, H. R., Swartz, M. S., Burns, B. J., & Elbogen, E. B. (2003). [No title found]. *Mental Health Services Research*, *5*(1), 27–38. https://doi.org/10.1023/A:1021755408267

Cranton, J. R. (1968). Involuntary hospitalization of the mentally ill in Alabama: A critical analysis. *Journal of the Medical Association of the State of Alabama*, *37*(11), 1266–1271.

Davis, G., Lowell, W., & Davis, G. (1998). Determining the number of state psychiatric hospital beds by measuring quality of care with artificial neural networks. *American Journal of Medical Quality : The Official Journal of the American College of Medical Quality*, *13*(1), 13–24.

Drogin, E., & Spaderna, C. (2016). Mental illness, dangerousness, and involuntary commitment. In *Gun violence and mental illness.* (2015-55277-007; pp. 159–183). American Psychiatric Association. https://search.ebscohost.com/login.aspx?direct=true&db=psyh&AN=2015-55277-007&site=ehost-live

Elbogen, E. B., Swanson, J. W., & Swartz, M. S. (2003). Effects of Legal Mechanisms on Perceived Coercion and Treatment Adherence Among Persons with Severe Mental Illness. *Journal of Nervous & Mental Disease*, *191*(10), 629–637. https://doi.org/10.1097/01.nmd.0000092195.22555.12

Fariba, K. : G., Vikas. (2021). *Involuntary Commitment: StatPearls*. http://ovidsp.ovid.com/ovidweb.cgi?T=JS&PAGE=reference&D=medp&NEWS=N&AN=32491309

Fisher, W. H., Barreira, P. J., Lincoln, A. K., Simon, L. J., White, A. W., & Sudders, M. (2001). Insurance status and length of stay for involuntarily hospitalized patients. *Journal of Behavioral Health Services & Research*, *28*(3), 334–346. https://doi.org/10.1007/BF02287248

Fitch, W. L., & Ortega, R. J. (2000). Law and the confinement of psychopaths. *Behavioral Sciences & the Law*, *18*(5), 663–678. https://doi.org/10.1002/1099-0798(200010)18:5<663::AID-BSL408>3.0.CO;2-V

Garakani, A., Shalenberg, E., Burstin, S. C., Brendel, R. W., & Appel, J. M. (2014). Voluntary Psychiatric Hospitalization and Patient-Driven Requests for Discharge: A Statutory Review and Analysis of Implications for the Capacity to Consent to Voluntary Hospitalization. *Harvard Review of Psychiatry*, *22*(4), 241–249. https://doi.org/10.1097/HRP.0000000000000044

Garriga, M., Pacchiarotti, I., Kasper, S., Zeller, S. L., Allen, M. H., Vázquez, G., Baldaçara, L., San, L., McAllister-Williams, R. H., Fountoulakis, K. N., Courtet, P., Naber, D., Chan, E. W., Fagiolini, A., Möller, H. J., Grunze, H., Llorca, P. M., Jaffe, R. L., Yatham, L. N., … Vieta, E. (2016). Assessment and management of agitation in psychiatry: Expert consensus. *The World Journal of Biological Psychiatry*, *17*(2), 86–128. https://doi.org/10.3109/15622975.2015.1132007

Gaynes, B. N. : B., Carrie :. Lux, Linda J. :. Ashok, Mahima :. Coker-Schwimmer, Emmanuel :. Hoffman, Valerie :. Sheitman, Brian :. Viswanathan, Meera. (2015). *Management Strategies To Reduce Psychiatric Readmissions*. http://ovidsp.ovid.com/ovidweb.cgi?T=JS&PAGE=reference&D=medp&NEWS=N&AN=26020093

Geller, J. L. (1986). The Quandaries of Enforced Community Treatment and Unenforceable Outpatient Commitment Statutes. *The Journal of Psychiatry & Law*, *14*(1–2), 149–158. https://doi.org/10.1177/0093185386014001-206

Geller, J. L. (2000). The Last Half-Century of Psychiatric Services as Reflected in Psychiatric Services. *Psychiatric Services*, *51*(1), 41–67. https://doi.org/10.1176/ps.51.1.41

George, P., Jones, N., Goldman, H., & Rosenblatt, A. (2023). Cycles of reform in the history of psychosis treatment in the United States. *SSM - Mental Health*, *3*, 100205. https://doi.org/10.1016/j.ssmmh.2023.100205

Gerbasi, J., Bonnie, R., & Binder, R. (2000). Resource document on mandatory outpatient treatment. *The Journal of the American Academy of Psychiatry and the Law*, *28*(2), 127–144.

Gilbert, A. R., Moser, L. L., Van Dorn, R. A., Swanson, J. W., Wilder, C. M., Robbins, P. C., Keator, K. J., Steadman, H. J., & Swartz, M. S. (2010). Reductions in Arrest Under Assisted Outpatient Treatment in New York. *Psychiatric Services*, *61*(10), 996–999. https://doi.org/10.1176/ps.2010.61.10.996

Griffith, E. E. H., & Etkin, K. (1981). Legal Rights and Involuntary Transfer Following Voluntary Admission. *Psychiatric Services*, *32*(5), 319–322. https://doi.org/10.1176/ps.32.5.319

Griffith, E., & Papapietro, D. (2018). Forensic ethics and involuntary outpatient commitment. In *Ethics challenges in forensic psychiatry and psychology practice.* (2018-10500-008; pp. 116–131). Columbia University Press. https://search.ebscohost.com/login.aspx?direct=true&db=psyh&AN=2018-10500-008&site=ehost-live

Guidelines for involuntary civil commitment. (1986). *Mental and Physical Disability Law Reporter*, *10*(5), 409–514.

Hoge, S., Applebaum, PS, Lawlor, T, Beck, JC, R, L., Greer, A, Gutheil, TG, & Kaplan, E. (1990). A prospective, multicenter study of patients’ refusal of antipsychotic medication. *Archives of General Psychiatry*, *47*(10), 949–956.

Hoge, S. K., Lidz, C. W., Eisenberg, M., Gardner, W., Monahan, J., Mulvey, E., Roth, L., & Bennett, N. (1997). Perceptions of coercion in the admission of voluntary and involuntary psychiatric patients. *International Journal of Law and Psychiatry*, *20*(2), 167–181. https://doi.org/10.1016/S0160-2527(97)00001-0

Hunter, R. H., Ritchie, A. J., & Spaulding, W. D. (2005). The Sell decision: Implications for psychological assessment and treatment. *Professional Psychology: Research and Practice*, *36*(5), 467–475. https://doi.org/10.1037/0735-7028.36.5.467

Iltis, A. S., Fortier, R., Ontjes, N., & McCall, W. V. (2023). Ethics Considerations in Laws Restricting Incapacitated Patients’ Access to ECT. *Journal of the American Academy of Psychiatry and the Law Online*, *51*(1), 47–55. https://doi.org/10.29158/JAAPL.220029-21

Irvin, T. L. (2003). Legal, ethical and clinical implications of prescribing involuntary, life-threatening treatment: The case of the sunshine kid. *Journal of Forensic Sciences*, *48*(4), 856–860.

Johnson, J. M., & Stern, T. A. (2014). Involuntary Hospitalization of Primary Care Patients: (Rounds in the General Hospital). *The Primary Care Companion For CNS Disorders*, *16*(3). https://doi.org/10.4088/PCC.13f01613

Keilitz, I., & Hall, T. (1985). State statutes governing involuntary outpatient civil commitment. *Mental & Physical Disability Law Reporter*, *9*(5), 378–397.

Lareau, C. R. (2013). Civil commitment and involuntary hospitalization of the mentally ill. In *Handbook of psychology: Forensic psychology., Vol. 11, 2nd ed.* (2012-28467-014; pp. 308–331). John Wiley & Sons, Inc. https://search.ebscohost.com/login.aspx?direct=true&db=psyh&AN=2012-28467-014&site=ehost-live

Leibman, F. H. : L., Neil. (1991). Developing trends in prisoners’ rights to mental health treatment. *American Journal of Forensic Psychology*, *9*(1), 19–28.

Leifer, R. (1966). Involuntary psychiatric hospitalization and social control. *The International Journal of Social Psychiatry*, *13*(1), 53–58.

Leifer, R. (2018). Involuntary Psychiatric Hospitalization. *Ethical Human Psychology and Psychiatry*, *19*(3), 182–184. https://doi.org/10.1891/1559-4343.19.3.182

Leong, G. B., & Eth, S. (1991). Ethics in psychiatry: *Current Opinion in Psychiatry*, *4*(6), 872–876. https://doi.org/10.1097/00001504-199112000-00011

Leukefeld, C. G., & Tims, F. M. (1990). Compulsory Treatment for Drug Abuse. *International Journal of the Addictions*, *25*(6), 621–640. https://doi.org/10.3109/10826089009061324

Lidz, C. W., Mulvey, E. P., Hoge, S. K., Kirsch, B. L., Monahan, J., Bennett, N. S., Eisenberg, M., Gardner, W., & Roth, L. H. (2000). Sources of coercive behaviours in psychiatric admissions. *Acta Psychiatrica Scandinavica*, *101*(1), 73–79. https://doi.org/10.1034/j.1600-0447.2000.101001073.x

Mahler, H., & Co, B. T. (1984). Who are the “committed?”: Update. *Journal of Nervous and Mental Disease*, *172*(4), 189–196. https://doi-org.proxy.kib.ki.se/10.1097/00005053-198404000-00001

McCormack, R. P., Williams, A. R., Goldfrank, L. R., Caplan, A. L., Ross, S., & Rotrosen, J. (2013). Commitment to assessment and treatment: Comprehensive care for patients gravely disabled by alcohol use disorders. *The Lancet*, *382*(9896), 995–997. https://doi.org/10.1016/S0140-6736(12)62206-5

Meloy, J. R. : H., Ansar :. Schiller, Eugene F. (1990). Clinical guidelines for involuntary outpatient treatment. In *Practitioner’s resource series* (1990-97292-000). Professional Resource Exchange, Inc. https://search.ebscohost.com/login.aspx?direct=true&db=psyh&AN=1990-97292-000&site=ehost-live

Miller, R. D. (1994). The US supreme court looks at voluntariness and consent. *International Journal of Law and Psychiatry*, *17*(3), 239–252. https://doi.org/10.1016/0160-2527(94)90028-0

Nakic, M. (2017). Civil commitment and involuntary outpatient commitment. In *Psychiatry and the Law: Basic Principles* (pp. 63–73). Springer International Publishing.

NASMHPD. (2017, August). *Trend in psychiatric inpatient capacity, united states and each state, 1970 to 2014*. National Association of State Mental Health Program Directors. https://www.nasmhpd.org/sites/default/files/TACPaper.2.Psychiatric-Inpatient-Capacity_508C.pdf

Parrish, J. (1993). Involuntary use of interventions: Pros and cons. *Innovations & Research*, *2*(1), 15–22.

Parry, J., & Beck, J. (1990). Revisiting the civil commitment/involuntary treatment stalemate using limited guardianship, substituted judgment and different due process considerations: A work in progress. *Mental & Physical Disability Law Reporter*, *14*(2), 102–107.

Parry, J. W. (1994). Involuntary civil commitment in the 90s: A constitutional perspective. *Mental & Physical Disability Law Reporter*, *18*(3), 320–336.

Pinals, D. A. (2016). Treatment engagement, access to services, and civil commitment reform: Would these strategies help reduce firearm-related risks? In *Gun violence and mental illness.* (2015-55277-011; pp. 291–315). American Psychiatric Association. https://search.ebscohost.com/login.aspx?direct=true&db=psyh&AN=2015-55277-011&site=ehost-live

Pollack, D. A., McFarland, B. H., Mahler, J. M., & Kovas, A. E. (2005). Brief Reports: Outcomes of Patients in a Low-Intensity, Short-Duration Involuntary Outpatient Commitment Program. *Psychiatric Services*, *56*(7), 863–866. https://doi.org/10.1176/appi.ps.56.7.863

Pow, J. L., Baumeister, A. A., Hawkins, M. F., Cohen, A. S., & Garand, J. C. (2015). Deinstitutionalization of American Public Hospitals for the Mentally Ill Before and After the Introduction of Antipsychotic Medications. *Harvard Review of Psychiatry*, *23*(3), 176–187. https://doi.org/10.1097/HRP.0000000000000046

Richmond, J. S. (2021). De-escalation in the emergency department. In *Behavioral emergencies for healthcare providers., 2nd ed.* (2021-36863-021; pp. 221–229). Springer Nature Switzerland AG. https://search.ebscohost.com/login.aspx?direct=true&db=psyh&AN=2021-36863-021&site=ehost-live

Serper, M. R., Goldberg, B. R., Herman, K. G., Richarme, D., Chou, J., Dill, C. A., & Cancro, R. (2005). Predictors of aggression on the psychiatric inpatient service. *Comprehensive Psychiatry*, *46*(2), 121–127. https://doi.org/10.1016/j.comppsych.2004.07.031

Simon, R. I. (2001). Concise guide to psychiatry and law for clinicians, 3rd ed. In *Concise guides* (2001-06677-000). American Psychiatric Association. https://search.ebscohost.com/login.aspx?direct=true&db=psyh&AN=2001-06677-000&site=ehost-live

Sisti, D. A. (2017). Nonvoluntary Psychiatric Treatment Is Distinct From Involuntary Psychiatric Treatment. *JAMA*, *318*(11), 999. https://doi.org/10.1001/jama.2017.10318

Skeem, J. L., Louden, J. E., Polaschek, D., & Camp, J. (2007). Assessing relationship quality in mandated community treatment: Blending care with control. *Psychological Assessment*, *19*(4), 397–410. https://doi.org/10.1037/1040-3590.19.4.397

Starks, S. L., Kelly, E. L., Castillo, E. G., Meldrum, M. L., Bourgois, P., & Braslow, J. T. (2022). Client Outreach in Los Angeles County’s Assisted Outpatient Treatment Program: Strategies and Barriers to Engagement. *Research on Social Work Practice*, *32*(7), 839–854. https://doi.org/10.1177/1049731520949918

Stern, T. A., Herman, J. B., & Slavin, P. L. (Eds.). (2004). *The massachusetts general hospital guide to primary care psychiatry*. McGraw-Hill Health Professions Division.

Surya, S., Bishnoi, R. J., & Shashank, R. B. (2019). Balancing Medical Ethics to Consider Involuntary Administration of Electroconvulsive Therapy. *The Journal of ECT*, *35*(3), 150–151. https://doi.org/10.1097/YCT.0000000000000624

Swanson, J. W., Swartz, M. S., Wagner, H. R., Burns, B. J., Borum, R., & Hiday, V. A. (2000). Involuntary out-patient commitment and reduction of violent behaviour in persons with severe mental illness. *British Journal of Psychiatry*, *176*(4), 324–331. https://doi.org/10.1192/bjp.176.4.324

Swartz, M. S., Bhattacharya, S., Robertson, A. G., & Swanson, J. W. (2017). Involuntary Outpatient Commitment and the Elusive Pursuit of Violence Prevention: A View from the United States. *The Canadian Journal of Psychiatry*, *62*(2), 102–108. https://doi.org/10.1177/0706743716675857

Swartz, M. S., Hoge, S. K., Pinals, D. A., Lee, E., Lee, L.-W., Sidor, M., Bell, T., Ford, E., & Scott Johnson, R. (2015). *Resource Document on Involuntary Outpatient Commitment and Related Programs of Assisted Outpatient Treatment*. American Psychiatric Association. https://www.psychiatry.org/getattachment/685f787b-f08f-4b2c-ac4b-35821d50e4fd/Resource-Document-2015-involuntary-outpatient-commitment.pdf

Swartz, M. S., & Swanson, J. W. (2013). Economic Grand Rounds: Can States Implement Involuntary Outpatient Commitment Within Existing State Budgets? *Psychiatric Services*, *64*(1), 7–9. https://doi.org/10.1176/appi.ps.201200467

Swartz, M. S., Swanson, J. W., Hiday, V. A., Wagner, H. R., Burns, B. J., & Borum, R. (2001). A Randomized Controlled Trial of Outpatient Commitment in North Carolina. *Psychiatric Services*, *52*(3), 325–329. https://doi.org/10.1176/appi.ps.52.3.325

Swartz, M. S., Swanson, J. W., Wagner, H. R., Burns, B. J., & Hiday, V. A. (2001). Effects of Involuntary Outpatient Commitment and Depot Antipsychotics on Treatment Adherence in Persons with Severe Mental Illness: *The Journal of Nervous and Mental Disease*, *189*(9), 583–592. https://doi.org/10.1097/00005053-200109000-00003

Swartz, M. S. : W., H. R. :. Swanson, J. W. :. Hiday, V. A. :. Burns, B. J. (2002). The perceived coerciveness of involuntary outpatient commitment: Findings from an experimental study. *Journal of The American Academy of Psychiatry and The Law*, *30*(2), 207–217.

Szasz, T. (2017). Coercion as cure: A critical history of psychiatry. In *Coercion as Cure: A Critical History of Psychiatry* (p. 278). Taylor and Francis. https://www.scopus.com/inward/record.uri?eid=2-s2.0-85065331660&doi=10.4324%2f9781315080994&partnerID=40&md5=bb78cf88f20f0328c37646232cd37ee9

Torrey, E. F., & Stanley, J. (2013). “assisted outpatient treatment”: An example of newspeak?: In reply. *Psychiatric Services (Washington, D.C.)*, *64*(11), 1179–1180. https://doi.org/10.1176/appi.ps.641109

Wagner, H. R., Swartz, M. S., Swanson, J. W., & Burns, B. J. (2003). Does involuntary outpatient commitment lead to more intensive treatment? *Psychology, Public Policy, and Law*, *9*(1–2), 145–158. https://doi.org/10.1037/1076-8971.9.1-2.145

Walton, M. T., & Hall, M. T. (2017). Involuntary Civil Commitment for Substance Use Disorder: Legal Precedents and Ethical Considerations for Social Workers. *Social Work in Public Health*, *32*(6), 382–393. https://doi.org/10.1080/19371918.2017.1327388

Wasser, T. (2017). Conclusion: How to learn more about forensic psychiatry. In *Psychiatry and the Law: Basic Principles* (pp. 183–188). Springer International Publishing. https://www.scopus.com/inward/record.uri?eid=2-s2.0-85043334567&doi=10.1007%2f978-3-319-63148-6_16&partnerID=40&md5=acef3bb30638681e701e453193e83bba

Wettstein, R. M. (1999). THE RIGHT TO REFUSE PSYCHIATRIC TREATMENT. *Psychiatric Clinics of North America*, *22*(1), 173–182. https://doi.org/10.1016/S0193-953X(05)70067-8

Wilk, R. J. (1988a). Implications of involuntary outpatient commitment for community mental health agencies. *American Journal of Orthopsychiatry*, *58*(4), 580–591.

Wilk, R. J. (1988b). Involuntary Outpatient Commitment of the Mentally III. *Social Work*, *33*(2), 133–137. https://doi.org/10.1093/sw/33.2.133

Williams, S. N. : J., Melissa G. (2022). Inpatient treatment for African American women. In *A handbook on counseling African American women: Psychological symptoms, treatments, and case studies.* (2022-31334-011; pp. 209–226). Praeger/ABC-CLIO. https://search.ebscohost.com/login.aspx?direct=true&db=psyh&AN=2022-31334-011&site=ehost-live

Zilber, C. (2016). Ethics Considerations of Involuntary Outpatient Treatment. *Psychiatric News*, *51*(23), 1–1. https://doi.org/10.1176/appi.pn.2016.12a16
